# Unveiling the True Identity of Carborane-Fused Phosphorus Heterocycles

**DOI:** 10.1021/acs.orglett.5c01374

**Published:** 2025-05-26

**Authors:** Dalma Gál, Lóránt Szántai, Dániel Buzsáki, Zsolt Kelemen

**Affiliations:** † Department of Inorganic and Analytical Chemistry, 61810 Budapest University of Technology and Economics, Műegyetem Rkp. 3, 1111 Budapest, Hungary; ‡ Wigner Research Centre for Physics, P.O. Box 49, H-1525 Budapest, Hungary; § HUN-REN Computation Driven Chemistry Research Group, 61810 Budapest University of Technology and Economics, Műegyetem Rkp. 3, 1111 Budapest, Hungary

## Abstract

In recent years, the possible global 2D/3D aromaticity of carborane-fused cycles has been widely debated. While phosphorus heterocycles fused with carboranes are known, the 2D aromatic character in carborane-fused phospholes was only recently reported. However, our computational study found that these compounds lack 2D aromaticity. Instead, they should be viewed not as a fusion of an aromatic ring but as a fusion of an unsaturated ring with carboranes.

## Graphical abstract

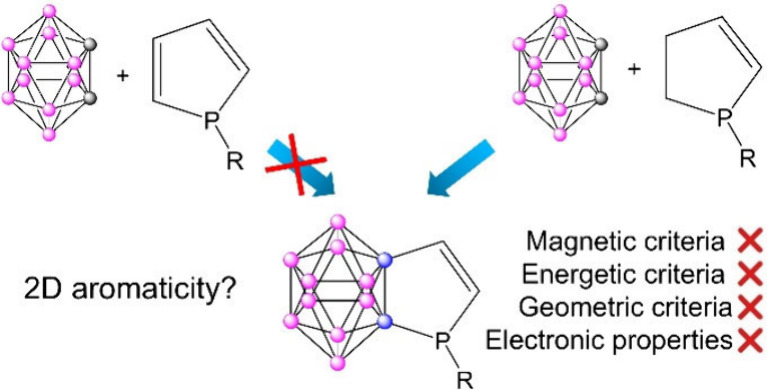

Over the past decade, the relationship between 2D and 3D aromaticity—and the potential for aromatic conjugation between them—has been the subject of intensive research.^1–7^ Carborane-fused cycles (representative examples **I**–**VI** in Scheme 1) have likely been among the most extensively studied systems in this context (Scheme 1). Despite promising initial results,^8–10^ recent studies have demonstrated that no aromatic conjugation exists between these two types of aromatic systems.^11–15^ In general, 3D/2D aromatic fusion is not feasible due to the poor overlap between the π molecular orbitals of the planar unit and the (n+1) molecular orbitals of the aromatic cage, as demonstrated by Poater, Teixidor, and Solà.^11^ This limited interaction prevents effective electronic delocalization across the fused units. In the case of carborane-fused heterocycles, the position of the heteroatom within the *exo* ring dictates the bonding pattern.^12^ In certain cases, the system actively prevents conjugation by any means, directing its electrons toward the unfused carbon atoms, and even prioritizing the triplet state compared to the singlet. Although the investigations of the aromaticity of the 2D fused ring using the readily applicable NICS indices^16^ were considered significant,^8–10^ their conclusions have since been proven incorrect. The magnetic field induced by the 3D cluster affects the magnetic properties of the fused *exo* ring, thus potentially leading to a misinterpretation of its aromatic character.^8–10^ We have demonstrated that the overall stability of the fused system can be ruled by negative hyperconjugation and ring strain and strongly depends on the connection side of the carborane.^13^ However, no aromatic conjugation exists in the case of furan-, thiophene-, and indole-fused systems. Very recently, Poater extended this group by investigating pyrazole- and pyrazoline-based systems^14^ and several boracycles,^15^ establishing the same conclusion.

**Scheme 1:**
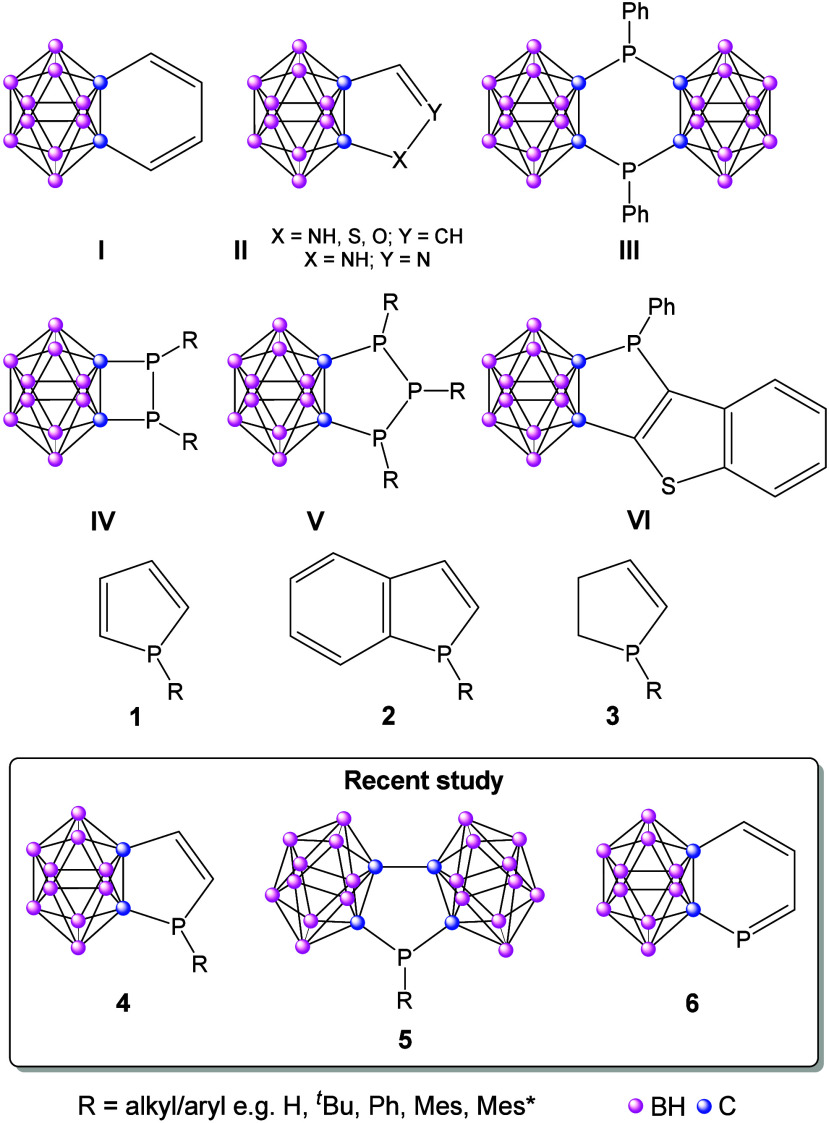
*o*-Carborane-Fused Ring Systems and the Parent Phosphole and Phospholene Derivatives

Phosphorus heterocycles, especially phospholes and phosphabenzenes, have been deeply investigated during the past several decades, due to their tunable electronic properties.^17–19^ The parent phosphole, owing to the pyramidal configuration of the phosphorus atom, exhibits only weak aromatic character. However, forcing the planarization with the addition of bulky substituents can result in systems with increased aromatic properties.^19^ For example, the tri-*tert*-butylphenyl (supermesityl (Mes*)) derivative exhibits comparable aromaticity to the pyrrole.^20^ Certainly, several *o*-carborane-fused phosphorus heterocycles have garnered increasing attention since the early days of carborane research.^21^ Various carborane-fused ring systems have been synthesized (some representative examples (**III**–**VI**) are shown in Scheme 1);^22–29^ however, no aromatic conjugation had been proposed until now. Very recently, Wang and Duan synthesized *o*-carborane-fused phosphorus heterocycles (**VI**) and stated that these compounds have weak but higher aromatic character compared to the corresponding benzophosphole derivatives.^30^ This finding is in full contradiction with the earlier observation made for other five-membered heterocycles. This raises an intriguing question: What is the origin of this apparent discrepancy? Can phosphorus heterocycles be the missing puzzle piece to achieve global 3D/2D aromaticity within carborane-fused heterocycles?

Although aromaticity cannot be directly measured, several subcriteria contribute to its overall assessment.^31^ In order to better judge the aromaticity, a set of indicators based on various properties is worth investigating.^31–33^ Among the various theoretical approaches, calculations of the nucleus-independent chemical shift (NICS) are among the most widely used descriptors of aromaticity due to its facile applicability.^34^ The aromaticity of carborane-fused systems was investigated by NICS values, as well;^8–10,30^ however, it was demonstrated in multiple cases that this method cannot be simply used as a black-box technique.^35–38^ Since the magnetic field induced by the 3D carborane moiety has a noticeable impact in a huge radius around the cluster, it practically affects the magnetic properties of the 2D fused ring.^12,13^ Moreover, this phenomenon is more effective in the geometrical center of the smaller five-membered *exo* rings (where NICS(0) is computed) due to their relative closeness to the carborane cage.^12^ In order to get comprehensive results (for computational details, see the Supporting Information), we have investigated systems **4**–**6** (Scheme 1); moreover we have reinvestigated several already synthesized compounds (**1**–**3**). We have varied the size of the R substituents, too (R = H, ^*t*^Bu, Ph, tri-*tert*-methylphenyl (Mes), or tri-*tert*-butylphenyl (Mes*)), as it is well-known that the pyramidalization of the phosphorus atom significantly influences the aromatic character of phosphorus heterocycles.^16^ In order to judge the magnetic shielding of the parent carborane, it is worth investigating the magnetic shielding of ghost atoms along from the perpendicular bisector line of the C–C bond of the carborane (at the B3LYP/cc-pVTZ//B3LYP/6-311+G** level of theory).^12^ In the case of **4** (R = H), the ghost atom is around 1.11 Å from the center of the C–C carborane bond. In this position, the NICS(0) of the parent carborane is around −6.5 ppm, which is even lower than the values obtained by Wang and Duan (−4.1 and −4.2 ppm at the B3LYP/6-31G** level of theory).^30^ Accordingly, the magnetic shielding of the parent carborane strongly affects the NICS values similar to the case of other heterocycles; indeed, this method is not suitable for the assessment of aromatic nature. Nevertheless, we have calculated the corresponding NICS(0) values of **4** and **5** to compare them with those of **1**–**3** (Table 1). In the case of **4**, the NICS(0) values are overall less affected by the planarity of the phosphorus atom, even if the steric bulk at the phosphorus center is increased (the difference between the extremes is 0.6 ppm), indicating distinct behavior compared to aromatic **1** and **2**, while similar to partially saturated **3**. These findings suggest that **4** is better classified as a phospholene rather than a phosphole. The more negative NICS(0) values of **5** can be explained by the presence of two carborane units, which contribute to the overall magnetic shielding in an additive manner. In order to gain better understanding of the magnetic properties, the SYSMOIC package was applied, which is based on the continuous transformation of the origin of the current density method.^39^ It defines an external magnetic field from a certain direction and measures the induced bond currents. This method showed that the diatropic bond currents are diminished in the case of carborane-fused systems compared to pyrrole and phosphole, indicating no aromatic character (Figure S1 and Table S1).

**Table 1:** Computed NICS(0) Values at the B3LYP/cc-pVTZ/B3LYP/6-311+G** Level of Theory

	**1**	**2**	**3**	**4**	**5**
R = H	–5.3	–2.2	–3.5	–5.4	–8.3
R = ^*t*^Bu	–6.0	–2.5	–3.5	–6.0	–8.3
R = Ph	–4.8	–1.4	–3.5	–5.5	–7.9
R = Mes	–7.3	–3.3	–3.1	–5.4	–7.9
R = Mes*	–9.6	–5.4	–3.1	–5.8	–7.8

Moreover, the carborane C–C bond current turned out to be net paratropic, therefore interrupting the flow within the *exo* ring. We can conclude that highly negative magnetic shielding does not depict aromatic properties, since no circular 2D magnetic current is induced in the center of the *exo* ring in **4**–**6**.

The evaluation of the magnetic criteria is only one of many for aromaticity;^31–33,40^ therefore, in the next step, aromatic stabilization was investigated by isodesmic reactions (equations **I** and **II** in Figure 1). Both investigated isodesmic reactions are endothermic, indicating significantly less aromatic character than the reference structures. The reaction energies slightly increase with the size of the steric bulk, in agreement with the planarization of the phosphorus atom, resulting in a higher aromatic character of the reference phosphole system. This aromatic stabilization diminishes upon fusion with the carborane cluster; consequently, the reactions become more endothermic. In the case of fused phosphabenzene derivatives, the same conclusion can be reached (Figure S2). In the case of aromatic systems containing σ,^3^λ^3^-pnictogen atoms, the inversion barrier is usually a good indicator of the aromaticity/conjugation.^17,41^ The computed values for systems **4** and **5** are significantly higher than the parent phosphole (**1**) and benzophosphole (**2**) derivatives (Figure 2); however, they are in agreement with the partially saturated phospholene derivatives (**3**). It is important to highlight that the investigation of isomer stabilization energies (Figure S3 and Table S2) further bolstered the similarity of **4** to partially saturated 2-phospholene derivatives (**3**), as both systems exhibit very similar isomer stabilization energies. This observation aligns well with the established understanding of other five-membered heterocycles fused with *o*-carborane.^12^ In view of these data, these compounds should be classified as carborane-fused phospholene and not as carborane-fused phosphole derivatives. This conclusion can be extended to other heterocycles, as well. This statement is entirely consistent with the bonding characteristics of the parent *o*-carborane compared to *o*-carboryne, which can be considered as the unsaturated analogue of *o*-carborane. In *o*-carborane and its derivatives, the C–C bond should be regarded as a single bond, and C=C bond character appears in the case of *o*-carboryne.^42^

**Figure 1:**
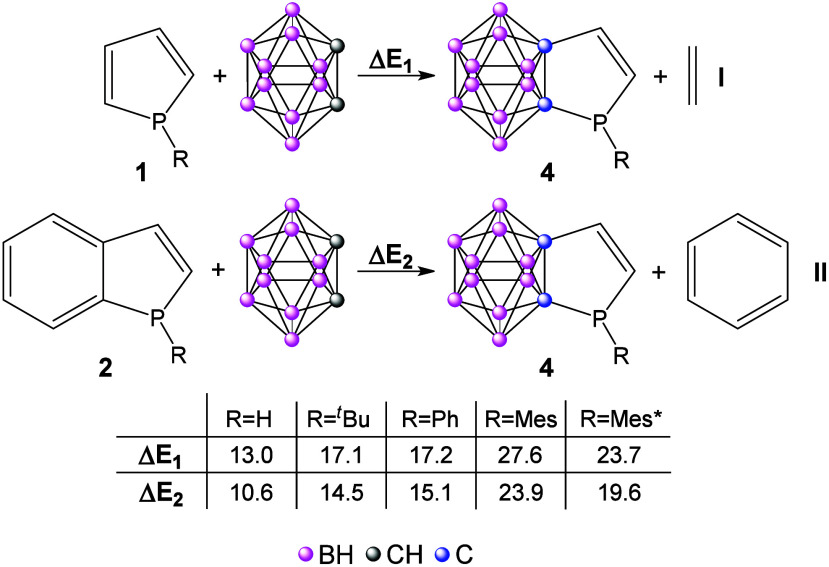
Different reactions that evaluate aromatic stabilization.

**Figure 2:**
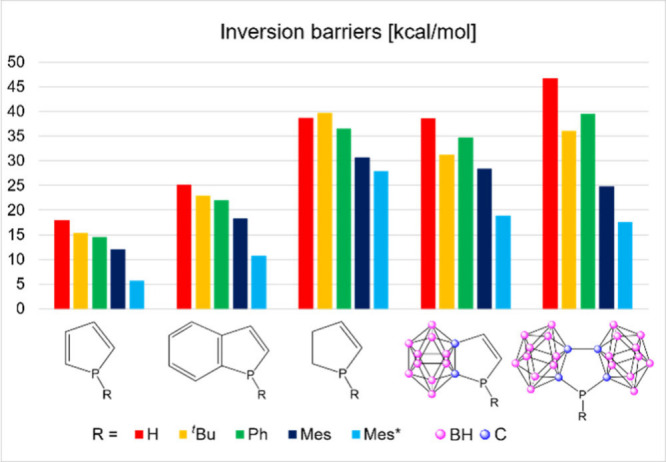
Inversion barriers (*E* in kcal/mol) of **1–5** with different R substituents.

The third criterion for aromaticity that was examined consisted of the geometric parameters. Commonly used geometric aromaticity indices, such as the Bird index,^43^ which are designed to characterize the aromatic properties of 2D systems, cannot be applied to carboranes due to carbon–carbon cluster bonds being longer than a standard C–C single bond.^12,13^ On the other hand, investigating the geometric parameters of the fused ring may deliver important information (Scheme 2). In the case of parent phosphole derivatives, as the steric bulk increases, the bond lengths become more equalized, and the deviation of the bond lengths compared to the reference structures decreases, in agreement with earlier findings (more information in Table S3).^17,44^ In the case of **4**, the deviation becomes much greater, indicating no aromatic conjugation. Increasing the size of the steric bulk at the phosphorus atom does not lead to bond length equalization. In contrast, replacing the hydrogen substituent at phosphorus with Mes* in **4** results in further elongation of the P–C bonds, while the *exo* C=C bond becomes shorter (the deviation is not decreasing in parallel with the increment of the steric bulk (see Table S3)). In the case of **6**, similar statements can be made; the bonds between the carborane cluster and the *exo* atoms have single bond character.

**Scheme 2:**
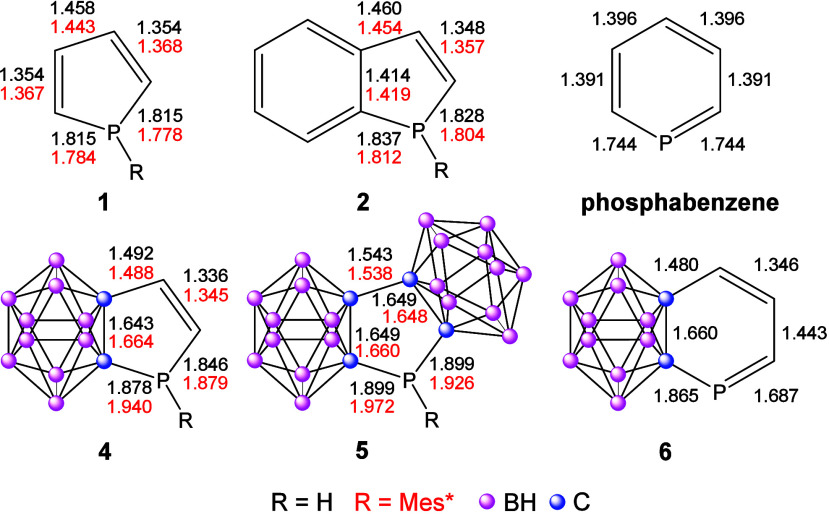
Bond Lengths in Angstroms of **1**, **2**, **4–6**, and Phosphabenzene (R = H and R = Mes*, at the B3LYP/6-311+G** level of theory)

Today, the utilization of electronic indices via the investigation of electron delocalization has become quite popular. The normalized multicenter delocalization index^45,46^ (MCI^1/*n*^) has been applied for carborane-fused systems, as well.^13^ Table S4 shows that MCI^1/*n*^ values of the carborane-phosphole fused system (**4** and **5**) are mostly comparable to the corresponding phospholene analogue (**3**), therefore indicating non-aromatic character. Higher aromatic properties can be seen in the case of phosphabenzene-fused systems; however, the phosphacyclohexadiene system also showed similar MCI^1/*n*^ values (0.36).

The amount of cyclically delocalized electrons in the electron density of delocalized bonds’ local EDDB_P_ function within the *exo* rings shows similar trends compared to the MCI values (Table S4). By visualizing the global EDDB_H_ function, we can see the very low degree of aromatic character of the parent phosphole (**1** (Figure 3)), which totally decreases in case of the carborane-fused systems (**4** and **5**). The difference is even more striking in the case of phosphabenzene. Phosphabenzene exhibits significant 2D aromatic character, which is completely lost upon fusion with the carborane cluster, becoming similar to that of phosphacyclohexadiene. The delocalization properties of the carborane system do not extend to the fused ring in the case of **4**–**6** and, therefore, do not induce 2D aromaticity (Figure 3 and Figures S4 and S5).

**Figure 3:**
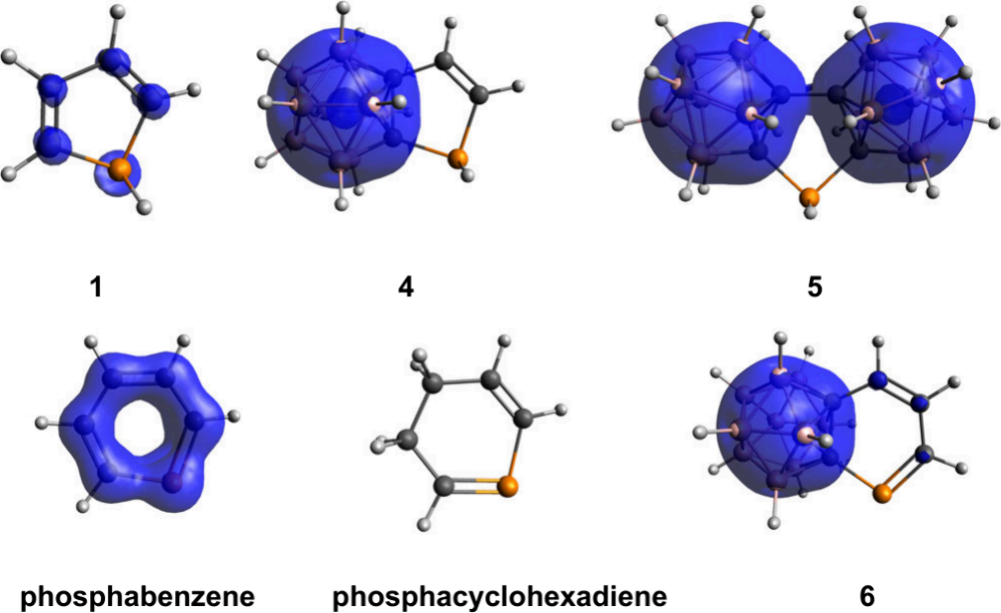
Total electron density (isovalue surface of 0.015) of the EDDB_H_ function of the investigated systems of **1**, **4–6**, phosphabenzene, and phosphacyclohexadiene. The same plot for the other compounds can be found in Figures S4 and S5.

In conclusion, our in-depth computational study demonstrates that phosphorus heterocycles fused with *o*-carborane neither exhibit 2D aromaticity nor show any 2D–3D aromatic conjugation between the planar ring and the three-dimensional carborane cluster. The previously reported aromatic character of carborane-fused phosphorus heterocycles appears to be overestimated,^30^ largely due to misleading NICS values. Moreover, we clearly show that the electronic nature of these fused systems resembles more closely the partially saturated compounds, such as phospholene. This observation can also be extended to other heterocycles fused with carboranes. These results further expand our understanding of 2D–3D carborane-fused systems and their potential for electronic modulation in various applications.

## Supplementary Material



## Data Availability

The data underlying this study are available in the published article and its Supporting Information.

## Notes

Computational details, XYZ geometries, and total energies of the investigated systems (PDF)
