# Supplementary material for: Unveiling the True Identity of Carborane-Fused Phosphorus Heterocycles
Source: Org Lett. 2025 May 26;27(22):5637–41. doi: 10.1021/acs.orglett.5c01374 (PMC12150315; doi:10.1021/acs.orglett.5c01374)
Supplement: Supplementary file 1 [file ol5c01374_si_001.pdf]

Unveiling the True Identity of Carborane-Fused Phosphorus Heterocycles

Supplementary Information

Dalma Gál,[a] Lóránt Szántai,[a] Dániel Buzsáki,[b] Zsolt Kelemen*[a,c]

a) Department of Inorganic and Analytical Chemistry, Budapest University of Technology and Economics, Műegyetem Rkp. 3, 1111 Budapest, Hungary, e-mail: kelemen.zsolt@vbk.bme.hu

b) Wigner Research Centre for Physics, P.O. Box 49, H-1525 Budapest, Hungary

c) HUN-REN Computation Driven Chemistry Research Group, Budapest University of Technology and Economics, Műegyetem Rkp. 3, 1111 Budapest, Hungary

Table of Contents

Computation details S1
Figure S1 ....S2
Table S1 ....S3
Figure S2 ....S3
Discussion of the obtained isomer stabilization energies S4
Figure S3 ....S4
Table S2 ....S5
Table S3 ....S5
Table S4 ....S5
Figure S4 ....S6
Figure S5 ....S7
References S8
XYZ Geometries and Total Energies of the Investigated Systems S9

Computation details

All DFT calculations were performed using the Gaussian 16 program package.[1] Full geometry optimization was performed for all molecules at the B3LYP/6-311+G** level of theory. B3LYP was successfully applied for several related systems.[2–6] Single-point calculations were carried out at the B3LYP/cc-pVTZ level of theory to evaluate the NICS values. All computations were performed using Gaussian 16 program with default convergence criteria. Magnetic bond current strengths were computed and plotted with the BOCUST and v3d programs of the SYSMOIC[7] package. The determination of the natural orbitals for the calculation of the electronic properties was performed using the NBO 7.0[8] program. These results were processed by the Multiwfn[9] software to gain $MCI^{1/n}$ values and by the RunEDDB script to gather electron density of delocalized bonds (EDDB) electron densities.[10] Since it was shown that the raw value of MCI cannot be used in case of rings with different sizes[11], they are normalized before application to get $MCI^{1/n}$, where *n* is defined as the number of atoms in the ring. In case of EDDB method, the electron density is separated into one-center and two-center localized parts, and the remaining population is defined as EDDB.[10,12] $EDDB_P$ function can be used for individual rings, while $EDDB_H$ shows the global density with the exclusion of hydrogens. $EDDB_H$ plots were generated with Avogadro.[13]

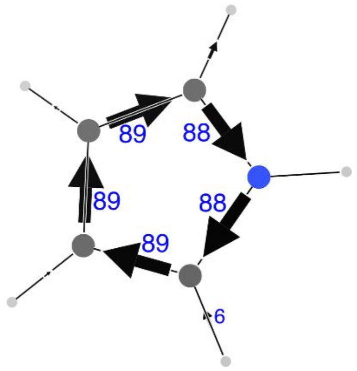

pyrrole

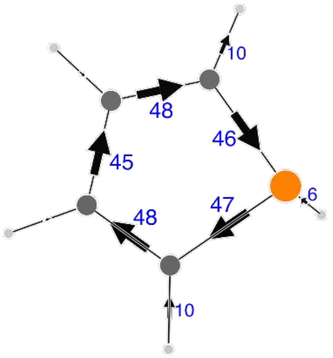

1

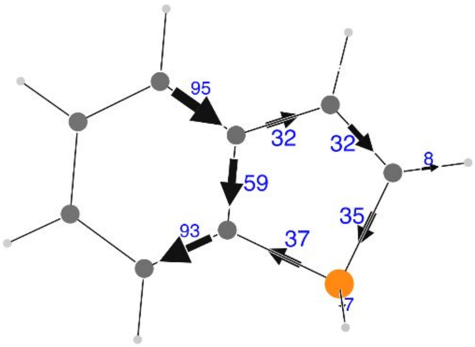

2

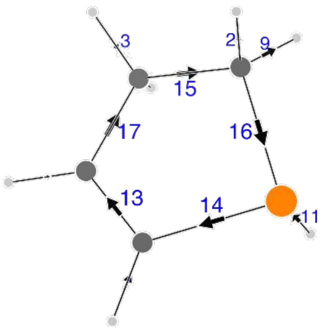

3

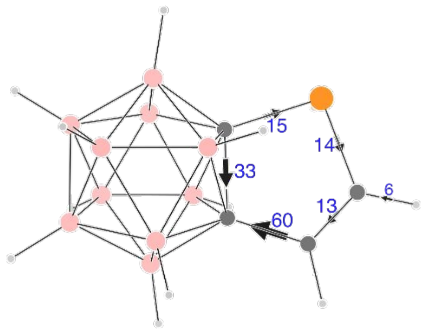

4

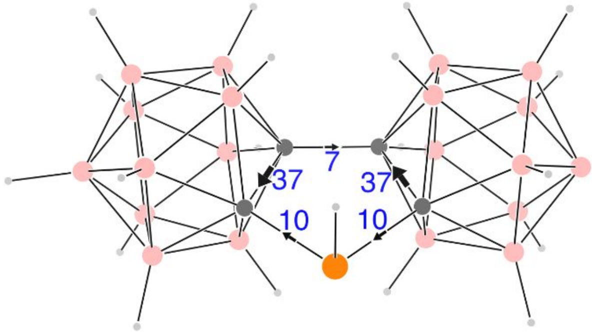

5

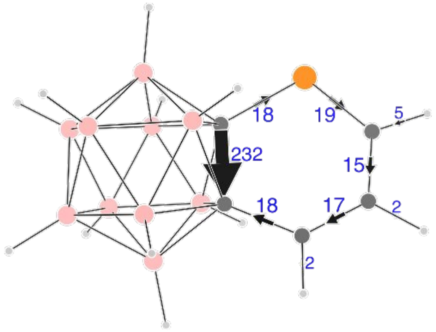

6

Figure S1 The bond current strengths of the investigated systems while inducing them with a magnetic current perpendicular to the molecular plane of the 2D fused moieties. The direction of the arrows shows the character of the bond current (diatropic for clockwise, paratropic for counter-clockwise), while the numbers show the percentage of the absolute value of the combined diatropic and paratropic currents compared to current strengths of individual bonds in benzene (12.0 nA/T).

Table S1 The sum of the diatropic (negative) and paratropic (positive) current strengths (in nA/T) of **1-5** while inducing them with a magnetic current perpendicular to the molecular plane of the 2D rings (by using Continuous Set of Gauge Transformations (CSGT)[14] method at the B3LYP/6-311+G** level of theory). For pyrrole, all current strengths are -10.55 nA/T.

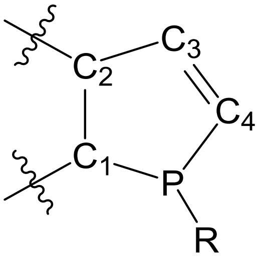

	**P-C_1**	**C_1-C_2**	**C_2-C_3**	**C_3-C_4**	**C_4-P**
**1_H**	-5.69	-5.76	-5.44	-5.74	-5.55
**2_H**	-4.49	7.12	-3.88	-3.82	-4.16
**3_H**	-1.90	-1.80	-2.01	-1.51	-1.65
**4_H**	-1.67	-1.52	-7.24	3.98	-1.84
**5_H**	-1.14	4.43	-0.81	4.50	-1.14

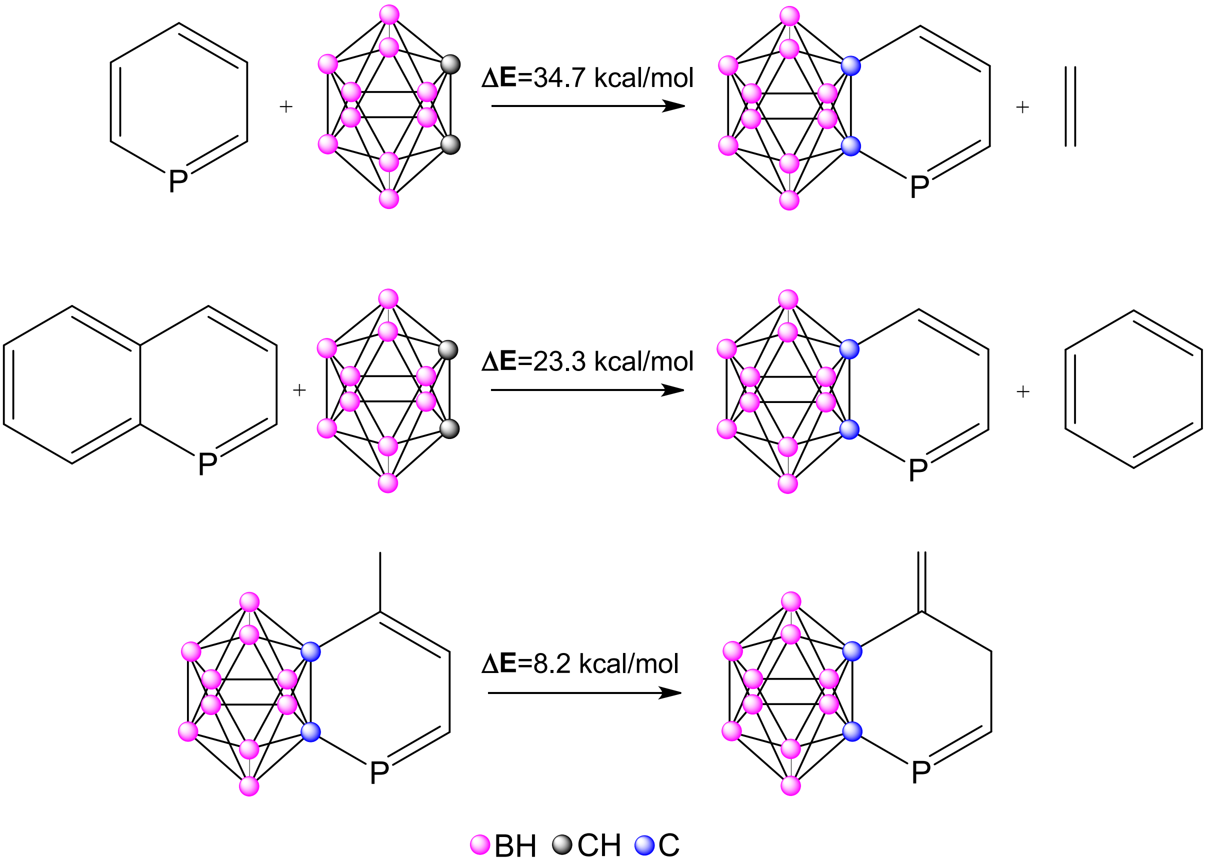

Figure S2 Reactions to evaluate the aromaticity of phosphabenzene derivatives (B3LYP/6-311+G**)

Discussion of the obtained isomer stabilization energies

Interestingly, the parent phosphole derivatives exhibit very similar values ($\Delta ISE_{phosphole}$, **Figure S3 and Table S2**), suggesting a comparable aromatic character in the case of carborane-fused systems (ΔISE). In contrast, investigating the isomer stabilization energy of the partially saturated 2-phospholene ($\Delta ISE_{phospholene}$) derivatives yields the same values, indicating that the stabilization energy reflects the conjugation between the phosphorus lone pair and the double bond and not the aromatic stabilization. This observation aligns well with the established understanding of other five-membered heterocycles fused with *o*-carborane.

Alternative ISE reactions (labelled by ‘) were investigated as well, where the methyl group is in position 2. For the phosphole derivatives these reactions are more endothermic compared to the 3-methyl analogues, as the two double bonds cannot conjugate directly, only through the phosphorus atom. In the case of phospholene and carborane derivatives, the dominant interaction involves the phosphorus lone pair and the adjacent double bond. This interaction is retained after proton migration, allowing the phosphorus lone pair to conjugate with the exocyclic double bond, therefore similar values were obtained in all cases.

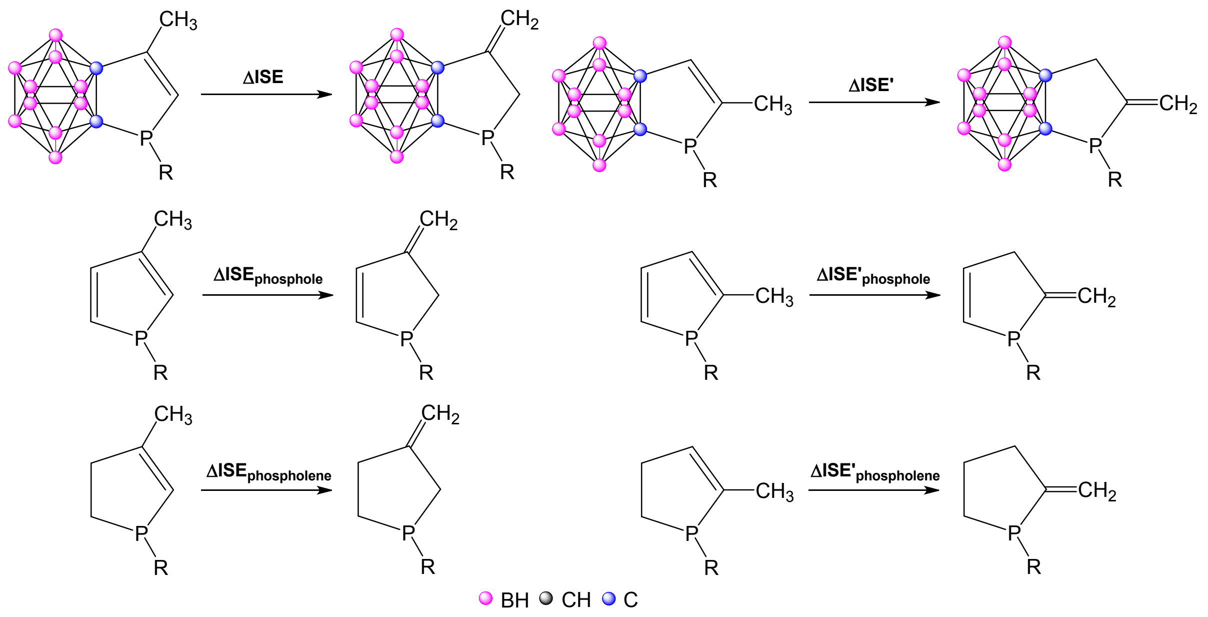

Figure S3 Reactions to evaluate isomer stabilization energy

Table S2 Computed isomer stabilization energy at B3LYP/6-311+G** level of theory

	**ΔISE**	**$\Delta ISE_{phosphole}$**	**$\Delta ISE_{phospholene}$**	**ΔISE'**	**$\Delta ISE'_{phosphole}$**	**$\Delta ISE'_{phospholene}$**
R=H	5.0	4.7	2.9	3.2	9.5	3.3
R=^{t}Bu	4.9	5.0	5.1	4.2	8.7	3.1
R=Ph	6.2	3.7	4.9	4.0	9.3	4.8
R=Mes	6.7	6.7	5.9	3.7	11.2	4.3
R=Mes*	8.9	9.6	8.0	4.8	13.3	5.3

Table S3 Standard deviation of the bond lengths of the *exo* ring compared to a reference structure. The reference structure is defined as a hypothetical phosphole ring, in which bonds are defined as: $d_{X\text{-}Y(opt)}=(d_{X\text{-}Y}+2*d_{X=Y})/3$, where $d_{X\text{-}Y}$ is the smallest single bond reference (e.g. ethane or phosphaethane), while $d_{X=Y}$ is the smallest double bond reference (e.g ethene or phosphaethene). All structures were calculated at the B3LYP/6-311+G** level of theory.

**1**$_{H}$	0.052	**2**$_{H}$	0.060	**3**$_{H}$	0.109	**4**$_{H}$	0.136	**5**$_{H}$	0.153
**1**$_{tBu}$	0.048	**2**$_{tBu}$	0.058	**3**$_{tBu}$	0.106	**4**$_{tBu}$	0.140	**5**$_{tBu}$	0.153
**1**$_{Ph}$	0.051	**2**$_{Ph}$	0.060	**3**$_{Ph}$	0.107	**4**$_{Ph}$	0.138	**5**$_{Ph}$	0.159
**1**$_{Mes}$	0.042	**2**$_{Mes}$	0.054	**3**$_{Mes}$	0.106	**4**$_{Mes}$	0.138	**5**$_{Mes}$	0.168
**1**$_{Mes*}$	0.030	**2**$_{Mes*}$	0.046	**3**$_{Mes*}$	0.107	**4**$_{Mes*}$	0.163	**5**$_{Mes*}$	0.180

Table S4 $MCI^{1/n}$ and the amount of cyclically delocalized electrons in the $EDDB_P$ function of the investigated systems in the fused 2D ring

	**R**	**1**	**2**	**3**	**4**	**5**	**6**
$MCI^{1/n}$	H	0.51	0.44	0.30	0.31	0.26	0.37
	Ph	0.51	0.44	0.31	0.31	0.26	
$EDDB_P$	H	0.52	0.40	0.15	0.19	0.17	0.36
	Ph	0.42	0.35	0.15	0.23	0.22	

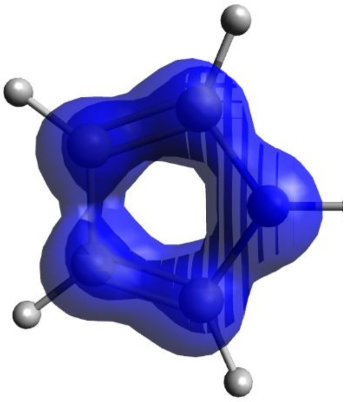

pyrrole

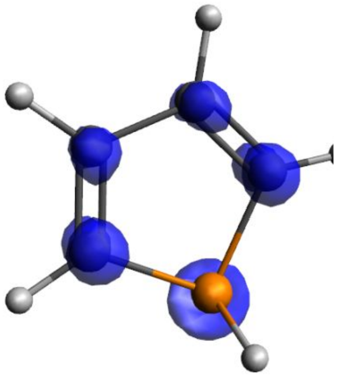

1

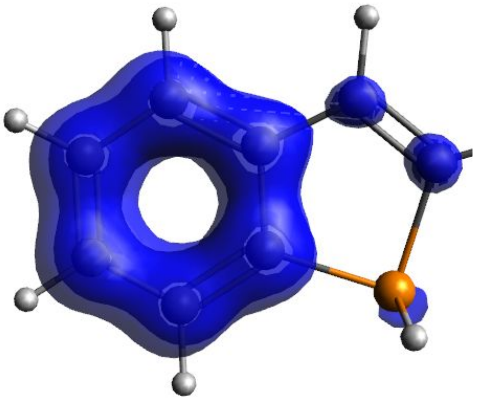

2

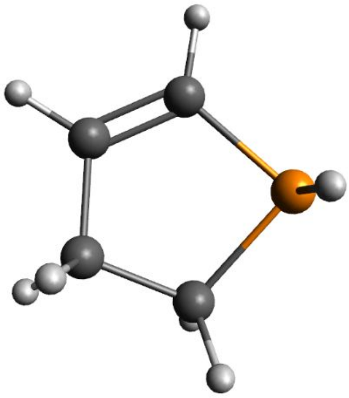

3

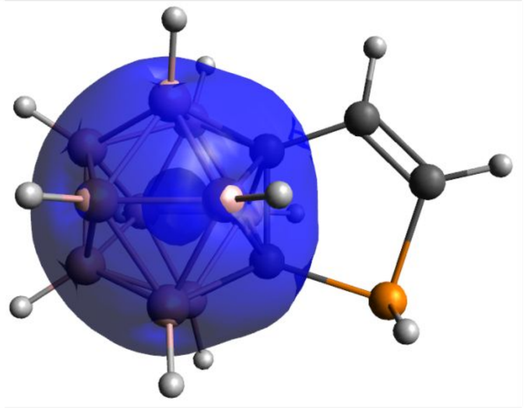

4

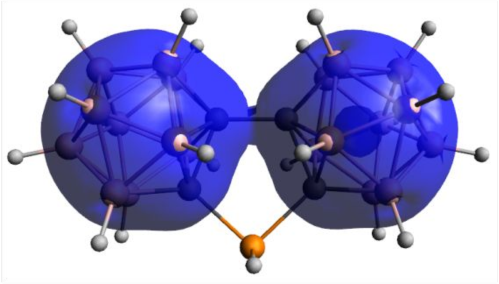

5

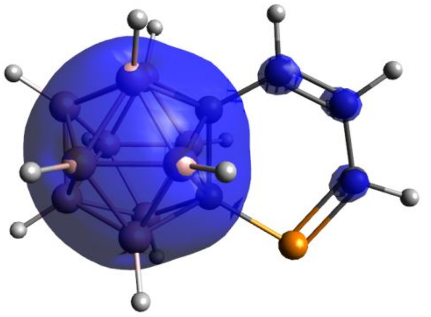

6

Figure S4 The total electron density (0.015 isovalue surface) of the $EDDB_H$ function of the investigated systems (R=H)

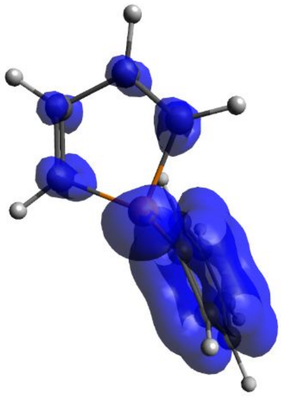

1

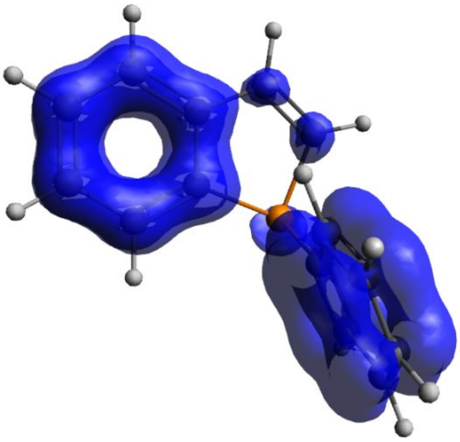

2

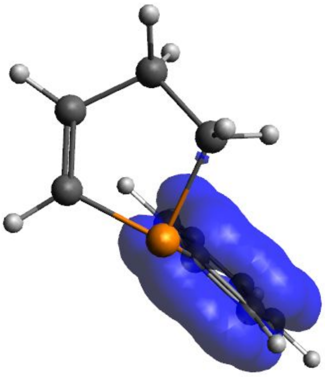

3

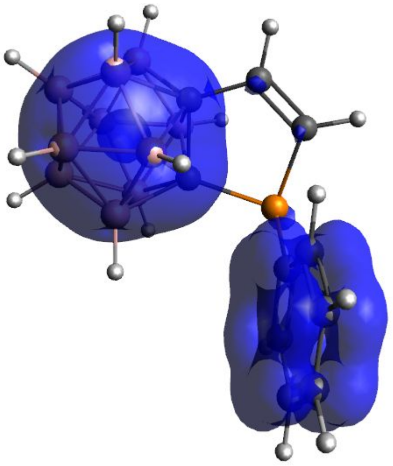

4

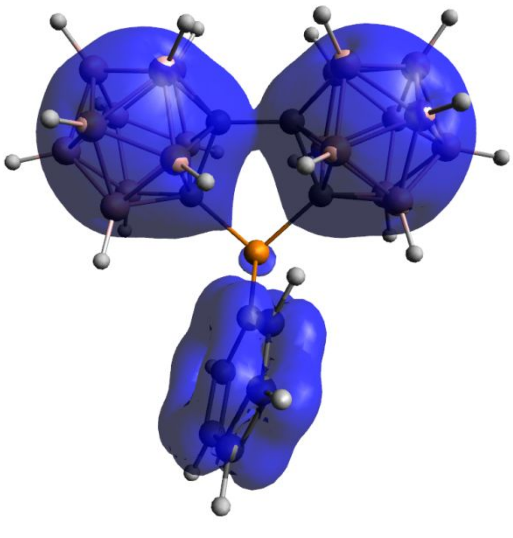

5

Figure S5 The total electron density (0.015 isovalue surface) of the $EDDB_H$ function of the investigated systems (R=Ph)

References

(1) Frisch, M. J.; Trucks, G. W.; Schlegel, H. B.; Scuseria, G. E.; Robb, M. A.; Cheeseman, J. R.; Scalmani, G.; Barone, V.; Petersson, G. A.; Nakatsuji, H.; Li, X.; Caricato, M.; Marenich, A. V.; Bloino, J.; Janesko, B. G.; Gomperts, R.; Mennucci, B.; Hratch, J. B. ; F. Gaussian 16, Revision C.01, 2016.

(2) Buzsáki, D.; Kovács, M. B.; Hümpfner, E.; Harcsa-Pintér, Z.; Kelemen, Z. Conjugation between 3D and 2D Aromaticity: Does It Really Exist? The Case of Carborane-Fused Heterocycles. *Chem. Sci.* **2022**, *13* (38), 11388–11393.

(3) Buzsáki, D.; Gál, D.; Harcsa-Pintér, Z.; Kalabay, L.; Kelemen, Z. The Possible Aromatic Conjugation via the Different Edges of (Car) Borane Clusters: Can the Relationship Between 3D and 2D Aromatic Systems Be Reconciled? *Chem. Eur. J.* **2024**, *30* (61), e202402970.

(4) El Bakouri, O.; Szczepanik, D. W.; Jorner, K.; Ayub, R.; Bultinck, P.; Solà, M.; Ottosson, H. Three-Dimensional Fully π-Conjugated Macrocycles: When 3D-Aromatic and When 2D-Aromatic-in-3D? *J. Am. Chem. Soc.* **2022**, *144* (19), 8560–8575. https://doi.org/10.1021/jacs.1c13478.

(5) Poater, J.; Solà, M.; Viñas, C.; Teixidor, F. π Aromaticity and Three-Dimensional Aromaticity: Two Sides of the Same Coin. *Angew. Chem. - Int. Ed.* **2014**, *53* (45), 12191–12195. https://doi.org/10.1002/anie.201407359.

(6) Buzsáki, D.; Gál, D.; Szathmári, B.; Holczbauer, T.; Udvardy, A.; Szilágyiné, J. K.; Kargin, D.; Bruhn, C.; Pietschnig, R.; Kelemen, Z. The "Chemical Tug-of-War" in Carborane Clusters: Distinct Tuning on Different Sides of the Cluster. *Inorg. Chem. Front.* **2025**, *12* (5), 1822–1830. https://doi.org/10.1039/D4QI02566K.

(7) Monaco, G.; Summa, F. F.; Zanasi, R. Program Package for the Calculation of Origin-Independent Electron Current Density and Derived Magnetic Properties in Molecular Systems. *J. Chem. Inf. Model.* **2021**, *61* (1), 270–283. https://doi.org/10.1021/acs.jcim.0c01136.

(8) Glendening, E. D.; Landis, C. R.; Weinhold, F. NBO 6.0: Natural Bond Orbital Analysis Program. *J. Comput. Chem.* **2013**, *34* (16), 1429–1437. https://doi.org/10.1002/jcc.23266.

(9) Lu, T.; Chen, F. Multiwfn: A Multifunctional Wavefunction Analyzer. *J. Comput. Chem.* **2012**, *33* (5), 580–592. https://doi.org/10.1002/jcc.22885.

(10) Szczepanik, D. W.; Andrzejak, M.; Dominikowska, J.; Pawełek, B.; Krygowski, T. M.; Szatylowicz, H.; Solà, M. The Electron Density of Delocalized Bonds (EDDB) Applied for Quantifying Aromaticity. *Phys. Chem. Chem. Phys.* **2017**, *19* (42), 28970–28981. https://doi.org/10.1039/C7CP06114E.

(11) Matito, E. An Electronic Aromaticity Index for Large Rings. *Phys. Chem. Chem. Phys.* **2016**, *18* (17), 11839–11846. https://doi.org/10.1039/C6CP00636A.

(12) Szczepanik, D. W.; Solà, M. 8 - The Electron Density of Delocalized Bonds (EDDBs) as a Measure of Local and Global Aromaticity. In *Aromaticity*; Fernandez, I., Ed.; Elsevier, 2021; pp 259–284. https://doi.org/10.1016/B978-0-12-822723-7.00008-X.

(13) Hanwell, M. D.; Curtis, D. E.; Lonie, D. C.; Vandermeersch, T.; Zurek, E.; Hutchison, G. R. Avogadro: An Advanced Semantic Chemical Editor, Visualization, and Analysis Platform. *J. Cheminformatics* **2012**, *4* (1), 17. https://doi.org/10.1186/1758-2946-4-17.

(14) Keith, T. A.; Bader, R. F. W. Calculation of Magnetic Response Properties Using Atoms in Molecules. *Chem. Phys. Lett.* **1992**, *194* (1), 1–8. https://doi.org/10.1016/0009-2614(92)85733-Q.

XYZ Geometries and Total Energies of the Investigated Systems

6
ethylene, E(B3LYP/6-311+G**)=-78.615512

C	0.003679	-0.000000	0.004592
C	0.002016	-0.000000	1.333638
H	0.927064	-0.000000	-0.564437
H	0.924026	-0.000000	1.904741
H	-0.921197	0.000000	1.902976
H	-0.919134	0.000000	-0.567011

12
benzene, E(B3LYP/6-311+G**)=-232.311238

C	0.022373	-0.000000	-0.021889
C	0.008194	-0.000000	1.372133
C	1.209529	0.000000	2.081714
C	2.424050	0.000000	1.397018
C	2.438125	0.000000	0.001995
C	1.237755	-0.000000	-0.707027
H	-0.935755	-0.000000	1.905893
H	1.198200	0.000000	3.166073
H	3.357521	0.000000	1.948869
H	3.382725	0.000000	-0.530576
H	1.247583	-0.000000	-1.791381
H	-0.911257	-0.000000	-0.573640

11
phosphabenzene, E(B3LYP/6-311+G**)=-534.955282

P	-0.344574	0.000000	-0.198940
C	-0.042428	0.000000	1.518276
C	1.213729	-0.000000	2.114815
C	2.405626	-0.000000	1.388889
C	2.438348	-0.000000	-0.006288
C	1.293651	0.000000	-0.795882
H	-0.915834	0.000000	2.164266
H	1.274298	-0.000000	3.199721
H	3.344931	-0.000000	1.931197
H	3.408189	-0.000000	-0.496286
H	1.416392	0.000000	-1.875268

17
phosphanaphthalene, E(B3LYP/6-311+G**)=-688.632826

C	-0.003162	-0.000000	-0.090318
C	-0.052163	-0.000000	1.284936
C	1.132229	-0.000000	2.067181
C	2.395957	0.000000	1.395563
C	2.410618	0.000000	-0.027000
C	1.243962	-0.000000	-0.752642
P	0.951439	-0.000000	3.841269
C	2.595270	0.000000	4.338929

C	3.716351	0.000000	3.485450
C	3.623326	0.000000	2.113194
H	4.538770	0.000000	1.529856
H	2.773888	0.000000	5.410377
H	4.705821	0.000000	3.933492
H	3.369547	0.000000	-0.535045
H	1.277935	0.000000	-1.836349
H	-1.011948	-0.000000	1.791947
H	-0.920719	-0.000000	-0.667745

24
***ortho*-carborane**, E(B3LYP/6-311+G**)=-332.163845

C	0.000162	0.000689	0.000273
B	0.000197	-0.000056	1.720689
B	1.729965	-0.000015	2.137471
B	2.677226	-0.063444	0.625633
B	2.454045	1.482655	1.474514
B	0.793622	1.515141	2.150314
B	-0.341453	1.469659	0.778681
B	0.605252	1.405296	-0.733581
B	1.546290	-0.104746	-0.747065
C	1.168649	-0.828394	0.767423
B	2.335971	1.411178	-0.312632
B	1.171269	2.392339	0.632996
H	1.075817	-1.904043	0.754736
H	0.114551	1.757093	-1.746737
H	3.639017	-0.743511	0.566142
H	3.361239	2.008260	2.020008
H	1.155474	3.572572	0.572719
H	2.071419	-0.637455	3.069214
H	1.654087	-0.814184	-1.680489
H	3.143376	1.881633	-1.035514
H	-1.453257	1.862665	0.756142
H	0.515860	2.058785	3.161762
H	-0.816677	-0.648127	2.267450
H	-0.777360	-0.589574	-0.461108

10
$\mathbf{1}_{\mathrm{H}}$, E(B3LYP/6-311+G**)=-496.812662

P	-0.413971	0.119133	-0.277476
C	-0.054958	-0.048374	1.493607
C	1.281711	-0.000128	1.702667
C	2.079747	0.001200	0.482875
C	1.352856	-0.045963	-0.658235
H	1.762739	-0.037393	-1.659093
H	3.163584	0.038332	0.506162
H	1.737133	0.035984	2.686493
H	-0.807858	-0.041972	2.270069
H	-0.882648	-1.188411	-0.585551

22
1$_{t\text{bu}}$, E(B3LYP/6-311+G**)= -654.114181

P	-0.227297	0.093197	-0.192015
C	0.097751	0.009223	1.584526
C	1.428071	0.138086	1.817616
C	2.248398	0.132171	0.614359
C	1.544748	-0.001204	-0.537934
H	1.979305	0.002605	-1.528558
H	3.326862	0.243565	0.654604
H	1.860115	0.254133	2.806041
H	-0.665566	0.021685	2.350934
C	-1.007817	-1.569004	-0.715966
C	-1.031460	-1.589182	-2.255052
C	-0.233164	-2.782406	-0.181839
C	-2.449214	-1.578923	-0.175568
H	-0.722383	-3.707401	-0.510849
H	0.794880	-2.795317	-0.549553
H	-0.199944	-2.788150	0.909545
H	-2.959591	-2.490374	-0.506313
H	-2.471533	-1.567219	0.917494
H	-3.023868	-0.722427	-0.538339
H	-1.530068	-2.500677	-2.603167
H	-1.575097	-0.732867	-2.663217
H	-0.022076	-1.585029	-2.675229

20
1$_{\text{Ph}}$, E(B3LYP/6-311+G**)=-727.922686

C	0.376262	0.509585	-0.104430
C	-0.131146	0.261331	1.126350
C	0.821041	-0.338774	2.056161
C	2.052404	-0.546690	1.532313
P	2.164284	0.211968	-0.110043
H	2.895888	-0.966274	2.063643
H	0.549191	-0.581852	3.077930
H	-1.150901	0.489717	1.417798
H	-0.164215	0.962640	-0.924463
C	2.458518	-1.171715	-1.304105
C	3.475892	-1.026471	-2.252840
C	3.735311	-2.042747	-3.174191
C	2.979104	-3.210951	-3.152884
C	1.961441	-3.363488	-2.208597
C	1.701880	-2.351467	-1.289860
H	4.066574	-0.117085	-2.271440
H	4.526701	-1.918571	-3.904954
H	3.179036	-4.001468	-3.867622
H	1.370561	-4.272726	-2.189656
H	0.911184	-2.473019	-0.558133

29
1$_{\text{Mes}}$, E(B3LYP/6-311+G**)=-845.898773

C	-0.044284	-0.017963	0.009132
C	-0.029323	-0.016621	1.405948
C	1.216901	0.001107	2.077020
C	2.415267	0.019228	1.325607
C	2.343546	0.016988	-0.070304
C	1.126535	-0.004657	-0.748684
C	-1.346818	-0.031171	2.137538
P	1.390894	0.006247	3.919533
C	0.385745	1.282222	4.692271
C	-0.390938	0.716212	5.655521
C	-0.376040	-0.734795	5.657652
C	0.412029	-1.287583	4.695981
C	3.787659	0.045002	1.966484
C	1.075031	-0.039473	-2.256330
H	0.577261	-2.347189	4.555815
H	-0.934674	-1.314526	6.384466
H	-0.961462	1.286493	6.380576
H	0.529201	2.344563	4.548902
H	3.267361	0.034678	-0.640378
H	-1.003753	-0.027733	-0.499527
H	-1.436434	-0.911341	2.778694
H	-1.451562	0.840699	2.787565
H	-2.178643	-0.035108	1.431044
H	4.562312	0.054747	1.197290
H	3.927357	0.927059	2.596490
H	3.956535	-0.826563	2.603825
H	0.207110	0.503431	-2.638311
H	1.973164	0.399194	-2.696691
H	1.000846	-1.070555	-2.619282

56

1$_{Mes*}$, E(B3LYP/6-311+G**)=-1199.764873

C	2.912020	-0.439557	1.640953
C	4.247789	-0.571406	1.376501
C	4.662952	-0.010841	0.112757
C	3.655380	0.559862	-0.613202
P	2.208254	0.651177	0.425944
H	3.773562	1.057185	-1.564888
H	5.697967	-0.020478	-0.209873
H	4.952486	-1.019450	2.067777
H	2.421488	-0.741705	2.555217
C	0.496160	0.212047	-0.147222
C	-0.438828	1.305280	-0.154686
C	-1.794754	1.011264	-0.032122
C	-2.289335	-0.287527	0.103817
C	-1.376515	-1.319214	-0.041911
C	0.002572	-1.121756	-0.230420
C	-0.060901	2.813809	-0.321136
H	-2.502327	1.824822	-0.016952
C	-3.789823	-0.519075	0.352758

H	-1.740168	-2.333827	-0.015980
C	0.788549	-2.430192	-0.560818
C	2.169954	-2.212973	-1.200140
C	0.947928	-3.295034	0.710336
C	-0.017852	-3.246304	-1.612396
C	-1.273209	3.645912	-0.812220
C	0.384227	3.441354	1.022981
C	1.027360	3.009065	-1.403815
C	-4.139224	-2.011835	0.495241
C	-4.205231	0.197427	1.659294
C	-4.606311	0.055928	-0.828467
H	1.436542	-4.242436	0.460809
H	1.561593	-2.787713	1.456203
H	-0.020275	-3.525806	1.162641
H	0.573689	-4.113828	-1.916886
H	-0.969502	-3.625320	-1.238790
H	-0.218955	-2.646393	-2.504235
H	2.538659	-3.173438	-1.572582
H	2.115894	-1.528729	-2.050067
H	2.908179	-1.840970	-0.499476
H	-0.931019	4.658778	-1.038556
H	-1.714686	3.231106	-1.722344
H	-2.056831	3.741190	-0.057505
H	0.579249	4.510106	0.885728
H	-0.405995	3.337194	1.772065
H	1.289517	2.979731	1.414769
H	1.166209	4.077367	-1.594271
H	1.998087	2.615705	-1.110230
H	0.730571	2.534849	-2.343557
H	-5.271718	0.045284	1.852202
H	-3.646133	-0.194011	2.513689
H	-4.025956	1.273682	1.607822
H	-5.208884	-2.119523	0.694675
H	-3.918572	-2.572468	-0.417209

12

3$_H$, E(B3LYP/6-311+G**)=-498.0338099

C	-0.07685	-1.30181	0.14475
C	-1.42581	-0.57673	-0.09990
C	-1.19165	0.90724	0.04543
C	0.07720	1.32085	0.07374
P	1.29712	-0.03275	-0.16381
H	0.36684	2.36467	0.11846
H	-2.03584	1.59086	0.07811
H	-2.20178	-0.93050	0.58761
H	0.04651	-2.16229	-0.51333
H	1.88227	-0.02196	1.13826
H	-0.01360	-1.65753	1.17411
H	-1.79854	-0.78939	-1.11016

24

3$_{t\text{bu}}$, E(B3LYP/6-311+G**)=-654.1141811

C	1.31663	1.29537	-0.37283
P	0.13789	-0.02172	-1.04455
C	1.21256	-1.33069	-0.33567
C	2.25948	-0.87407	0.35746
C	2.37312	0.61874	0.53640
H	1.04138	-2.38561	-0.51907
H	3.00682	-1.52987	0.79818
H	2.22474	0.87358	1.59368
H	1.79819	1.75407	-1.23865
C	-1.35495	0.00684	0.16013
H	0.76757	2.08880	0.13764
H	3.38149	0.96518	0.28510
C	-2.14091	-1.29529	-0.08245
C	-0.97469	0.11285	1.64369
C	-2.22588	1.21002	-0.24560
H	-3.13485	1.24027	0.36625
H	-1.70260	2.16023	-0.10072
H	-2.52809	1.15016	-1.29490
H	-1.87801	0.08079	2.26509
H	-0.32733	-0.71044	1.95454
H	-0.46266	1.05262	1.86709
H	-3.06992	-1.28654	0.49911
H	-2.40827	-1.41440	-1.13651
H	-1.56904	-2.17562	0.22455

22

3$_{\text{Ph}}$, E(B3LYP/6-311+G**)=-729.145659

C	-0.020447	-0.941119	0.729178
C	0.389837	-0.231379	2.043021
C	1.649625	0.552388	1.766075
C	1.975818	0.733912	0.482936
P	0.749718	0.047332	-0.693323
H	2.821317	1.329093	0.156298
H	2.215862	0.983831	2.587503
H	0.526948	-0.943417	2.864177
H	-1.103220	-0.998954	0.613140
C	1.723282	-1.244737	-1.607992
H	0.375455	-1.958435	0.699803
H	-0.400734	0.456929	2.368821
C	2.792826	-1.953579	-1.043867
C	3.476792	-2.915047	-1.783980
C	3.099737	-3.185852	-3.100046
C	2.040203	-2.486625	-3.673286
C	1.361902	-1.518081	-2.932908
H	3.095424	-1.747429	-0.022638
H	4.305462	-3.452607	-1.335540
H	3.632876	-3.934691	-3.675276
H	1.744884	-2.688905	-4.697020

H	0.546035	-0.965560	-3.387646

31

$\mathbf{3}_{Mes}$, E(B3LYP/6-311+G**)=-845.8987732

C	-0.68814	-1.08860	-0.03898
C	-0.29695	0.27170	-0.11628
C	-1.30221	1.27116	-0.08210
C	-2.64584	0.89982	0.02415
C	-3.04253	-0.43307	0.09138
C	-2.04572	-1.40667	0.06170
P	1.45158	0.91427	-0.26408
C	2.43476	-0.27603	-1.24513
C	3.52272	-0.71118	-0.60307
C	3.68315	-0.28021	0.83623
C	2.34017	0.33656	1.30412
C	-0.99377	2.75489	-0.14533
C	-4.50081	-0.81305	0.16888
C	0.28517	-2.24643	-0.06638
H	2.24360	-0.46506	-2.29571
H	4.29688	-1.30420	-1.08271
H	3.99320	-1.11409	1.47626
H	2.49304	1.17880	1.97975
H	1.72840	-0.39930	1.82892
H	4.48621	0.46527	0.90358
H	-3.40149	1.67913	0.05521
H	-2.32958	-2.45344	0.12314
H	-1.92153	3.33053	-0.12423
H	-0.37860	3.08293	0.69675
H	-0.44641	3.01985	-1.05233
H	0.77608	-2.33966	-1.03736
H	1.07910	-2.14501	0.67441
H	-0.24136	-3.18174	0.13370
H	-4.64490	-1.72546	0.75279
H	-5.09637	-0.01868	0.62459
H	-4.90987	-0.99722	-0.83076

58

$\mathbf{3}_{Mes*}$, E(B3LYP/6-311+G**)=-1200.9786532

C	-3.66375	0.72305	-0.43894
P	-2.10538	0.79799	0.64148
C	-2.72792	-0.56566	1.67212
C	-4.02698	-0.82056	1.48665
C	-4.72009	-0.08985	0.36060
H	-2.14424	-0.98704	2.48219
H	-4.59802	-1.48527	2.12946
H	-5.27332	-0.78564	-0.28132
H	-4.00583	1.74391	-0.61167
C	-0.47869	0.25269	-0.15508
H	-3.45879	0.28058	-1.41125
H	-5.47212	0.58893	0.78106

C	0.50104	1.30652	-0.18026
C	1.84579	0.97619	-0.02601
C	2.29793	-0.33478	0.13758
C	1.35751	-1.33610	-0.04753
C	-0.00906	-1.09054	-0.26845
C	0.18643	2.82335	-0.39712
H	2.57512	1.77185	-0.00450
C	3.78036	-0.61213	0.44025
H	1.68858	-2.36337	-0.03367
C	-0.80480	-2.35621	-0.71901
C	1.35798	3.51521	-1.14450
C	-1.05031	3.04727	-1.29705
C	0.00398	3.55863	0.95225
C	-2.17925	-2.06005	-1.33866
C	0.00121	-3.05455	-1.85320
C	-0.97479	-3.36162	0.44238
C	4.07372	-2.11381	0.61277
C	4.65730	-0.08015	-0.71749
C	4.17485	0.10659	1.75196
H	-0.58373	-3.89137	-2.24596
H	0.19615	-2.36221	-2.67674
H	0.95783	-3.45760	-1.52009
H	-2.57304	-2.97690	-1.78745
H	-2.90040	-1.72910	-0.60135
H	-2.09942	-1.31180	-2.13104
H	-1.46018	-4.27443	0.08148
H	-0.00994	-3.64468	0.87080
H	-1.59214	-2.94770	1.24015
H	5.12995	-2.25500	0.85822
H	3.48444	-2.55229	1.42285
H	3.87266	-2.67758	-0.30231
H	5.22914	-0.07583	1.98289
H	4.03044	1.18705	1.68208
H	3.57505	-0.25661	2.59110
H	5.71527	-0.27079	-0.51099
H	4.40056	-0.57312	-1.65927
H	4.53449	0.99596	-0.85944
H	1.05318	4.52941	-1.41592
H	2.25738	3.61265	-0.53404
H	1.62167	2.98467	-2.06346
H	-0.16112	4.62733	0.77712
H	-0.84470	3.16445	1.51036
H	0.90042	3.45436	1.57017
H	-1.11892	4.10591	-1.56444
H	-0.97534	2.47021	-2.22290
H	-1.98519	2.79032	-0.80374

28

4$_H$, E(B3LYP/6-311+G**)=-750.340282

C	0.011470	0.001856	-0.010757

P	0.018767	-0.013363	1.867196
C	1.864202	0.009220	1.862172
C	2.460794	-0.081500	0.670690
C	1.569165	-0.097579	-0.525093
B	0.720723	1.398424	-0.772542
B	-0.946305	0.893937	-1.104220
B	0.170582	1.335532	-2.429805
B	1.792740	0.714747	-2.008465
B	1.700673	-1.065660	-1.920588
B	0.776104	-0.182252	-3.158311
B	-0.921062	-0.068784	-2.604404
B	0.021487	-1.555008	-2.299286
B	0.563723	-1.516172	-0.635011
B	-1.039478	-0.884134	-1.029793
H	0.038387	2.342533	-3.033617
H	-0.211269	-2.594858	-2.809506
H	-1.838980	-0.053793	-3.348402
H	1.071486	-0.250370	-4.300474
H	0.772781	-2.370496	0.147091
H	2.676683	-1.714870	-2.054472
H	-1.974105	-1.405618	-0.534110
H	-1.822522	1.553169	-0.668357
H	1.015168	2.299331	-0.076189
H	2.830853	1.242787	-2.197906
H	3.532821	-0.142458	0.525298
H	2.417049	0.019747	2.794246
H	-0.180928	1.383913	2.056742

40

4$_{t\mathrm{bu}}$, E(B3LYP/6-311+G**)=-907.635298

C	-0.200604	-0.066453	-0.284377
P	-0.460310	-0.160725	1.589894
C	1.327289	0.153942	1.847054
C	2.112983	0.094716	0.765051
C	1.426484	-0.058984	-0.549568
B	0.528647	1.342187	-1.016942
B	-1.022963	0.693402	-1.576493
B	0.256372	1.161718	-2.734254
B	1.829455	0.695860	-2.029711
B	1.856686	-1.075944	-1.853591
B	1.081387	-0.329991	-3.271695
B	-0.686632	-0.331204	-2.996324
B	0.302382	-1.718639	-2.464046
B	0.565932	-1.561235	-0.743671
B	-0.995446	-1.080234	-1.414900
H	0.148903	2.128969	-3.404947
H	0.229385	-2.796330	-2.943253
H	-1.473068	-0.426465	-3.873568
H	1.561732	-0.423050	-4.347625
H	0.707974	-2.364850	0.103488

H	2.885695	-1.650048	-1.791523
H	-1.957443	-1.657123	-1.049141
H	-2.007049	1.300556	-1.344954
H	0.674572	2.303768	-0.364977
H	2.840770	1.302010	-2.082522
H	3.194962	0.147473	0.787438
H	1.735275	0.245906	2.846940
C	-1.381900	1.368245	2.275235
C	-1.212337	1.225775	3.804460
C	-0.900811	2.755501	1.835145
C	-2.867310	1.177794	1.911633
H	-1.434538	3.517585	2.414935
H	0.168759	2.894166	2.008426
H	-1.105091	2.944813	0.781725
H	-3.463377	1.958041	2.396925
H	-3.035900	1.253995	0.835214
H	-3.245342	0.208951	2.248789
H	-1.843296	1.968054	4.303981
H	-1.515472	0.237874	4.162707
H	-0.181602	1.406535	4.121471

38

4$_{Ph}$, E(B3LYP/6-311+G**)=-981.449823

C	0.116033	0.131474	-0.111461
P	0.242732	0.267974	1.773154
C	2.074137	0.191649	1.649111
C	2.589621	-0.063229	0.442010
C	1.626343	-0.145417	-0.694129
B	0.867710	1.368285	-1.073811
B	-0.846218	0.956388	-1.254358
B	0.214460	1.154940	-2.681045
B	1.813871	0.468412	-2.275438
B	1.607085	-1.276688	-1.969789
B	0.669733	-0.476985	-3.253398
B	-0.978074	-0.173953	-2.627275
B	-0.121600	-1.680495	-2.196173
B	0.523182	-1.488173	-0.581828
B	-1.053170	-0.787467	-0.961492
H	0.115754	2.092085	-3.394183
H	-0.456131	-2.752372	-2.564330
H	-1.937562	-0.178553	-3.317462
H	0.889163	-0.698902	-4.393382
H	0.720762	-2.260211	0.283993
H	2.526284	-2.009114	-2.074849
H	-1.988841	-1.178569	-0.358556
H	-1.647923	1.726090	-0.860561
H	1.272293	2.324724	-0.527220
H	2.870971	0.893307	-2.583698
H	3.643629	-0.221813	0.246082
H	2.685865	0.251581	2.542021

C	-0.105932	2.036709	2.168875
C	-1.417633	2.336892	2.563394
C	-1.769962	3.636028	2.920667
C	-0.808042	4.644451	2.916804
C	0.504182	4.351467	2.548189
C	0.854042	3.057171	2.170595
H	-2.164894	1.550708	2.593778
H	-2.790376	3.856721	3.212469
H	-1.078166	5.653911	3.205474
H	1.256117	5.132639	2.548846
H	1.874018	2.844256	1.874264

47

4$_{Mes}$, E(B3LYP/6-311+G**)=-1099.420592

C	-0.024752	-0.021577	-0.143347
P	0.023798	0.179413	1.744982
C	1.848888	0.332088	1.677676
C	2.433376	0.078230	0.502047
C	1.529179	-0.124434	-0.664249
B	0.610850	1.283975	-1.099275
B	-1.028512	0.665848	-1.343182
B	0.058114	0.974486	-2.729015
B	1.708073	0.491803	-2.247128
B	1.699696	-1.262192	-1.920788
B	0.729796	-0.596372	-3.255172
B	-0.967407	-0.487754	-2.700377
B	0.044977	-1.874458	-2.211077
B	0.593119	-1.587750	-0.574091
B	-1.036370	-1.087536	-1.029596
H	-0.124091	1.884848	-3.460334
H	-0.143494	-2.982993	-2.574756
H	-1.891066	-0.615303	-3.426983
H	1.022145	-0.804326	-4.381431
H	0.845124	-2.322078	0.310018
H	2.704528	-1.878565	-1.976131
H	-1.943915	-1.587514	-0.466045
H	-1.932725	1.340358	-1.001102
H	0.866766	2.290338	-0.551357
H	2.719153	1.037100	-2.518355
H	3.503699	-0.003629	0.354982
H	2.420539	0.458994	2.588545
C	-0.760965	1.827918	2.098679
C	-2.173919	1.764400	2.224496
C	-2.889703	2.923512	2.528557
C	-2.261652	4.148539	2.736797
C	-0.871601	4.184458	2.650397
C	-0.102533	3.058756	2.340753
C	-2.960611	0.484637	2.037790
H	-3.970123	2.858991	2.612559
C	-3.057811	5.394986	3.031077

H	-0.358423	5.123661	2.832456
C	1.398094	3.248858	2.329776
H	1.859808	2.888464	1.411667
H	1.638264	4.308366	2.431784
H	1.874980	2.728972	3.165858
H	-3.995964	0.631466	2.349641
H	-2.977886	0.163018	0.993207
H	-2.544691	-0.339779	2.622568
H	-2.484524	6.102255	3.634556
H	-3.335197	5.905484	2.102299
H	-3.982348	5.161139	3.563811

74

4$_{Mes*}$, E(B3LYP/6-311+G**)=-1452.662289

C	2.058998	0.429802	0.021199
P	1.107715	0.234341	-1.657770
C	2.287719	-1.152772	-2.120424
C	3.354663	-1.331942	-1.322168
C	3.395875	-0.558443	-0.051651
B	2.117253	-0.966359	1.084982
B	1.514332	0.611890	1.648149
B	2.672531	-0.353873	2.634736
B	3.846094	-1.126589	1.513860
B	4.766265	0.177752	0.681312
B	4.312480	0.349557	2.404797
B	2.871315	1.426253	2.494552
B	4.170040	1.759734	1.297536
B	3.630558	1.178547	-0.271031
B	2.441389	1.914257	0.829127
H	2.331776	-0.892867	3.631898
H	4.879855	2.704758	1.356953
H	2.668979	2.149708	3.409275
H	5.132012	0.312423	3.257907
H	3.852775	1.548067	-1.366243
H	5.832406	-0.048173	0.225414
H	1.901262	2.904524	0.488558
H	0.359761	0.736543	1.851601
H	1.442083	-1.883664	0.809471
H	4.305987	-2.211271	1.603001
H	4.190096	-1.980382	-1.563400
H	2.187926	-1.631063	-3.085975
C	-0.690463	-0.169990	-1.063432
C	-1.439970	1.060863	-0.943958
C	-2.414053	1.128492	0.062284
C	-2.750183	0.041150	0.881295
C	-2.203999	-1.192684	0.532907
C	-1.218083	-1.354319	-0.464011
C	-1.353424	2.281886	-1.935533
H	-2.942033	2.058844	0.210491
C	-3.752401	0.223576	2.039120

H	-2.565978	-2.080857	1.031007
C	-1.048183	-2.838494	-0.936827
C	-0.023289	-3.072026	-2.059326
C	-0.702823	-3.804665	0.228330
C	-2.437680	-3.249010	-1.527936
C	-2.684664	3.093241	-1.904281
C	-0.227196	3.296395	-1.594698
C	-1.214663	1.784060	-3.402861
C	-3.963420	-1.080827	2.841649
C	-3.212035	1.308158	3.011837
C	-5.127910	0.677469	1.476109
H	-0.329339	4.182401	-2.234019
H	-0.303952	3.621700	-0.551229
H	0.768806	2.882567	-1.753443
H	-2.664876	3.822244	-2.721551
H	-3.559218	2.449238	-2.045047
H	-2.810973	3.659439	-0.975604
H	-1.251776	2.644153	-4.082051
H	-0.273807	1.263139	-3.584584
H	-2.041138	1.111403	-3.659945
H	-2.389920	-4.283110	-1.890365
H	-3.237318	-3.185202	-0.785037
H	-2.704941	-2.600739	-2.370225
H	-0.166798	-4.079038	-2.468451
H	-0.152468	-2.359750	-2.881647
H	0.999689	-3.011899	-1.690270
H	-0.659921	-4.832462	-0.151938
H	0.269780	-3.566363	0.667754
H	-1.449225	-3.782421	1.027709
H	-4.642442	-0.888680	3.679862
H	-4.413596	-1.869767	2.227775
H	-3.020255	-1.457138	3.253740
H	-5.843837	0.813899	2.295714
H	-5.051549	1.626857	0.935578
H	-5.533975	-0.072721	0.787676
H	-3.917255	1.457596	3.838393
H	-2.246663	1.006927	3.433629
H	-3.073848	2.270673	2.508316

46

5$_H$, E(B3LYP/6-311+G**)=-1003.842742

C	0.000519	-0.001637	-0.001927
B	-0.002242	-0.001380	1.723819
B	1.716288	0.000417	2.191010
B	1.155433	0.988303	0.825771
B	1.599147	0.142658	-0.648529
B	2.372494	-1.402870	-0.252386
B	1.213063	-2.405014	0.664371
B	2.468846	-1.481229	1.525758
B	2.690175	0.080451	0.704420

P	-1.358132	0.654035	-1.154758
C	-1.186621	-0.789026	-2.376648
B	-1.628572	-2.380372	-1.826365
B	-2.457086	-1.629767	-3.187437
B	-1.713851	-2.274717	-4.671761
B	-0.387474	-3.375705	-4.189384
B	0.839663	-2.419371	-3.319480
B	0.264760	-0.742151	-3.317781
B	-0.024509	-1.719827	-4.726753
B	-1.305925	-0.644094	-4.098151
C	0.691181	-1.370778	-0.608216
B	-0.295959	-1.496320	0.839949
B	0.811638	-1.531706	2.176231
C	0.029898	-1.809413	-1.931379
B	-0.323324	-3.424107	-2.410070
B	-1.901628	-3.331580	-3.252668
H	-2.440565	0.094520	-0.428077
H	3.364226	-2.041889	2.053453
H	-0.923133	0.492898	2.270570
H	1.747972	0.715913	-1.650414
H	1.016055	2.156621	0.752778
H	3.094633	-1.893789	-1.041170
H	3.725593	0.640656	0.614975
H	-1.360382	-1.964279	0.793035
H	0.496337	-2.124344	3.147624
H	2.065046	0.509890	3.197673
H	1.156753	-3.567059	0.486998
H	-2.670581	-4.225415	-3.189368
H	0.559575	-1.458750	-5.719026
H	-0.072347	-4.321220	-4.822789
H	-2.357484	-2.423508	-5.650620
H	0.955414	0.190958	-3.234957
H	-1.625227	0.404941	-4.530924
H	0.054780	-4.298163	-1.718661
H	-2.153228	-2.490625	-0.793523
H	-3.562047	-1.257712	-3.008759
H	1.996012	-2.622189	-3.239564

58

5$_{t\mathrm{bu}}$, E(B3LYP/6-311+G**)=-1161.132206

C	1.392816	0.291824	-0.300043
B	2.878704	0.570090	0.547489
B	4.003952	-0.676362	-0.037492
B	2.887308	0.125979	-1.163397
B	1.567122	-0.991171	-1.469423
B	1.778493	-2.458424	-0.503840
B	1.772538	-2.010659	1.221479
B	3.315853	-2.275377	0.376167
B	3.169066	-1.637731	-1.277325
P	-0.000233	1.467152	-0.903876

C	-1.392656	0.291066	-0.300554
B	-1.547593	-0.257189	1.346292
B	-2.878620	0.569147	0.546943
B	-4.003536	-0.677771	-0.037553
B	-3.314905	-2.276433	0.376721
B	-1.777419	-2.459128	-0.503376
B	-1.566771	-0.992289	-1.469769
B	-3.168426	-1.639342	-1.277012
B	-2.887130	0.124480	-1.163779
C	0.770878	-1.190387	0.085535
B	1.547799	-0.257095	1.346304
B	3.152331	-0.911342	1.507115
C	-0.770445	-1.190513	0.085487
B	-1.771604	-2.010787	1.221854
B	-3.151903	-0.911957	1.507187
C	-0.000810	3.076946	0.121681
H	3.979490	-3.219315	0.628995
H	3.133635	1.664537	0.898203
H	0.938733	-0.930731	-2.447792
H	3.120215	0.928807	-1.995331
H	1.260269	-3.450689	-0.866584
H	3.698991	-2.110337	-2.221045
H	0.920761	0.248345	2.183520
H	3.672686	-0.859429	2.566304
H	5.164323	-0.459914	-0.086088
H	1.250941	-2.701580	2.018485
H	-3.672375	-0.859762	2.566303
H	-3.698293	-2.112564	-2.220459
H	-3.978288	-3.220485	0.629770
H	-5.163948	-0.461578	-0.086260
H	-0.938228	-0.932164	-2.448051
H	-3.120455	0.927111	-1.995791
H	-1.249636	-2.701550	2.018754
H	-0.920347	0.249054	2.182833
H	-3.134010	1.663710	0.896948
H	-1.258817	-3.451405	-0.865544
C	-1.251941	3.836379	-0.376212
C	-0.001433	3.004206	1.652172
C	1.250370	3.836694	-0.375200
H	-0.001032	4.025156	2.050096
H	-0.887712	2.503745	2.041806
H	0.884057	2.502827	2.042465
H	-1.225992	4.853668	0.027295
H	-1.274902	3.913761	-1.466710
H	-2.180979	3.372163	-0.044163
H	1.223407	4.854306	0.027428
H	2.179268	3.373272	-0.041628
H	1.274761	3.913284	-1.465732

56
5$_{Ph}$, E(B3LYP/6-311+G**)=-1234.946156

C	-2.394934	-1.003687	-0.350890
P	-0.878763	-0.455703	-1.239019
C	0.652875	-1.287716	-0.454921
C	1.683479	-0.155502	0.170710
B	2.696546	-0.835023	1.383990
B	2.224413	-2.560467	1.469057
B	3.668343	-2.068088	0.549114
B	3.240496	-2.052965	-1.174785
B	1.518929	-2.515032	-1.316989
B	2.024877	-0.835894	-1.420676
B	3.316190	-0.516221	-0.255405
C	-0.453280	1.254051	-0.488885
B	-0.388164	1.425512	1.229039
B	-0.520866	3.125883	1.565892
B	0.851243	3.954586	0.793559
B	0.696921	3.749785	-0.967996
B	1.848007	2.737036	-0.040512
C	1.037521	1.244592	0.212049
B	1.093289	2.351644	1.530521
B	-1.512409	2.383633	0.274644
B	-0.762850	2.763770	-1.280504
B	0.844567	2.055876	-1.332242
B	-0.762954	3.976184	0.020410
B	1.005788	-1.340940	1.255255
B	0.887383	-2.822676	0.305475
B	2.553874	-3.303599	-0.111283
H	1.351731	4.912947	1.269494
H	-2.654621	2.102763	0.343201
H	1.270131	1.574526	-2.303256
H	-1.395588	2.737969	-2.275418
H	3.018415	2.714322	-0.162966
H	1.089296	4.530161	-1.762957
H	-0.764229	0.548515	1.893661
H	-1.004565	3.459177	2.590688
H	-1.430537	4.947469	-0.060729
H	1.757709	2.071885	2.461068
H	2.258067	-3.142351	2.496465
H	4.002826	-2.268418	-2.050630
H	4.760251	-2.317496	0.924359
H	2.840531	-4.444429	-0.219214
H	1.824572	-0.211773	-2.383266
H	0.990047	-2.975781	-2.264797
H	3.006517	-0.148350	2.288111
H	0.209530	-1.023455	2.039766
H	-0.054256	-3.511965	0.468842
H	4.047263	0.384233	-0.454968
C	-3.546368	-0.888154	-1.147550
C	-4.795387	-1.253137	-0.651661

C	-4.906377	-1.767086	0.637568
C	-3.766446	-1.910506	1.427578
C	-2.518950	-1.528025	0.942503
H	-3.463358	-0.513838	-2.162643
H	-5.674359	-1.147779	-1.276881
H	-5.875063	-2.063228	1.023645
H	-3.846909	-2.320871	2.427693
H	-1.652875	-1.652687	1.574202

65

5$_{Mes}$, E(B3LYP/6-311+G**)=-1352.905034

C	4.015944	-1.178740	-1.242328
C	2.645715	-1.122345	-0.970129
C	2.212496	-0.614642	0.279999
C	3.197848	-0.218294	1.236700
C	4.548983	-0.283245	0.894807
C	4.985412	-0.751189	-0.341285
C	1.741839	-1.635352	-2.062200
P	0.521264	-0.362982	1.018850
C	-0.121591	1.298283	0.305278
C	-1.673795	1.152000	-0.225422
B	-1.971206	2.225010	-1.544866
B	-0.393372	1.434362	-1.394083
B	0.740747	2.503252	-0.582522
B	-0.113663	4.029587	-0.264066
B	-0.445700	3.132481	-1.765108
B	-1.793300	3.856122	-0.857643
B	-2.584083	2.574878	0.093104
B	-1.396007	2.006714	1.276687
B	-1.434798	3.699404	0.878773
B	0.129219	2.847451	1.041522
C	2.886173	0.280621	2.637942
C	6.450998	-0.787021	-0.690362
C	-0.990784	-1.351283	0.334337
B	-2.236922	-0.996015	1.507669
B	-3.712284	-0.767102	0.564354
C	-2.191714	-0.294434	-0.104999
B	-1.611877	-1.421545	-1.298098
B	-3.334859	-1.039170	-1.153862
B	-4.079527	-2.337376	-0.194241
B	-2.794294	-3.490894	0.272003
B	-1.639420	-2.633484	1.310372
B	-3.390744	-2.292969	1.442246
B	-1.252205	-2.896614	-0.392303
B	-2.765218	-2.724850	-1.332937
H	-2.424128	4.758739	-1.285434
H	1.889788	2.325329	-0.771515
H	-1.678893	1.510023	2.291606
H	0.862229	2.912730	1.962268
H	-3.728147	2.452935	0.340853

H	-1.803308	4.460277	1.703709
H	-0.030644	0.572688	-2.082033
H	-0.105404	3.485015	-2.840079
H	0.471951	5.055768	-0.264354
H	-2.703370	1.866390	-2.393932
H	-2.918606	-3.307972	-2.348727
H	-3.988330	-2.560955	2.425113
H	-5.197039	-2.660733	-0.399392
H	-2.978673	-4.649799	0.408601
H	-1.932102	-0.360018	2.434300
H	-0.941037	-3.060545	2.159297
H	-3.827449	-0.374302	-1.990795
H	-0.976055	-1.043484	-2.192981
H	-0.316849	-3.543394	-0.697848
H	-4.459616	0.082807	0.887313
H	5.282299	0.030701	1.630484
H	4.329500	-1.579915	-2.200887
H	2.311790	-2.263796	-2.748399
H	0.922297	-2.233590	-1.679581
H	1.322381	-0.816336	-2.649846
H	3.819685	0.459360	3.173883
H	2.327380	1.218972	2.638214
H	2.301187	-0.441715	3.210788
H	6.666828	-1.560864	-1.429922
H	6.768901	0.171569	-1.114720
H	7.066900	-0.973515	0.192257

92

5$_{Mes*}$, E(B3LYP/6-311+G**)=-1706.755108

C	1.751917	-1.330800	-0.925899
C	1.175022	-0.936096	0.320132
C	2.064042	-0.493350	1.368080
C	3.312041	-0.002994	0.966694
C	3.772812	-0.058346	-0.346272
C	3.019831	-0.832139	-1.230233
P	-0.523083	-0.425847	0.895502
C	-0.728375	1.331477	0.133365
B	-1.017355	1.496239	-1.556317
B	-0.649965	3.144349	-1.980493
B	-1.730122	4.212680	-1.056009
B	-1.359280	4.006092	0.674742
B	-2.786327	3.202871	-0.041225
B	-2.344956	2.666475	-1.684592
C	-2.286165	1.588874	-0.338664
B	-1.742969	2.368918	1.123030
B	-0.062009	2.783027	0.813533
B	-0.043325	3.959590	-0.518786
B	0.378507	2.260240	-0.816478
C	1.845980	-0.688727	2.918257
C	1.066800	-1.995531	3.207141

C	5.111903	0.590916	-0.731348
C	6.264606	-0.100252	0.034924
C	1.176600	-2.442296	-1.849991
C	0.545433	-3.541236	-0.966539
C	-2.274208	-1.008793	0.200405
B	-3.306370	-0.343785	1.448892
B	-4.771052	-1.276993	1.461543
B	-5.553482	-1.096799	-0.121307
B	-4.475134	-1.802469	-1.349830
B	-4.546899	-0.033019	-1.125986
B	-4.719738	0.296556	0.611372
C	-3.168772	0.341450	-0.161506
B	-3.006926	-0.866940	-1.389510
B	-3.006867	-2.403146	-0.525207
B	-4.604104	-2.567101	0.249444
B	-3.190423	-2.079639	1.204439
C	0.165363	-1.920497	-2.882148
C	2.307970	-3.137213	-2.647858
C	3.226176	-0.884883	3.604581
C	1.174660	0.503374	3.636082
C	5.399531	0.482474	-2.240410
C	5.074525	2.090822	-0.355623
H	-2.374110	-0.637657	-2.335010
H	-2.785418	0.173682	2.353720
H	-6.727905	-1.096914	-0.250271
H	-2.589172	-2.704174	2.004779
H	-2.322100	-3.272572	-0.917940
H	-4.887729	0.760133	-1.925663
H	-4.847629	-2.299723	-2.354658
H	-5.350976	-1.393911	2.483991
H	-5.090198	-3.633772	0.398210
H	-5.177784	1.312903	0.989059
H	-1.486331	4.855291	1.486178
H	-0.271873	3.373937	-3.076242
H	-2.123681	5.238027	-1.491749
H	0.787039	4.799239	-0.567590
H	-0.902352	0.553379	-2.224890
H	1.435359	1.793925	-1.036492
H	-3.913947	3.384053	0.243370
H	-2.113941	1.977780	2.157153
H	0.702446	2.685105	1.701325
H	-3.176802	2.490169	-2.498333
H	3.964737	0.411115	1.720218
H	3.441248	-1.072314	-2.193269
H	0.121727	-4.330771	-1.593554
H	1.301823	-3.991852	-0.318049
H	-0.250995	-3.162168	-0.330569
H	1.890527	-4.018469	-3.142232
H	2.724450	-2.501630	-3.432695
H	3.123013	-3.470261	-2.000805

H	-0.146956	-2.733199	-3.544943
H	-0.726659	-1.526668	-2.410513
H	0.606270	-1.131971	-3.497625
H	1.186763	0.329624	4.716698
H	1.715870	1.432445	3.441865
H	0.138402	0.640396	3.331366
H	3.056716	-1.203702	4.635831
H	3.825498	-1.652519	3.109374
H	3.811481	0.035427	3.653235
H	1.046481	-2.168978	4.286683
H	0.032911	-1.961732	2.868361
H	1.556350	-2.853206	2.738234
H	6.024210	2.566995	-0.616948
H	4.275534	2.610165	-0.890913
H	4.911426	2.239507	0.714142
H	7.222669	0.353757	-0.235874
H	6.146619	-0.007554	1.117057
H	6.313515	-1.165401	-0.207901
H	6.328486	1.009355	-2.473702
H	5.525943	-0.554865	-2.562164
H	4.603122	0.934135	-2.838071

29

6, E(B3LYP/6-311+G**)=-788.211517

C	0.000000	0.000000	0.000000
P	0.000000	0.000000	1.343544
C	1.255597	0.000000	2.127824
C	2.640894	0.000078	1.235266
C	2.445587	0.000161	-0.232207
C	1.222095	0.000092	-0.787465
B	2.201191	1.465735	2.074253
B	3.151464	1.445031	3.549163
B	2.693268	-0.000131	4.491094
B	4.198684	-0.000037	3.521142
B	3.875680	0.892100	2.019581
B	3.875723	-0.892036	2.019436
B	2.201256	-1.465722	2.074078
B	3.151579	-1.445149	3.548984
B	1.459459	-0.892147	3.576335
B	1.459420	0.891961	3.576413
H	5.277319	-0.000040	4.004474
H	1.779077	2.346312	1.419161
H	1.779149	-2.346229	1.418888
H	3.465242	-2.475230	4.035689
H	4.636243	-1.478389	1.333215
H	0.520310	-1.478614	3.985182
H	0.520249	1.478347	3.985329
H	3.465076	2.475071	4.035990
H	4.636153	1.478572	1.333409
H	2.687585	-0.000204	5.673055

H	1.133327	0.000158	-1.867632
H	-0.947069	-0.000017	-0.526959
H	3.345333	0.000298	-0.834944

13

3-methyl-1$_{H}$, E(B3LYP/6-311+G**)=-536.142392

C	0.004891	0.021134	0.001066
C	0.005984	0.048381	1.352284
P	1.713406	0.068904	1.971326
C	2.323633	-0.252425	0.294066
C	1.323496	-0.148166	-0.616048
C	1.489357	-0.193978	-2.109193
H	1.793860	-1.229071	2.551408
H	-0.870008	0.177187	1.973798
H	-0.893468	0.116869	-0.601504
H	3.371087	-0.363793	0.044935
H	0.867598	-0.981911	-2.547505
H	1.180174	0.750940	-2.568947
H	2.527015	-0.382916	-2.389158

13

3-methylene-1$_{H}$, E(B3LYP/6-311+G**)=-536.134826

H	0.018960	-0.226635	0.026411
C	0.019898	-0.145305	1.109438
C	1.144048	-0.091184	1.841518
P	0.852802	-0.025725	3.656169
C	-1.011387	-0.075980	3.349418
C	-1.243051	-0.067626	1.842437
C	-2.449122	-0.016457	1.259863
H	1.003141	1.387423	3.811323
H	2.139465	-0.134348	1.415376
H	-1.401951	-0.999645	3.783939
H	-2.552986	-0.045480	0.180804
H	-3.362165	0.055163	1.839795
H	-1.512331	0.755261	3.848403

25

3-methyl-1$_{tBu}$, E(B3LYP/6-311+G**)=-693.443674

C	-0.016756	-0.019486	-0.028997
C	-0.026461	0.009779	1.329241
C	1.318510	0.058985	1.907211
C	2.326860	0.070585	1.003544
P	1.654580	0.237061	-0.668559
C	2.148890	-1.340349	-1.627251
C	1.354939	-1.330205	-2.946151
C	3.654330	-1.231073	-1.929225
C	1.857314	-2.628762	-0.844996
H	3.377672	0.129152	1.254690
H	1.471102	0.099374	2.981880
C	-1.253102	0.016368	2.198089

H	-0.909544	-0.025842	-0.641492
H	2.150399	-3.500228	-1.443357
H	2.413461	-2.662278	0.094143
H	0.795045	-2.722486	-0.610057
H	1.653020	-2.185631	-3.562783
H	0.278694	-1.409875	-2.770325
H	1.540205	-0.420127	-3.523239
H	3.971512	-2.085446	-2.537737
H	3.890814	-0.318635	-2.483382
H	4.252289	-1.239024	-1.013794
H	-1.218340	-0.796464	2.931655
H	-1.332642	0.952255	2.762137
H	-2.162255	-0.098026	1.605080

25

3-methylene-1$_{t\text{Bu}}$, E(B3LYP/6-311+G**)=-693.435784

C	-0.072316	0.100237	-0.051067
C	-0.003868	-0.086168	1.460789
C	1.378325	-0.096477	1.933733
C	2.333290	0.055164	0.998915
P	1.689154	0.356828	-0.686927
C	2.109670	-1.268429	-1.614471
C	1.345874	-1.228175	-2.950622
C	3.624241	-1.228903	-1.891886
C	1.757057	-2.546824	-0.842149
H	3.390726	0.087206	1.235281
H	1.591915	-0.208871	2.992930
C	-1.073760	-0.190461	2.262760
H	-0.538284	-0.759807	-0.537632
H	2.032806	-3.427508	-1.434640
H	2.289642	-2.603576	0.109765
H	0.687135	-2.621417	-0.630973
H	1.615191	-2.096609	-3.562406
H	0.261998	-1.256189	-2.803258
H	1.584736	-0.327314	-3.523050
H	3.915417	-2.091497	-2.501926
H	3.911129	-0.323367	-2.433972
H	4.206962	-1.270070	-0.967090
H	-0.962547	-0.288645	3.337092
H	-2.085055	-0.176848	1.872470
H	-0.674011	0.975517	-0.303967

23

3-methyl-1$_{\text{Ph}}$, E(B3LYP/6-311+G**)=-767.252437

C	-0.009317	0.013252	0.006535
C	0.014193	0.036521	1.407693
C	1.247124	0.005235	2.067317
C	2.438497	-0.051494	1.341515
C	2.405227	-0.074875	-0.049645
C	1.178262	-0.041907	-0.716198

P	-1.530118	0.107862	2.429607
C	-2.592660	-1.221881	1.800155
C	-3.725467	-0.700692	1.278615
C	-3.745277	0.764550	1.186484
C	-2.603708	1.336912	1.643856
H	-2.400580	-2.275462	1.952968
H	-4.571600	-1.298848	0.953110
C	-4.940749	1.497517	0.646122
H	-2.421159	2.403283	1.680405
H	1.275899	0.026279	3.151370
H	3.387943	-0.075555	1.864791
H	3.329283	-0.117674	-0.615417
H	1.149200	-0.059556	-1.800267
H	-0.960150	0.038962	-0.513864
H	-5.209664	1.128421	-0.349574
H	-5.816076	1.350028	1.288221
H	-4.751771	2.569894	0.573906

23

3-methylene-1$_{Ph}$, E(B3LYP/6-311+G**)=-767.246515

C	0.033026	-0.271657	0.030967
C	0.008415	-0.067535	1.417520
C	1.219256	0.146934	2.087179
C	2.426537	0.171531	1.388479
C	2.437414	-0.029215	0.010264
C	1.237973	-0.253029	-0.667175
P	-1.550073	-0.063320	2.430175
C	-2.616826	-1.249384	1.535554
C	-3.704258	-0.688732	0.977768
C	-3.807528	0.766324	1.078299
C	-2.544052	1.366345	1.680118
H	-2.434687	-2.317674	1.554596
H	-4.493512	-1.261990	0.499892
C	-4.890377	1.471595	0.720543
H	-2.775715	2.114845	2.439067
H	1.215668	0.292482	3.162536
H	3.355567	0.341841	1.921326
H	3.374871	-0.015696	-0.534533
H	1.242622	-0.415191	-1.739665
H	-0.893471	-0.451332	-0.503968
H	-5.769608	0.982759	0.315488
H	-4.930934	2.549665	0.825141
H	-1.937357	1.847649	0.908842

32

3-methyl-1$_{Mes}$, E(B3LYP/6-311+G**)=-885.227984

C	0.005557	0.277125	-0.012106
C	0.000744	0.193488	1.382371
C	1.236425	0.131382	2.070309
C	2.444907	0.152407	1.334748

C	2.393495	0.236371	-0.059661
C	1.186778	0.303115	-0.753077
C	-1.328318	0.167766	2.092794
P	1.390531	0.021128	3.914154
C	0.388019	1.263342	4.745425
C	-0.429728	0.670872	5.659599
C	-1.239407	1.391446	6.702020
C	3.809047	0.083809	1.990568
C	1.157255	0.426909	-2.256701
C	0.359336	-1.294883	4.583223
C	-0.439110	-0.784712	5.555877
H	0.498002	-2.347721	4.376972
H	-1.031864	-1.396968	6.228922
H	0.561423	2.329464	4.674187
H	3.325487	0.248190	-0.616548
H	-0.946614	0.320964	-0.532694
H	-1.455682	-0.754235	2.665157
H	-1.415023	0.991950	2.804603
H	-2.148326	0.242906	1.376420
H	4.593396	0.121401	1.232025
H	3.970411	0.912730	2.684148
H	3.939844	-0.836412	2.565425
H	0.289785	-0.083641	-2.681933
H	1.098278	1.478128	-2.559829
H	2.057427	0.005446	-2.709642
H	-2.292994	1.095218	6.654508
H	-0.885895	1.155219	7.711701
H	-1.183650	2.473735	6.571573

32

3-methylene-1$_{\mathrm{Mes}}$, E(B3LYP/6-311+G**)=-885.217333

C	0.053750	-0.095151	0.003263
C	0.038257	-0.063043	1.400455
C	1.266861	-0.045022	2.107385
C	2.478300	-0.060910	1.371005
C	2.437947	-0.090499	-0.026183
C	1.237943	-0.107036	-0.732036
C	-1.317195	-0.066045	2.071744
P	1.469826	-0.008392	3.963453
C	0.576822	1.549763	4.561833
C	-0.435894	1.093260	5.604926
C	-1.041762	1.907678	6.482463
C	3.845655	-0.043743	2.027361
C	1.217296	-0.108802	-2.240785
C	0.156531	-1.035077	4.704577
C	-0.632221	-0.354854	5.555857
H	0.129598	-2.113126	4.594088
H	-1.366127	-0.829418	6.200587
H	0.084879	2.041724	3.719480
H	3.374893	-0.103417	-0.574887

H	-0.895064	-0.112051	-0.525307
H	-1.497363	-0.998159	2.611654
H	-1.431809	0.741257	2.796165
H	-2.104812	0.044749	1.323992
H	4.626251	-0.073642	1.264406
H	4.001037	0.855419	2.629132
H	3.989566	-0.894930	2.696273
H	0.365620	-0.673734	-2.627525
H	1.136247	0.911850	-2.630922
H	2.130707	-0.544799	-2.651563
H	-1.737057	1.520277	7.219115
H	-0.858218	2.975883	6.492755
H	1.292021	2.257325	4.983436

59

3-methyl-1$_{Mes*}$, E(B3LYP/6-311+G**)=-1239.093654

C	-0.164760	-1.062885	-0.363117
C	0.229915	0.302475	-0.263182
C	-0.787729	1.314062	-0.160875
C	-2.101818	0.907002	0.057977
C	-2.479302	-0.431192	0.187275
C	-1.503301	-1.379435	-0.072310
P	1.936675	0.865184	0.226679
C	3.308163	0.917485	-0.918942
C	4.398444	0.382438	-0.299478
C	4.129522	-0.274050	0.967501
C	2.806913	-0.242661	1.317321
C	-0.547053	2.852568	-0.306935
C	0.456193	3.166293	-1.442385
C	-3.930479	-0.792081	0.549037
C	-4.885908	-0.257123	-0.543450
C	0.688482	-2.287812	-0.820244
C	-0.136384	-3.112398	-1.850604
C	-1.852456	3.587588	-0.706704
C	-0.077479	3.484507	1.026622
C	-4.145443	-2.311779	0.671732
C	-4.292667	-0.145738	1.906944
C	1.993196	-1.929020	-1.549402
C	1.014591	-3.198702	0.385076
H	3.324565	1.464472	-1.850692
H	5.405763	0.458706	-0.696786
C	5.230922	-0.869691	1.801288
H	2.405807	-0.623097	2.247021
H	-2.867738	1.659655	0.157659
H	-1.782018	-2.421081	-0.058519
H	1.551568	-4.090908	0.046895
H	1.645901	-2.680443	1.108338
H	0.106387	-3.528055	0.896868
H	0.490816	-3.916431	-2.245150
H	-1.025743	-3.580082	-1.427225

H	-0.450501	-2.487246	-2.690970
H	2.406426	-2.837431	-1.998169
H	1.818163	-1.212776	-2.355599
H	2.750711	-1.528276	-0.886313
H	-1.611093	4.629858	-0.929789
H	-2.311833	3.152228	-1.598196
H	-2.594459	3.602645	0.094410
H	0.020188	4.568862	0.908614
H	-0.810818	3.296882	1.816218
H	0.884241	3.090504	1.352272
H	0.487592	4.246265	-1.614350
H	1.473094	2.857685	-1.210285
H	0.151294	2.685000	-2.375894
H	-5.324123	-0.389718	2.179713
H	-3.635574	-0.511575	2.700805
H	-4.204561	0.942545	1.875808
H	-5.181972	-2.512873	0.956001
H	-3.959415	-2.828890	-0.273543
H	-3.501686	-2.753139	1.437569
H	-5.922232	-0.507967	-0.296316
H	-4.821623	0.828636	-0.644701
H	-4.652138	-0.698483	-1.516253
H	5.843501	-1.557932	1.209049
H	5.901584	-0.094271	2.187702
H	4.830424	-1.420138	2.654766

59

3-methylene-1$_{Mes*}$, E(B3LYP/6-311+G**)=-1239.078286

C	-0.120473	-1.047695	-0.393074
C	0.282709	0.315978	-0.277806
C	-0.749265	1.315302	-0.184286
C	-2.053726	0.905668	0.085614
C	-2.420164	-0.432928	0.236543
C	-1.450228	-1.373543	-0.072848
P	1.944248	0.964414	0.346665
C	3.381521	0.809955	-0.763556
C	4.433562	0.229827	-0.156412
C	4.176322	-0.357386	1.158787
C	2.690326	-0.327565	1.494699
C	-0.539958	2.850320	-0.412438
C	0.549783	3.138692	-1.472483
C	-3.852718	-0.802263	0.657867
C	-4.858155	-0.275741	-0.392935
C	0.679117	-2.255887	-0.977868
C	-0.123532	-2.767760	-2.209831
C	-1.833321	3.491392	-0.983756
C	-0.216203	3.588963	0.908423
C	-4.051798	-2.323107	0.792635
C	-4.160363	-0.155397	2.028893
C	2.080403	-1.936325	-1.514530

C	0.799587	-3.413370	0.041753
H	3.442509	1.304792	-1.725495
H	5.433793	0.211556	-0.578822
C	5.130342	-0.822182	1.980189
H	2.234657	-1.304018	1.331459
H	-2.818936	1.657912	0.201911
H	-1.729720	-2.416354	-0.085918
H	1.321015	-4.259047	-0.417067
H	1.367103	-3.117554	0.927438
H	-0.175038	-3.771039	0.379981
H	0.412292	-3.601786	-2.672922
H	-1.123548	-3.117663	-1.952009
H	-0.228174	-1.976218	-2.956928
H	2.440970	-2.794398	-2.089850
H	2.070638	-1.072398	-2.181172
H	2.808439	-1.754682	-0.731365
H	-1.613751	4.520354	-1.280071
H	-2.197064	2.957107	-1.865548
H	-2.643202	3.543170	-0.253643
H	-0.151009	4.667027	0.725956
H	-1.007291	3.420798	1.644962
H	0.728269	3.255094	1.335679
H	0.543273	4.204372	-1.719919
H	1.553945	2.905307	-1.123085
H	0.359880	2.578436	-2.392317
H	-5.178304	-0.403218	2.345649
H	-3.468473	-0.516823	2.794722
H	-4.077905	0.933162	1.991842
H	-5.072345	-2.530838	1.125846
H	-3.907715	-2.840875	-0.159674
H	-3.368986	-2.758609	1.527372
H	-5.881319	-0.532274	-0.100693
H	-4.804856	0.810131	-0.499029
H	-4.664166	-0.717662	-1.374236
H	6.176485	-0.794451	1.695611
H	4.895412	-1.234442	2.954641
H	2.516806	-0.050779	2.535248

15

3-methyl-3$_H$, E(B3LYP/6-311+G**)=-537.364301

C	0.000537	0.010137	-0.002949
P	-0.021685	-0.010444	1.891401
C	1.811574	0.010964	1.889730
C	2.366710	-0.289766	0.709162
C	1.390718	-0.516384	-0.428903
H	2.389225	0.137127	2.799500
C	3.834937	-0.474595	0.456896
H	1.737994	-0.034796	-1.350544
H	-0.809381	-0.599397	-0.405062
H	-0.262738	1.378629	2.120998

H	-0.145445	1.033107	-0.353064
H	1.345314	-1.591263	-0.650879
H	4.424666	-0.310112	1.360480
H	4.045239	-1.486610	0.090684
H	4.188036	0.217834	-0.316093

15

3-methylene-3$_{\mathrm{H}}$, E(B3LYP/6-311+G**)=-537.359713

C	0.069293	0.007904	0.013796
P	0.092680	0.567868	1.830870
C	1.714430	-0.346974	2.139443
C	2.387521	-0.437798	0.796029
C	1.369697	-0.789615	-0.266604
H	1.455535	-1.344164	2.513969
C	3.673178	-0.170741	0.565087
H	1.755972	-0.604109	-1.271241
H	-0.821170	-0.595349	-0.169057
H	0.703294	1.832781	1.590355
H	-0.002457	0.892921	-0.620309
H	1.153772	-1.863344	-0.201205
H	4.353674	0.089684	1.368640
H	4.088058	-0.207167	-0.436626
H	2.320961	0.148741	2.898494

27

3-methyl-3$_{t\mathrm{Bu}}$, E(B3LYP/6-311+G**)=-694.664979

C	-0.082424	-0.094476	0.142774
C	-0.019646	-0.027141	1.687626
C	1.427205	0.096797	2.118527
C	1.761071	-0.014331	3.578052
P	1.549306	0.600146	-0.512840
C	2.300009	0.361557	1.136733
H	3.353198	0.541355	1.328026
H	-0.496256	-0.897743	2.154443
H	-0.921075	0.478164	-0.254682
C	2.286645	-0.822653	-1.566116
H	-0.206017	-1.126233	-0.192604
H	-0.573794	0.847299	2.053847
H	2.829680	0.113895	3.759996
H	1.221954	0.740792	4.162671
H	1.458551	-0.990796	3.974222
C	3.715345	-0.384967	-1.940699
C	2.335905	-2.181469	-0.854949
C	1.432946	-0.922658	-2.843155
H	1.864608	-1.661028	-3.528679
H	0.408187	-1.238230	-2.624759
H	1.385331	0.035250	-3.369346
H	2.784405	-2.933161	-1.515851
H	2.934303	-2.136744	0.057980
H	1.340762	-2.544326	-0.584144

H	4.169426	-1.119779	-2.615590
H	3.718379	0.584057	-2.447539
H	4.359088	-0.307394	-1.059450

27

3-methylene-3$_{t\mathrm{Bu}}$, E(B3LYP/6-311+G**)=-694.656790

C	-0.036047	-0.235957	0.298739
C	-0.117128	0.322276	1.735553
C	1.280749	0.291993	2.308247
C	1.600720	-0.190277	3.508975
P	1.607473	0.368956	-0.427094
C	2.266974	0.820151	1.293354
H	2.298929	1.914402	1.334414
H	-0.826809	-0.231664	2.354921
H	-0.866365	0.105754	-0.321304
C	2.531436	-1.248263	-0.860943
H	-0.064011	-1.327792	0.316364
H	-0.464171	1.363492	1.699751
H	2.626646	-0.209565	3.861354
H	0.844129	-0.578508	4.182897
H	3.285013	0.464032	1.461161
C	3.948057	-0.833342	-1.303497
C	2.630999	-2.277961	0.274304
C	1.790504	-1.866259	-2.062201
H	2.332384	-2.747819	-2.423256
H	0.779783	-2.189441	-1.796534
H	1.711316	-1.158410	-2.892398
H	3.176590	-3.162543	-0.076255
H	3.166300	-1.884418	1.141055
H	1.650340	-2.617327	0.615942
H	4.505782	-1.711191	-1.649140
H	3.916094	-0.109961	-2.122861
H	4.517337	-0.386570	-0.482894

25

3-methyl-3$_{\mathrm{Ph}}$, E(B3LYP/6-311+G**)=-768.476328

C	-0.025573	-0.267057	0.468399
P	0.495582	0.192075	2.231336
C	2.227666	0.302586	1.656453
C	2.456952	-0.273633	0.468237
C	1.231609	-0.839791	-0.221980
H	3.020007	0.689501	2.289073
C	3.803566	-0.458111	-0.168567
H	1.242044	-0.620625	-1.296294
H	-0.852916	-0.977666	0.480728
C	-0.032085	1.970313	2.356682
H	-0.366530	0.637812	-0.039010
H	1.251316	-1.934848	-0.138378
H	4.601415	-0.025074	0.437431
H	4.024316	-1.522309	-0.314304

H	3.832494	0.005678	-1.161401
C	-1.030901	2.292197	3.283828
C	-1.485210	3.604332	3.418740
C	-0.933873	4.616186	2.636053
C	0.070690	4.310658	1.716781
C	0.515973	2.998214	1.577272
H	-1.451953	1.509481	3.906705
H	-2.262651	3.835510	4.138740
H	-1.280049	5.638193	2.743738
H	0.507611	5.096498	1.109772
H	1.299572	2.769733	0.862512

25

3-methylene-3$_{Ph}$, E(B3LYP/6-311+G**)=-768.468455

C	-0.103916	-0.420282	0.393205
P	0.145898	-0.029038	2.237056
C	2.030013	-0.182339	2.062835
C	2.341228	-0.237146	0.587729
C	1.237403	-0.966378	-0.142218
H	2.305397	-1.129258	2.540775
C	3.395029	0.334484	0.003559
H	1.322025	-0.861026	-1.226521
H	-0.918102	-1.136982	0.273893
C	-0.095395	1.812141	2.283372
H	-0.386201	0.495128	-0.130434
H	1.309356	-2.038253	0.084456
H	4.144509	0.868453	0.577963
H	3.545907	0.279085	-1.069565
H	2.551129	0.617984	2.589807
C	-0.946170	2.316025	3.275652
C	-1.182837	3.686381	3.391229
C	-0.565237	4.575419	2.515741
C	0.287995	4.088664	1.524318
C	0.519036	2.720274	1.407099
H	-1.426233	1.627171	3.962895
H	-1.847328	4.056037	4.164502
H	-0.745806	5.641101	2.603011
H	0.773569	4.776920	0.840753
H	1.186537	2.361492	0.631713

34

3-methyl-3$_{Mes}$, E(B3LYP/6-311+G**)=-886.446415

C	0.003415	-0.146699	0.140064
P	0.325788	0.216276	1.968920
C	2.117851	0.031479	1.673667
C	2.418148	-0.613115	0.538354
C	1.237234	-0.933845	-0.358639
H	2.856700	0.261300	2.434712
C	3.785239	-1.081192	0.133462
H	1.465530	-0.712457	-1.408579

H	-0.919850	-0.715734	0.024345
C	-0.065549	2.032381	2.184914
H	-0.116763	0.789589	-0.407579
H	1.043231	-2.014281	-0.316698
H	4.541616	-0.810888	0.872718
H	3.806808	-2.170355	0.006446
H	4.075993	-0.648683	-0.831539
C	-1.129273	2.315522	3.078734
C	-1.503084	3.641861	3.314909
C	-0.857341	4.713086	2.703422
C	0.183670	4.423190	1.823287
C	0.593098	3.114757	1.549328
C	-1.910085	1.234232	3.801217
H	-2.324315	3.838695	3.997723
C	-1.252551	6.138773	3.000810
H	0.696229	5.241705	1.325784
C	1.739940	2.947703	0.576486
H	-2.660706	1.687221	4.452395
H	-2.429356	0.568780	3.106414
H	-1.262330	0.601809	4.411791
H	-1.103360	6.783893	2.131483
H	-2.300753	6.207870	3.300742
H	-0.649697	6.548631	3.818851
H	2.633665	2.557699	1.067917
H	1.504711	2.256837	-0.234185
H	1.994941	3.911503	0.131178

34

3-methylene-3$_{Mes}$, E(B3LYP/6-311+G**)=-886.437030

C	-0.003901	-0.324685	0.307063
P	0.270386	0.122428	2.124430
C	2.144755	-0.143321	1.975727
C	2.422404	-0.654129	0.575322
C	1.176233	-1.251345	-0.043526
H	2.392208	-0.915632	2.710992
C	3.595571	-0.549902	-0.048732
H	1.286220	-1.394241	-1.121352
H	-0.966942	-0.824922	0.191473
C	-0.067625	1.956546	2.230034
H	-0.008934	0.563097	-0.328494
H	0.997654	-2.241788	0.396563
H	4.457396	-0.094732	0.427958
H	3.733986	-0.921706	-1.058785
H	2.734902	0.737532	2.228537
C	-1.133213	2.313481	3.099544
C	-1.486162	3.656438	3.249133
C	-0.822656	4.678381	2.572636
C	0.216487	4.317616	1.720073
C	0.610150	2.987334	1.535667
C	-1.937122	1.294446	3.884641

H	-2.307892	3.908399	3.912979
C	-1.204212	6.124077	2.775890
H	0.743626	5.094063	1.172703
C	1.778087	2.756405	0.602955
H	1.707559	1.832523	0.034232
H	1.860454	3.581868	-0.107371
H	2.722221	2.716434	1.156410
H	-2.677872	1.802695	4.505604
H	-2.469447	0.598801	3.230805
H	-1.303784	0.686704	4.534103
H	-0.910380	6.738190	1.921637
H	-2.281577	6.235558	2.921462
H	-0.711047	6.537136	3.662823

61

3-methyl-3$_{Mes*}$, E(B3LYP/6-311+G**)=-1240.309324

C	-0.125634	-1.008077	0.411324
C	0.214604	0.369936	0.258471
C	-0.865848	1.315185	0.158049
C	-2.155092	0.839840	-0.072862
C	-2.459307	-0.517215	-0.197842
C	-1.439731	-1.405218	0.107455
P	1.834452	1.042885	-0.455696
C	2.670672	-0.298076	-1.350458
C	3.976328	-0.415420	-1.071279
C	4.486627	0.450579	0.064822
C	3.299686	1.184461	0.740434
C	-0.723118	2.864939	0.314160
C	-0.521568	3.551681	-1.057823
C	-3.878967	-0.961096	-0.589863
C	-4.242801	-0.348401	-1.962807
C	0.756306	-2.161300	0.986180
C	1.116668	-3.196569	-0.103272
C	-2.008703	3.463319	0.945918
C	0.418244	3.259611	1.278954
C	-4.006031	-2.491499	-0.702362
C	-4.890559	-0.470729	0.472490
C	2.042094	-1.694104	1.685369
C	-0.059638	-2.886469	2.096087
H	2.194539	-0.816457	-2.175618
C	4.954577	-1.277249	-1.814266
H	5.059799	-0.150484	0.782233
H	3.522983	2.241929	0.886208
H	3.062842	0.766017	1.716028
H	5.197153	1.181848	-0.341572
H	-2.957080	1.553299	-0.188578
H	-1.663997	-2.460957	0.125786
H	0.574963	-3.638095	2.574740
H	-0.386118	-2.180475	2.864570
H	-0.942363	-3.402554	1.717941

H	2.491843	-2.542381	2.210101
H	2.778394	-1.322350	0.983048
H	1.826656	-0.922080	2.428098
H	1.659636	-4.037198	0.341385
H	0.221809	-3.596658	-0.586773
H	1.751084	-2.757142	-0.873654
H	-5.020886	-2.753060	-1.014686
H	-3.315440	-2.902806	-1.443766
H	-3.819866	-2.988847	0.253499
H	-5.252978	-0.649597	-2.257794
H	-4.212829	0.743264	-1.940789
H	-3.547638	-0.685632	-2.736684
H	-5.905346	-0.779453	0.201896
H	-4.658015	-0.890599	1.455097
H	-4.887243	0.617693	0.565164
H	-1.829183	4.515754	1.181315
H	-2.868789	3.436033	0.274687
H	-2.279911	2.951447	1.873278
H	-0.477796	4.638820	-0.929920
H	0.399857	3.222178	-1.537024
H	-1.357124	3.325767	-1.726490
H	0.358010	4.330384	1.495335
H	0.339708	2.719615	2.226606
H	1.406251	3.082126	0.859865
H	5.447572	-1.982738	-1.134418
H	4.470410	-1.847359	-2.609260
H	5.749447	-0.671302	-2.265681

61

3-methylene-3$_{\mathrm{Mes*}}$, E(B3LYP/6-311+G**)=-1240.296560

C	-0.126626	-1.029591	0.381586
C	0.250401	0.341594	0.247620
C	-0.808687	1.315236	0.173168
C	-2.109894	0.876120	-0.064033
C	-2.452223	-0.469928	-0.201663
C	-1.455156	-1.386697	0.090916
P	1.874946	1.012061	-0.455180
C	2.632864	-0.339812	-1.540689
C	4.087070	-0.478043	-1.121189
C	4.536792	0.688828	-0.265875
C	3.381431	1.063096	0.685720
C	-0.635514	2.858329	0.371055
C	-0.419040	3.581142	-0.979750
C	-3.885162	-0.871469	-0.590382
C	-4.237500	-0.236617	-1.956228
C	0.706685	-2.218529	0.958618
C	0.851310	-3.376066	-0.057537
C	-1.911017	3.463600	1.017631
C	0.509955	3.203331	1.349881
C	-4.054030	-2.396782	-0.715886

C	-4.878416	-0.363110	0.480757
C	2.097828	-1.854785	1.490087
C	-0.070215	-2.754559	2.197260
H	2.102350	-1.289083	-1.507546
C	4.861365	-1.516206	-1.436448
H	5.458046	0.461145	0.276088
H	3.499359	2.075233	1.076016
H	3.333419	0.380494	1.530221
H	4.752217	1.544619	-0.919628
H	-2.892289	1.612332	-0.169416
H	-1.710536	-2.435565	0.113834
H	0.505466	-3.556385	2.669578
H	-0.214552	-1.960825	2.935214
H	-1.051357	-3.156729	1.943464
H	2.518415	-2.723563	2.005359
H	2.799707	-1.585656	0.707224
H	2.036391	-1.040946	2.215001
H	1.372900	-4.217075	0.409790
H	-0.117029	-3.740679	-0.406270
H	1.428056	-3.079508	-0.936095
H	-5.076671	-2.628016	-1.026590
H	-3.377489	-2.819987	-1.463517
H	-3.878365	-2.907709	0.234835
H	-5.256897	-0.506975	-2.249187
H	-4.177167	0.853559	-1.924633
H	-3.555403	-0.586133	-2.736235
H	-5.902212	-0.642239	0.212171
H	-4.653165	-0.797358	1.458809
H	-4.845632	0.724000	0.582501
H	-1.709525	4.504059	1.285784
H	-2.769371	3.476167	0.343692
H	-2.196203	2.929701	1.928193
H	-0.361789	4.663384	-0.819950
H	0.500427	3.253550	-1.463539
H	-1.254787	3.386066	-1.657929
H	0.470097	4.268740	1.595352
H	0.416732	2.639465	2.282233
H	1.494285	3.015999	0.927434
H	5.890589	-1.576895	-1.097821
H	4.496552	-2.337667	-2.044185
H	2.567922	0.025065	-2.570269

31

3-methyl-4$_H$, E(B3LYP/6-311+G**)=-789.671454

C	-0.016720	-0.010030	-0.009197
B	-0.004517	0.019310	1.700575
B	1.514346	0.026371	0.793713
B	1.342177	-1.056802	2.160459
B	-0.339221	-1.659792	2.174806
B	0.974052	-2.686492	1.529691

B	2.114084	-1.636729	0.655002
B	0.917073	-2.605645	-0.244847
B	-0.604190	-2.619801	0.690815
B	-1.200710	-0.937670	0.791568
B	-0.439828	-1.555799	-0.687307
C	1.186997	-0.956511	-0.606199
C	1.677619	-0.363490	-1.899659
C	1.051053	0.758923	-2.279717
P	-0.209460	1.469865	-1.145037
H	-1.350512	-3.533923	0.629608
H	1.978918	-0.864206	3.136928
H	-0.906765	-1.903033	3.182379
H	1.349524	-3.664996	2.075857
H	2.187113	0.977191	0.623884
H	3.267961	-1.802902	0.475515
H	-0.330199	1.019769	2.233656
H	-2.323772	-0.581030	0.729073
H	-0.956011	-1.558660	-1.744192
H	1.275379	-3.414854	-1.024776
C	2.790465	-1.043229	-2.639291
H	1.323434	1.288115	-3.186161
H	-1.372078	1.009937	-1.828245
H	2.518214	-2.072694	-2.891284
H	3.024711	-0.508852	-3.560613
H	3.694771	-1.093064	-2.024803

31

3-methylene-4$_{H}$, E(B3LYP/6-311+G**)=-789.663494

C	0.021994	-0.001123	0.015625
B	0.011123	-0.001388	1.723774
B	1.539749	-0.027757	0.831720
B	1.316047	-1.127558	2.181903
B	-0.387219	-1.674896	2.164082
B	0.900675	-2.735359	1.521492
B	2.084787	-1.711822	0.679971
B	0.871806	-2.626898	-0.250679
B	-0.661184	-2.602347	0.659199
B	-1.202602	-0.904083	0.784180
B	-0.435853	-1.513350	-0.696910
C	1.203376	-0.979739	-0.584175
C	1.770784	-0.403432	-1.849506
C	0.932280	0.727892	-2.426173
P	-0.147852	1.517271	-1.075627
H	-1.439907	-3.486712	0.570906
H	1.945312	-0.972623	3.169748
H	-0.976490	-1.914638	3.159957
H	1.235959	-3.733550	2.058113
H	2.245021	0.903994	0.687061
H	3.235758	-1.906428	0.512674
H	-0.290226	0.999150	2.270791

H	-2.312498	-0.510454	0.712164
H	-0.941406	-1.484571	-1.759535
H	1.214683	-3.423542	-1.049667
C	2.902482	-0.842941	-2.394003
H	1.574857	1.487720	-2.872096
H	-1.408100	1.154624	-1.623181
H	3.464071	-1.660285	-1.959564
H	3.295640	-0.395816	-3.299479
H	0.276667	0.343081	-3.210671

43

3-methyl-4$_{t\mathrm{Bu}}$, E(B3LYP/6-311+G**)=-946.966008

C	0.004893	0.008230	-0.005014
B	0.014079	0.024749	1.707693
B	1.538968	0.018491	0.814870
B	1.351597	-1.060238	2.179113
B	-0.333073	-1.651596	2.182998
B	0.978562	-2.687721	1.547935
B	2.128091	-1.644069	0.678273
B	0.933617	-2.605154	-0.224979
B	-0.592384	-2.610143	0.699005
B	-1.181673	-0.926100	0.793026
B	-0.410602	-1.547214	-0.678664
C	1.208365	-0.954012	-0.590738
C	1.704990	-0.374118	-1.887093
C	1.112526	0.773689	-2.254728
P	-0.033489	1.569848	-1.073046
C	-1.701028	1.778314	-1.987570
C	-2.709468	2.293784	-0.942742
C	-1.405314	2.904459	-3.003553
C	-2.274879	0.564250	-2.726553
H	-1.345127	-3.518899	0.630788
H	1.980869	-0.868879	3.160927
H	-0.908642	-1.888579	3.187860
H	1.343438	-3.669476	2.095907
H	2.220235	0.964328	0.656110
H	3.282535	-1.812946	0.503180
H	-0.308327	1.024630	2.244642
H	-2.307797	-0.580395	0.738835
H	-0.899722	-1.591006	-1.741693
H	1.288351	-3.417806	-1.003105
C	2.788240	-1.087479	-2.639092
H	1.408837	1.292450	-3.159879
H	-3.146280	0.881704	-3.311275
H	-1.551940	0.125405	-3.417979
H	-2.608926	-0.213295	-2.040045
H	-3.646365	2.563275	-1.442147
H	-2.940988	1.535128	-0.191925
H	-2.337471	3.182626	-0.426106
H	-2.343310	3.217282	-3.473768

H	-0.963866	3.784214	-2.526589
H	-0.734871	2.571267	-3.800522
H	2.485003	-2.108573	-2.889070
H	3.029360	-0.559098	-3.562085
H	3.697867	-1.164325	-2.035109

43

3-methylene-4$_{t\mathrm{Bu}}$, E(B3LYP/6-311+G**)=-946.958248

C	0.047551	-0.009913	-0.068225
B	-0.092939	-0.017080	1.637142
B	1.504431	-0.005552	0.877947
B	1.202786	-1.105249	2.209538
B	-0.474817	-1.700396	2.057419
B	0.890154	-2.726295	1.528629
B	2.107688	-1.669694	0.779558
B	1.004016	-2.621615	-0.239170
B	-0.597589	-2.640854	0.541124
B	-1.197911	-0.960870	0.611709
B	-0.294254	-1.548937	-0.797227
C	1.307925	-0.963763	-0.556474
C	1.950136	-0.379369	-1.784721
C	1.075894	0.661465	-2.457748
P	0.070517	1.563011	-1.132937
C	-1.644843	1.839740	-1.922237
C	-2.537274	2.434846	-0.814434
C	-1.375083	2.925155	-2.989158
C	-2.352386	0.647438	-2.578549
H	-1.340842	-3.547948	0.393683
H	1.742866	-0.924222	3.244863
H	-1.137683	-1.952323	3.003125
H	1.208567	-3.713681	2.095120
H	2.194310	0.943156	0.795054
H	3.274025	-1.828971	0.707525
H	-0.466748	0.972487	2.159830
H	-2.317345	-0.621198	0.467358
H	-0.700579	-1.573117	-1.897910
H	1.430581	-3.409816	-1.005725
C	3.159627	-0.739325	-2.207010
H	0.395379	0.172475	-3.161048
H	-3.277106	0.999711	-3.049977
H	-1.751662	0.182525	-3.363898
H	-2.625714	-0.120423	-1.855612
H	-3.492437	2.754010	-1.245144
H	-2.754409	1.705856	-0.030684
H	-2.071969	3.307275	-0.347821
H	-2.328406	3.272525	-3.400635
H	-0.859180	3.793364	-2.568741
H	-0.778638	2.542323	-3.822450
H	3.741051	-1.495281	-1.694403
H	3.599426	-0.285287	-3.087167
H	1.684681	1.373183	-3.015764

41

3-methyl-4$_{Ph}$, E(B3LYP/6-311+G**)=-1020.780874

C	0.007632	0.001599	-0.012993
C	0.004897	0.013392	1.387743
C	1.230161	0.043845	2.068721
C	2.430897	0.095595	1.365025
C	2.422463	0.086163	-0.028651
C	1.210344	0.032129	-0.715235
P	-1.513139	-0.112702	2.432717
C	-2.862000	-0.424684	1.233934
C	-3.790279	0.528517	1.059415
C	-3.530991	1.810539	1.804376
B	-3.607473	1.647765	3.535813
B	-2.186704	2.521295	4.119466
B	-3.802910	3.278770	4.140645
B	-2.370656	4.222794	3.643403
B	-3.903353	4.378890	2.736928
B	-4.659349	2.770594	2.655488
B	-3.766648	3.412064	1.252261
B	-2.347143	4.309536	1.858922
B	-1.290413	3.149738	2.715891
B	-2.148376	2.687203	1.234659
C	-2.124841	1.665039	2.638818
H	-1.898074	5.221035	1.255420
H	-4.387031	3.457615	5.152265
H	-1.932108	5.090829	4.315186
H	-4.563865	5.358905	2.759185
H	-3.969532	0.607935	3.951400
H	-5.804806	2.504860	2.559611
H	-1.602982	2.068920	5.039565
H	-0.111578	3.131477	2.704304
H	-1.629371	2.298098	0.256456
H	-4.318650	3.575280	0.222322
C	-5.029025	0.408108	0.224104
H	-2.953679	-1.399651	0.767454
H	1.244594	0.024482	3.153610
H	3.370632	0.131167	1.904333
H	3.356779	0.114846	-0.577837
H	1.200192	0.019420	-1.799453
H	-0.929200	-0.028062	-0.556255
H	-5.060241	1.188626	-0.541957
H	-5.072459	-0.565288	-0.265439
H	-5.926557	0.529471	0.838569

41

3-methylene-4$_{Ph}$, E(B3LYP/6-311+G**)=-1020.771032

C	0.259278	0.281842	-0.015658
C	0.033479	0.005381	1.338859
C	1.141006	-0.227152	2.168499

C	2.438444	-0.159027	1.666041
C	2.647848	0.121238	0.317942
C	1.555789	0.336160	-0.521611
P	-1.603885	-0.145200	2.186552
C	-2.870763	-0.365614	0.799909
C	-4.039000	0.579747	1.020027
C	-3.641514	1.840242	1.737094
B	-3.556371	1.670042	3.463188
B	-2.060629	2.499920	3.915653
B	-3.644232	3.305936	4.088640
B	-2.233516	4.207797	3.460911
B	-3.839781	4.416940	2.703545
B	-4.649692	2.834662	2.690704
B	-3.875497	3.454927	1.212142
B	-2.377288	4.301108	1.680871
B	-1.280857	3.108681	2.436267
B	-2.292662	2.668022	1.048383
C	-2.162746	1.648343	2.438505
H	-1.955014	5.197598	1.036846
H	-4.125217	3.496971	5.150985
H	-1.707007	5.058889	4.089842
H	-4.464655	5.416600	2.790058
H	-3.908190	0.640684	3.912122
H	-5.805899	2.602370	2.707209
H	-1.407861	2.028756	4.778250
H	-0.110076	3.054809	2.311711
H	-1.878131	2.273465	0.022357
H	-4.511958	3.623828	0.233660
C	-5.289109	0.330716	0.638417
H	-3.209674	-1.401899	0.779117
H	0.983746	-0.465618	3.215296
H	3.281343	-0.333973	2.324857
H	3.655623	0.166899	-0.079070
H	1.712537	0.550716	-1.572805
H	-0.566900	0.462605	-0.691476
H	-6.088106	1.041845	0.806195
H	-5.544990	-0.596819	0.139935
H	-2.402826	-0.152532	-0.163784

50

3-methyl-4$_{Mes}$, E(B3LYP/6-311+G**)=-1138.751281

C	0.002929	0.016665	-0.004700
C	0.001755	0.007799	1.412133
C	1.243053	0.007347	2.101799
C	2.433854	0.073353	1.376257
C	2.451723	0.117821	-0.015094
C	1.227061	0.074572	-0.677784
P	-1.472349	-0.143458	2.539481
C	-3.021019	-0.207907	1.571790
C	-3.896719	0.799321	1.707569

C	-3.402246	1.963097	2.519623
B	-3.197987	1.603026	4.208205
B	-1.635234	2.312757	4.619817
B	-3.155425	3.161556	5.006427
B	-1.756252	4.066489	4.365544
B	-3.401711	4.423535	3.766029
B	-4.287188	2.885342	3.651683
B	-3.595628	3.631505	2.187793
B	-2.027754	4.361607	2.624688
B	-0.940377	3.046230	3.153613
B	-2.068111	2.814072	1.808686
C	-1.889939	1.630261	3.067719
C	1.346987	-0.058197	3.610721
C	3.748624	0.223115	-0.777543
C	-1.236290	-0.073668	-0.868049
H	-1.612788	5.307723	2.050572
H	-3.543190	3.255512	6.118895
H	-1.139339	4.819472	5.036292
H	-3.967909	5.432514	4.008456
H	-3.568738	0.547103	4.572060
H	-5.450276	2.705336	3.733029
H	-0.946664	1.725802	5.376414
H	0.213988	2.963881	2.929984
H	-1.747663	2.512618	0.720082
H	-4.298839	3.947873	1.294592
C	-5.285264	0.837486	1.143572
H	-3.284074	-1.113241	1.037672
H	3.373276	0.080828	1.920372
H	1.217188	0.083196	-1.763403
H	-1.982227	0.676944	-0.610894
H	-0.967731	0.064620	-1.916665
H	-1.713536	-1.054328	-0.783941
H	2.384113	-0.220804	3.908747
H	1.013329	0.868173	4.085256
H	0.743584	-0.868944	4.026757
H	3.672575	-0.244005	-1.762038
H	4.022862	1.272387	-0.932616
H	4.569447	-0.252284	-0.235746
H	-5.418329	1.706667	0.491919
H	-5.493230	-0.065139	0.567958
H	-6.030842	0.920757	1.940435

50

3-methylene-4$_{\mathrm{Mes}}$, E(B3LYP/6-311+G**)=-1138.740626

C	-0.142162	0.004753	0.093805
C	-0.046672	0.000363	1.507143
C	1.245660	0.021712	2.103565
C	2.379320	0.094443	1.293502
C	2.298084	0.124035	-0.096569
C	1.030206	0.066590	-0.667368

P	-1.402816	-0.166092	2.773479
C	-3.093860	-0.462576	1.978974
C	-3.854578	0.820612	1.698546
C	-3.368111	1.972446	2.529852
B	-3.268872	1.635917	4.227175
B	-1.726687	2.333916	4.738891
B	-3.256249	3.208422	5.009094
B	-1.803262	4.081572	4.445815
B	-3.399969	4.453217	3.736259
B	-4.305960	2.932162	3.592990
B	-3.507292	3.632602	2.165077
B	-1.957228	4.344283	2.683762
B	-0.928453	3.025732	3.309829
B	-1.972523	2.776041	1.899455
C	-1.890001	1.621624	3.186343
C	1.469453	-0.033911	3.602081
C	3.537271	0.234160	-0.948967
C	-1.434656	-0.086095	-0.686774
H	-1.490334	5.271813	2.119450
H	-3.713034	3.322154	6.093112
H	-1.217849	4.838681	5.139652
H	-3.961133	5.477248	3.920146
H	-3.679931	0.598388	4.601534
H	-5.472458	2.758450	3.598992
H	-1.099251	1.748235	5.547813
H	0.236202	2.924863	3.160632
H	-1.586149	2.457205	0.838634
H	-4.137953	3.940874	1.216970
C	-4.859040	0.929740	0.832032
H	-3.034574	-1.107404	1.105568
H	3.355198	0.121211	1.768426
H	0.940802	0.071912	-1.749424
H	-2.204926	0.589933	-0.320329
H	-1.252384	0.163129	-1.733357
H	-1.840698	-1.102939	-0.669820
H	2.538980	-0.066750	3.816823
H	1.060914	0.838500	4.117681
H	1.006085	-0.916643	4.049474
H	3.373202	-0.176197	-1.947708
H	3.834016	1.281954	-1.068210
H	4.380292	-0.293541	-0.496575
H	-5.389641	1.862706	0.687950
H	-5.182004	0.078468	0.244079
H	-3.630013	-1.026625	2.748282

77

3-methyl-4$_{Mes*}$, E(B3LYP/6-311+G**)=-1492.605880

C	-1.215607	1.451509	0.018161
C	-0.749354	0.474317	0.949191
C	-1.612994	-0.643642	1.245622

C	-2.634105	-0.932128	0.339898
C	-2.916675	-0.140863	-0.774452
C	-2.257770	1.079819	-0.843651
P	0.982049	0.055944	1.533994
C	2.262397	1.354103	1.609221
C	3.344036	1.217366	0.825960
C	3.248171	0.127031	-0.202606
B	3.276638	-1.479439	0.458082
B	1.983973	-2.324124	-0.397935
B	3.688289	-2.520555	-0.887592
B	2.409288	-2.352351	-2.121584
B	3.962863	-1.489953	-2.320740
B	4.486425	-0.932260	-0.714313
B	3.713985	0.203295	-1.850198
B	2.425920	-0.671001	-2.720844
B	1.203815	-1.185160	-1.520653
B	2.013164	0.381264	-1.386164
C	1.818531	-0.659265	-0.015142
C	-1.588899	-1.486154	2.572952
C	-1.259600	-0.590423	3.792719
C	-3.982798	-0.581977	-1.790686
C	-5.359586	-0.681974	-1.092651
C	-0.901069	2.980528	-0.005305
C	-2.200697	3.673536	0.499600
C	-3.004218	-2.055233	2.862352
C	-0.636791	-2.703897	2.526377
C	-4.109350	0.400713	-2.969448
C	-3.595307	-1.967065	-2.360022
C	0.217969	3.450500	0.929003
C	-0.584762	3.511204	-1.422694
H	2.116064	-0.364425	-3.819876
H	4.274505	-3.526998	-0.685860
H	2.084936	-3.257667	-2.809528
H	4.754368	-1.776649	-3.150915
H	3.486977	-1.574023	1.612184
H	5.585454	-0.735029	-0.333163
H	1.335747	-3.103182	0.201822
H	0.036403	-1.202414	-1.673577
H	1.465474	1.416097	-1.365062
H	4.297017	1.159043	-2.223566
C	4.602809	2.027034	0.920631
H	2.241306	2.079376	2.413161
H	-3.245348	-1.804692	0.511753
H	-2.574665	1.795359	-1.587363
H	-0.786259	-3.319515	3.419472
H	-0.844430	-3.326242	1.652018
H	0.410448	-2.409147	2.494733
H	-3.007432	-2.481012	3.868772
H	-3.774511	-1.281089	2.822437
H	-3.285255	-2.860629	2.180508

H	-1.360009	-1.177880	4.710079
H	-0.243845	-0.199132	3.769784
H	-1.954724	0.251610	3.854584
H	-2.051448	4.757330	0.528468
H	-3.056478	3.467492	-0.144924
H	-2.452761	3.337929	1.509295
H	0.202961	4.543225	0.977823
H	0.080639	3.076037	1.946290
H	1.200549	3.153384	0.571894
H	-0.426422	4.592912	-1.378094
H	0.320240	3.057821	-1.829346
H	-1.395265	3.333435	-2.131272
H	-4.840405	0.018547	-3.686942
H	-4.455579	1.386730	-2.647209
H	-3.160110	0.524535	-3.497648
H	-6.124709	-0.995000	-1.809772
H	-5.350919	-1.408979	-0.277323
H	-5.658902	0.284437	-0.677302
H	-4.345294	-2.300161	-3.083876
H	-2.627900	-1.925432	-2.867184
H	-3.528863	-2.725110	-1.576410
H	4.824292	2.519212	-0.031505
H	4.513529	2.790178	1.694709
H	5.462101	1.391731	1.157561

77

3-methylene-4$_{Mes*}$, E(B3LYP/6-311+G**)=-1492.591648

C	-1.135879	1.422895	0.220794
C	-0.726207	0.309023	1.018812
C	-1.637988	-0.808319	1.119889
C	-2.625150	-0.934221	0.141165
C	-2.841830	0.015035	-0.857236
C	-2.152011	1.213241	-0.721759
P	0.956583	-0.309267	1.593560
C	2.368906	0.872850	2.075556
C	3.288438	1.219692	0.922350
C	3.215372	0.214661	-0.190689
B	3.384576	-1.433434	0.310290
B	2.140313	-2.312139	-0.588095
B	3.834471	-2.312825	-1.141757
B	2.499558	-2.137913	-2.315564
B	3.968111	-1.133606	-2.476379
B	4.512818	-0.690449	-0.843662
B	3.604271	0.478058	-1.830388
B	2.353998	-0.408140	-2.741490
B	1.227379	-1.143155	-1.564478
B	1.912454	0.467867	-1.288546
C	1.849005	-0.711542	-0.034688
C	-1.708469	-1.833787	2.311543
C	-1.352888	-1.160522	3.660055

C	-3.873811	-0.243491	-1.967091
C	-5.280662	-0.405150	-1.344724
C	-0.789705	2.928216	0.439675
C	-2.074025	3.555641	1.055358
C	-3.171761	-2.326233	2.489245
C	-0.848735	-3.101032	2.094275
C	-3.928605	0.903258	-2.993343
C	-3.493667	-1.541553	-2.717578
C	0.341701	3.205997	1.430593
C	-0.460293	3.683368	-0.869057
H	1.978138	-0.023093	-3.794150
H	4.506506	-3.280830	-1.050099
H	2.222701	-2.994466	-3.082081
H	4.744468	-1.267921	-3.357886
H	3.650746	-1.638549	1.438201
H	5.600754	-0.435247	-0.466255
H	1.585142	-3.196034	-0.041963
H	0.060341	-1.241318	-1.684831
H	1.281133	1.443234	-1.153451
H	4.082927	1.514807	-2.127045
C	4.097467	2.276339	0.887968
H	2.038866	1.747607	2.626462
H	-3.262845	-1.804637	0.162075
H	-2.422676	2.039145	-1.362019
H	-1.068249	-3.832570	2.878972
H	-1.078763	-3.565035	1.131194
H	0.217013	-2.883700	2.122041
H	-3.238253	-2.885166	3.425965
H	-3.880534	-1.496071	2.541404
H	-3.491467	-3.005704	1.696705
H	-1.528000	-1.869909	4.474191
H	-0.310670	-0.852219	3.720294
H	-1.985016	-0.285377	3.835342
H	-1.902606	4.616373	1.264054
H	-2.931063	3.476285	0.384639
H	-2.336488	3.061711	1.994842
H	0.381003	4.279743	1.635330
H	0.171017	2.697642	2.381733
H	1.310273	2.921131	1.027170
H	-0.289528	4.739867	-0.641753
H	0.442393	3.296046	-1.343699
H	-1.268898	3.639737	-1.600062
H	-4.636744	0.650079	-3.786911
H	-4.266412	1.841232	-2.543827
H	-2.955235	1.075931	-3.460340
H	-6.021715	-0.588778	-2.128822
H	-5.322388	-1.242959	-0.644964
H	-5.575620	0.498532	-0.803963
H	-4.221146	-1.746297	-3.508969
H	-2.506207	-1.453009	-3.177914

H	-3.474584	-2.406316	-2.050536
H	4.759632	2.461379	0.051260
H	4.121616	2.985687	1.707021
H	2.926724	0.250528	2.782931

13

2-methyl-1$_{H}$ E(B3LYP/6-311+G**)= -536.141177

C	1.336920	1.171514	0.008797
C	1.747254	-0.116240	0.059677
P	0.319326	-1.223519	-0.123214
C	-0.829128	0.198071	0.020932
C	-0.109655	1.345885	-0.008680
H	0.217956	-1.753120	1.194014
H	2.777625	-0.444953	0.059391
H	2.015212	2.018047	-0.018889
C	-2.323969	0.078396	0.018751
H	-0.567277	2.330390	-0.039609
H	-2.791946	1.064820	0.070490
H	-2.686882	-0.420479	-0.885701
H	-2.683104	-0.507673	0.871647

13

2-methylene-1$_{H}$ E(B3LYP/6-311+G**)= -536.126049

C	0.000000	0.000000	0.000000
C	0.000000	0.000000	1.351626
P	1.705345	0.000000	1.972287
C	2.315048	-0.311186	0.295388
C	1.320772	-0.174562	-0.617145
H	1.824702	-0.991202	2.549442
H	-0.873357	0.124073	1.977832
H	-0.893887	0.125326	-0.604106
C	3.766642	-0.479906	-0.042856
H	1.594538	0.571180	-1.333958
H	3.870251	-0.652709	-1.093715
H	4.353346	-0.387339	0.847149
H	1.315725	-1.053033	-1.228017

25

2-methyl-1$_{tBu}$ E(B3LYP/6-311+G**)= -693.441449

C	-0.035263	-0.008581	0.011168
C	0.038475	-0.009178	1.366823
C	1.386136	0.024737	1.915296
C	2.368107	0.050956	0.982333
P	1.644980	0.249249	-0.664099
C	2.127365	-1.314556	-1.655088
C	1.458256	-1.218489	-3.038047
C	3.657226	-1.273063	-1.827838
C	1.711922	-2.612899	-0.948960
H	3.425793	0.112001	1.200400
H	1.568086	0.048348	2.984908

C	-1.303250	-0.027982	-0.791635
H	2.052862	-3.477979	-1.530954
H	2.149781	-2.682646	0.049129
H	0.627275	-2.686731	-0.844794
H	1.758913	-2.074813	-3.652288
H	0.367804	-1.232320	-2.968672
H	1.752528	-0.306658	-3.564518
H	3.975704	-2.101935	-2.469981
H	3.987475	-0.342122	-2.297297
H	4.175134	-1.377466	-0.871324
H	-0.839963	-0.016980	2.006039
H	-2.177063	-0.001175	-0.134973
H	-1.366370	0.826623	-1.472000
H	-1.381431	-0.931162	-1.407758

25

2-methylene-1$_{t\mathrm{Bu}}$ E(B3LYP/6-311+G**)= -693.427611

C	-0.045172	0.041538	-0.074905
C	0.017437	-0.262833	1.417245
C	1.446536	-0.090235	1.860133
C	2.351026	0.160882	0.908507
P	1.646863	0.352747	-0.774948
C	2.122968	-1.305206	-1.631746
C	1.422885	-1.318337	-3.000947
C	3.648834	-1.263256	-1.833364
C	1.730748	-2.553468	-0.831305
H	3.402968	0.315071	1.120286
H	1.700968	-0.169395	2.913814
C	-1.178219	0.167899	-0.768411
H	2.059041	-3.455803	-1.361469
H	2.194187	-2.561801	0.158185
H	0.647379	-2.621475	-0.704162
H	1.719381	-2.211499	-3.562870
H	0.334699	-1.342371	-2.896322
H	1.689304	-0.441583	-3.598154
H	3.971128	-2.135489	-2.413461
H	3.960493	-0.367418	-2.378520
H	4.184893	-1.286416	-0.880443
H	-0.652533	0.403694	1.973275
H	-2.149705	0.008591	-0.307058
H	-1.183096	0.441071	-1.817774
H	-0.325837	-1.284847	1.630297

23

2-methyl-1$_{\mathrm{Ph}}$ E(B3LYP/6-311+G**)= -767.251633

C	-0.007002	-0.116757	0.026594
C	-0.025967	-0.036130	1.425982
C	1.185958	0.071768	2.116555
C	2.397217	0.103306	1.423059
C	2.405922	0.023099	0.033532

C	1.200907	-0.087846	-0.663635
P	-1.599778	-0.058489	2.402402
C	-2.665529	-1.293541	1.612489
C	-3.757946	-0.689415	1.090650
C	-3.718962	0.769135	1.128470
C	-2.605303	1.298334	1.690311
H	-2.518222	-2.362025	1.693181
H	-4.607705	-1.227341	0.682692
C	-2.281452	2.752600	1.848430
H	1.182657	0.128283	3.199699
H	3.329610	0.187208	1.970274
H	3.345749	0.044664	-0.506878
H	1.204414	-0.152317	-1.746298
H	-0.940558	-0.204398	-0.517864
H	-4.529794	1.374005	0.732715
H	-1.305038	2.992310	1.413125
H	-3.032936	3.374848	1.355554
H	-2.240367	3.043912	2.902952

23

2-methylene-1$_{Ph}$ E(B3LYP/6-311+G**)= -767.236870

C	-0.035416	-0.305072	0.049066
C	-0.020838	-0.073704	1.430656
C	1.204426	0.181020	2.058034
C	2.388342	0.221216	1.320817
C	2.360793	-0.005664	-0.052902
C	1.146209	-0.272045	-0.686846
P	-1.544818	-0.084025	2.500819
C	-2.643343	-1.263123	1.629728
C	-3.674134	-0.692907	0.998393
C	-3.742920	0.811480	1.013246
C	-2.553810	1.325294	1.817384
H	-2.499804	-2.335738	1.693396
H	-4.449292	-1.265810	0.496333
C	-2.298893	2.610553	2.059383
H	1.231758	0.344632	3.130433
H	3.329356	0.422369	1.820996
H	3.279947	0.019662	-0.627690
H	1.121067	-0.454384	-1.755876
H	-0.973702	-0.516665	-0.451684
H	-4.683047	1.151892	1.464919
H	-1.453515	2.925372	2.661632
H	-2.923557	3.401779	1.653120
H	-3.736952	1.211657	-0.009377

32

2-methyl-1$_{Mes}$ E(B3LYP/6-311+G**)= -885.227120

C	0.000000	0.000000	0.000000
C	0.000000	0.000000	1.398984
C	1.236611	0.000000	2.083997

C	2.444283	-0.000969	1.343123
C	2.388141	-0.001757	-0.051650
C	1.177076	-0.000838	-0.744476
C	-1.327415	-0.002921	2.112855
P	1.397237	0.005233	3.930673
C	0.382244	1.284520	4.687335
C	-0.425984	0.739922	5.638987
C	3.810133	-0.001438	1.998819
C	1.065700	0.649604	-2.101704
C	0.383594	-1.278980	4.682944
C	-0.418235	-0.719289	5.625095
H	0.534241	-2.340836	4.541507
H	-1.001798	-1.295848	6.336597
C	0.604482	2.755682	4.496828
H	3.318735	-0.002696	-0.611559
H	-0.953881	0.000400	-0.518756
H	-1.443632	-0.895693	2.732123
H	-1.421894	0.855730	2.781589
H	-2.149543	0.025971	1.395558
H	4.592824	0.000625	1.237634
H	3.957030	0.874043	2.636222
H	3.958230	-0.879319	2.632628
H	0.348455	0.832241	-2.904207
H	1.079633	1.407726	-2.891032
H	2.049030	0.947066	-2.478820
H	-0.896931	1.521993	6.197086
H	-0.130527	3.302025	5.050155
H	1.581326	3.017071	4.846616
H	0.521176	2.997378	3.457817

32

2-methylene-1$_{Mes}$ E(B3LYP/6-311+G**)= -885.209347

C	0.022245	0.300054	0.031971
C	0.042715	0.227237	1.427202
C	1.288776	0.142757	2.097011
C	2.481038	0.135612	1.331355
C	2.404501	0.211616	-0.062767
C	1.187325	0.297106	-0.733786
C	-1.285302	0.248464	2.145594
P	1.524717	0.026563	3.945091
C	0.418033	1.285939	4.737864
C	-0.540618	0.597729	5.706166
C	3.862785	0.040963	1.949766
C	1.129644	0.409924	-2.237456
C	0.433462	-1.306136	4.563128
C	-0.461083	-0.888403	5.464000
H	0.601893	-2.349471	4.321722
H	-1.099905	-1.565083	6.024657
C	0.495123	2.604640	4.555079
H	3.325954	0.201465	-0.637169

H	-0.939245	0.359208	-0.470018
H	-1.419518	-0.632100	2.776025
H	-1.370219	1.124006	2.793170
H	-2.104870	0.280160	1.425242
H	4.624933	0.087538	1.169144
H	4.052414	0.850916	2.657547
H	4.001936	-0.892660	2.500480
H	0.250319	-0.097397	-2.641943
H	1.072459	1.459123	-2.547886
H	2.017656	-0.022023	-2.704334
H	-0.256849	0.829210	6.741285
H	-0.162153	3.290635	5.082857
H	1.204410	3.049569	3.865677
H	-1.566858	0.967639	5.584965

59

2-methyl-1$_{Mes*}$ E(B3LYP/6-311+G**)=-1239.091567

C	-0.058857	-1.069589	-0.417057
C	0.403763	0.273480	-0.308663
C	-0.562900	1.335970	-0.232411
C	-1.899479	0.998444	-0.031821
C	-2.347294	-0.318653	0.093441
C	-1.417534	-1.316201	-0.151638
P	2.119319	0.757531	0.228229
C	3.553183	0.643962	-0.831162
C	4.558307	0.066157	-0.113292
C	4.150876	-0.487231	1.158473
C	2.825286	-0.340079	1.463548
C	-0.239881	2.857488	-0.398162
C	0.801141	3.097467	-1.517950
C	-3.820885	-0.603965	0.431221
C	-4.729375	-0.023287	-0.677858
C	0.724386	-2.337657	-0.883532
C	-0.092675	-3.009226	-2.025244
C	-1.498085	3.648799	-0.839828
C	0.233224	3.491654	0.932906
C	-4.115418	-2.110341	0.552688
C	-4.171911	0.063445	1.781856
C	2.113086	-2.073479	-1.481471
C	0.876160	-3.347009	0.277245
H	3.663779	1.135510	-1.786970
H	5.590436	0.041270	-0.445116
C	2.163182	-0.732772	2.753372
H	-2.627162	1.790142	0.052446
H	-1.750185	-2.342578	-0.149208
H	1.343517	-4.266921	-0.088188
H	1.510703	-2.943317	1.067885
H	-0.088063	-3.614899	0.716397
H	0.470463	-3.862216	-2.414228
H	-1.065697	-3.382228	-1.704173

H	-0.255949	-2.309329	-2.849411
H	2.479536	-2.999224	-1.935765
H	2.078536	-1.313816	-2.264836
H	2.841173	-1.773516	-0.735879
H	-1.199716	4.672419	-1.079678
H	-1.961702	3.217644	-1.731110
H	-2.253770	3.718644	-0.054476
H	0.380401	4.568471	0.798570
H	-0.521271	3.350291	1.712229
H	1.171269	3.061775	1.281444
H	0.903093	4.171871	-1.697455
H	1.792845	2.726228	-1.267009
H	0.481596	2.628240	-2.452722
H	-5.219086	-0.126127	2.037317
H	-3.548426	-0.334215	2.587448
H	-4.026977	1.145619	1.750646
H	-5.165215	-2.257326	0.820813
H	-3.941610	-2.638534	-0.388769
H	-3.507251	-2.582500	1.329172
H	-5.781112	-0.219944	-0.447418
H	-4.607612	1.057454	-0.779791
H	-4.502805	-0.478506	-1.645962
H	4.861997	-0.945817	1.838674
H	1.174584	-1.171548	2.592000
H	2.774814	-1.462337	3.290852
H	2.025292	0.132118	3.410880

59

2-methylene-1$_{Mes*}$ E(B3LYP/6-311+G**)=-1239.070440

C	-0.041108	-1.048446	-0.397269
C	0.403001	0.300195	-0.262655
C	-0.593793	1.338200	-0.196596
C	-1.923408	0.973436	0.005504
C	-2.346380	-0.352195	0.118216
C	-1.396868	-1.325586	-0.147019
P	2.081413	0.883134	0.371158
C	3.530646	0.725192	-0.730699
C	4.559976	0.118345	-0.131987
C	4.293065	-0.491321	1.223455
C	2.795556	-0.377212	1.509370
C	-0.319549	2.869518	-0.381181
C	0.789149	3.138382	-1.426990
C	-3.812626	-0.671341	0.456938
C	-4.738197	-0.095287	-0.640292
C	0.743566	-2.293008	-0.919033
C	-0.000659	-2.797619	-2.189106
C	-1.577630	3.586197	-0.940625
C	0.024361	3.557095	0.962391
C	-4.075872	-2.184855	0.560591
C	-4.171234	-0.028426	1.817395

C	2.186330	-2.036040	-1.366596
C	0.766567	-3.427719	0.131878
H	3.601355	1.228855	-1.687350
H	5.556736	0.075276	-0.560868
C	2.167715	-0.993258	2.512422
H	-2.665734	1.751153	0.096346
H	-1.711818	-2.357628	-0.177711
H	1.278301	-4.303071	-0.280840
H	1.300986	-3.114322	1.030504
H	-0.236638	-3.742649	0.427129
H	0.533258	-3.656892	-2.606065
H	-1.025765	-3.110635	-1.988030
H	-0.033296	-2.016348	-2.953772
H	2.559183	-2.926442	-1.882276
H	2.255974	-1.197907	-2.061373
H	2.850999	-1.846743	-0.530959
H	-1.308374	4.612904	-1.201352
H	-1.958164	3.098854	-1.842289
H	-2.389926	3.651991	-0.214162
H	0.138830	4.635697	0.809545
H	-0.781170	3.404644	1.686597
H	0.948441	3.170785	1.389711
H	0.830229	4.209338	-1.646903
H	1.780038	2.853003	-1.078798
H	0.581596	2.610404	-2.362039
H	-5.213839	-0.240486	2.074258
H	-3.537318	-0.424828	2.615438
H	-4.045899	1.056507	1.799615
H	-5.120507	-2.356968	0.834210
H	-3.898302	-2.697193	-0.388945
H	-3.451878	-2.654332	1.326037
H	-5.784755	-0.316565	-0.408186
H	-4.638959	0.988955	-0.728515
H	-4.506518	-0.532862	-1.615333
H	4.855852	0.051650	1.994714
H	1.106942	-0.862235	2.693879
H	2.697845	-1.662276	3.185039
H	4.635454	-1.532111	1.276708

15

2-methyl-3$_H$ E(B3LYP/6-311+G**)= -537.363240

C	-0.012377	-0.057769	0.004847
P	0.030637	-0.246144	1.884334
C	1.868084	-0.023583	1.864035
C	2.372760	-0.176646	0.634993
C	1.407416	-0.423984	-0.497120
C	2.656342	0.174657	3.125597
H	1.673840	0.150834	-1.390845
H	-0.779120	-0.698624	-0.431862
H	-0.332549	1.084815	2.253729

H	-0.252284	0.975460	-0.250868
H	1.447428	-1.480154	-0.794833
H	3.444106	-0.172524	0.445198
H	2.372087	1.105377	3.628979
H	2.473946	-0.637864	3.837026
H	3.729311	0.214827	2.920606

15

2-methylene-3$_{\mathrm{H}}$ E(B3LYP/6-311+G**)= -537.358000

C	0.000000	0.000000	0.000000
P	0.000000	0.000000	1.895662
C	1.837544	0.000000	1.878985
C	2.360753	-0.295593	0.687039
C	1.393923	-0.507427	-0.452473
C	2.654646	0.164000	3.126161
H	1.729093	-0.000077	-1.363638
H	-0.804285	-0.630721	-0.379521
H	-0.210205	1.394839	2.115066
H	-0.174216	1.015022	-0.359871
H	1.351870	-1.575823	-0.700555
H	3.126639	-1.017444	0.880044
H	2.009450	0.397911	3.947079
H	3.688420	0.008081	2.898344
H	2.987285	0.571648	0.702870

27

2-methyl-3$_{t\mathrm{Bu}}$ E(B3LYP/6-311+G**)= -694.662023

C	0.000000	0.000000	0.000000
C	0.000000	0.000000	1.546371
C	1.436025	0.000000	2.033137
H	1.620424	-0.173562	3.001745
P	1.664216	0.730678	-0.534763
C	2.355909	0.295802	1.106499
C	3.817155	0.427600	1.418574
H	-0.554676	-0.855924	1.948464
H	-0.826418	0.581621	-0.409810
C	2.305591	-0.713991	-1.515155
H	-0.085386	-1.017987	-0.383107
H	-0.507357	0.894454	1.932689
C	3.659824	-0.381444	-2.067843
C	2.410656	-1.915920	-0.623903
C	1.363530	-1.009667	-2.644357
H	1.732492	-1.840740	-3.208346
H	0.397510	-1.246884	-2.250106
H	1.288583	-0.152291	-3.280117
H	2.779618	-2.746993	-1.187892
H	3.082659	-1.705005	0.181594
H	1.444636	-2.153137	-0.229653
H	4.028787	-1.212517	-2.631832
H	3.584878	0.475932	-2.703603
H	4.331828	-0.170529	-1.262346

H	3.995443	0.120317	2.427876
H	4.381356	-0.191126	0.752423
H	4.117022	1.447624	1.298035

27

2-methylene-3$_{t\mathrm{Bu}}$ E(B3LYP/6-311+G**)= -694.657132

C	0.000000	0.000000	0.000000
C	0.000000	0.000000	1.546371
C	1.436025	0.000000	2.033137
H	1.720690	-0.684559	2.705946
P	1.664216	0.730678	-0.534763
C	2.355909	0.295802	1.106499
C	3.817155	0.427600	1.418574
H	-0.554676	-0.855924	1.948464
H	-0.826418	0.581621	-0.409810
C	2.305591	-0.713991	-1.515155
H	-0.085386	-1.017987	-0.383107
H	-0.507357	0.894454	1.932689
C	3.659824	-0.381444	-2.067843
C	2.410656	-1.915920	-0.623903
C	1.363530	-1.009667	-2.644357
H	1.732492	-1.840740	-3.208346
H	0.397510	-1.246884	-2.250106
H	1.288583	-0.152291	-3.280117
H	2.779618	-2.746993	-1.187892
H	3.082659	-1.705005	0.181594
H	1.444636	-2.153137	-0.229653
H	4.028787	-1.212517	-2.631832
H	3.584878	0.475932	-2.703603
H	4.331828	-0.170529	-1.262346
H	4.304700	0.956710	0.626587
H	4.024514	-0.038833	2.358969
H	1.698242	0.468971	2.958452

25

2-methyl-3$_{\mathrm{Ph}}$ E(B3LYP/6-311+G**)= -768.474990

C	-0.152112	-0.224360	0.259961
P	0.285293	0.062783	2.075021
C	2.076438	0.136699	1.626079
C	2.305287	-0.353117	0.401336
C	1.116483	-0.807289	-0.407698
C	3.122285	0.566753	2.613238
H	1.195776	-0.501297	-1.456786
H	-1.011240	-0.890321	0.169884
C	-0.149506	1.853680	2.322984
H	-0.420539	0.732664	-0.192414
H	1.074237	-1.904338	-0.415939
C	-1.071508	2.173187	3.326807
C	-1.449551	3.496881	3.555533
C	-0.900351	4.520491	2.787378

C	0.026332	4.216373	1.788803
C	0.396821	2.893894	1.558324
H	-1.494144	1.378998	3.933584
H	-2.168193	3.726489	4.334646
H	-1.188692	5.550480	2.965940
H	0.460588	5.010986	1.191479
H	1.120760	2.667801	0.781905
H	3.311897	-0.482494	0.008371
H	2.966671	1.602590	2.931839
H	3.083869	-0.050883	3.517030
H	4.126094	0.487445	2.187696

25

2-methylene-3$_{Ph}$ E(B3LYP/6-311+G**)= -768.467289

C	0.000000	0.000000	0.000000
P	0.000000	0.000000	1.895662
C	1.837544	0.000000	1.878985
C	2.360753	-0.295593	0.687039
C	1.393923	-0.507427	-0.452473
C	2.654646	0.164000	3.126161
H	1.729093	-0.000077	-1.363638
H	-0.804285	-0.630721	-0.379521
C	-0.273882	1.817379	2.181531
H	-0.174216	1.015022	-0.359871
H	1.351870	-1.575823	-0.700555
C	-0.349188	2.317077	3.487146
C	-0.555336	3.684997	3.702316
C	-0.686178	4.553219	2.611871
C	-0.610873	4.053521	1.306256
C	-0.404725	2.685601	1.091086
H	-0.247411	1.641724	4.335357
H	-0.613913	4.073690	4.717898
H	-0.846532	5.617265	2.779243
H	-0.712650	4.728874	0.458045
H	-0.346148	2.296908	0.075504
H	2.905460	-1.124862	1.087674
H	2.009450	0.397911	3.947079
H	3.688420	0.008081	2.898344
H	3.151064	0.304036	0.286098

34

2-methyl-3$_{Mes}$ E(B3LYP/6-311+G**)= -886.445174

C	0.000000	0.000000	0.000000
C	0.000000	0.000000	1.415616
C	1.248912	0.000000	2.094200
C	2.436292	-0.000021	1.360858
C	2.447186	-0.007525	-0.033867
C	1.219861	-0.009889	-0.688246
P	-1.505240	-0.015411	2.526555
C	-2.686977	-1.254417	1.838514

C	-2.341728	-2.710536	1.730241
C	1.367114	0.001732	3.606559
C	3.748545	-0.016015	-0.797904
C	-1.252441	0.034686	-0.848674
C	-2.624456	1.388331	1.954895
C	-4.043619	0.773550	1.845860
C	-3.892496	-0.712335	1.626469
H	-4.626392	1.244285	1.045970
H	-2.590101	2.209098	2.672388
H	-2.291043	1.776726	0.992576
H	-4.604893	0.953127	2.772318
H	3.380830	0.001509	1.897233
H	1.201021	-0.018508	-1.774351
H	2.419454	0.019869	3.898037
H	0.877023	0.868799	4.055490
H	0.905398	-0.881100	4.054391
H	3.701387	0.635516	-1.672747
H	4.540711	0.486168	-0.233823
H	3.997639	-1.024477	-1.142757
H	-2.066037	-0.552947	-0.428508
H	-1.620871	1.059553	-0.965722
H	-1.037243	-0.341719	-1.851037
H	-4.758924	-1.311714	1.354125
H	-1.525590	-2.872140	1.017829
H	-2.001203	-3.105381	2.693520
H	-3.202371	-3.300378	1.403936

34

2-methylene-3$_{Mes}$ E(B3LYP/6-311+G**)= -886.438281

C	-2.080022	0.546538	-1.568949
P	-1.269398	0.947642	0.082477
C	-2.336244	-0.284623	0.963609
C	-3.413465	-0.810564	0.029158
C	-3.513119	0.142989	-1.176247
C	-2.257955	-0.561051	2.267412
H	-4.043770	-0.318346	-2.013994
H	-2.063721	1.424086	-2.217456
C	0.474680	0.290682	-0.003392
H	-1.566927	-0.264071	-2.091326
H	-4.078525	1.036324	-0.890184
C	1.491484	1.271299	0.120877
C	2.834354	0.885456	0.072066
C	3.218273	-0.443334	-0.087886
C	2.210006	-1.397328	-0.213193
C	0.853100	-1.064128	-0.176191
C	1.197837	2.748816	0.295696
H	3.599971	1.650234	0.162357
C	4.673690	-0.841516	-0.099857
H	2.484338	-2.439641	-0.348403
C	-0.137065	-2.196605	-0.318385

H	2.131352	3.305345	0.402124
H	0.657911	3.163699	-0.559642
H	0.581459	2.941619	1.176211
H	4.845455	-1.706434	-0.745224
H	5.308143	-0.023813	-0.449447
H	5.012464	-1.112463	0.906315
H	-0.723874	-2.329595	0.592771
H	-0.841780	-2.028205	-1.134831
H	0.386538	-3.131838	-0.524904
H	-3.121683	-1.807004	-0.324609
H	-1.479118	-0.145480	2.898300
H	-2.970018	-1.224990	2.750445
H	-4.370570	-0.923838	0.546032

61

2-methyl-3$_{Mes*}$ E(B3LYP/6-311+G**)=-1240.306518

C	0.048915	-1.019569	-0.442273
C	-0.380741	0.331648	-0.277182
C	0.633082	1.350871	-0.210716
C	1.959207	0.966818	-0.023219
C	2.361521	-0.365611	0.089102
C	1.396948	-1.323137	-0.183018
P	-2.018918	0.891955	0.484694
C	-2.725769	-0.499697	1.419019
C	-4.024130	-0.699779	1.171732
C	-4.648088	0.110483	0.059764
C	-3.535083	0.925725	-0.656152
C	0.376923	2.886702	-0.360328
C	0.165501	3.558689	1.017511
C	3.821021	-0.709066	0.432658
C	4.186735	-0.071657	1.793805
C	-0.769315	-2.232552	-0.987536
C	-1.017680	-3.288910	0.112914
C	1.598983	3.574498	-1.026122
C	-0.815414	3.198490	-1.293425
C	4.058175	-2.226797	0.538008
C	4.759816	-0.149904	-0.662058
C	-2.107878	-1.858048	-1.642359
C	0.057554	-2.900132	-2.125227
C	-1.982181	-1.166497	2.538151
H	-5.197996	-0.532397	-0.637770
H	-3.836585	1.965234	-0.788479
H	-3.306485	0.526163	-1.641685
H	-5.393559	0.792757	0.486203
H	2.712709	1.734644	0.067006
H	1.693497	-2.360633	-0.209985
H	-0.538453	-3.695272	-2.582614
H	0.307283	-2.174199	-2.903823
H	0.986643	-3.352367	-1.777638
H	-2.513381	-2.736844	-2.152617

H	-2.846336	-1.540042	-0.916582
H	-1.972503	-1.073896	-2.391294
H	-1.517983	-4.164955	-0.312873
H	-0.080215	-3.626489	0.562152
H	-1.652169	-2.894739	0.907239
H	5.098741	-2.416694	0.815457
H	3.423419	-2.685008	1.301467
H	3.875063	-2.736284	-0.412011
H	5.224580	-0.301908	2.054546
H	4.080528	1.015299	1.774643
H	3.542789	-0.455843	2.589781
H	5.802031	-0.387250	-0.425983
H	4.524441	-0.585438	-1.637142
H	4.677884	0.935612	-0.752527
H	1.338376	4.611061	-1.255892
H	2.476307	3.609758	-0.377953
H	1.881248	3.082283	-1.960762
H	0.042382	4.639954	0.891938
H	-0.716933	3.166402	1.522037
H	1.032988	3.392366	1.662563
H	-0.838847	4.271032	-1.507956
H	-0.722863	2.666726	-2.244466
H	-1.776901	2.949411	-0.849708
H	-4.618405	-1.398033	1.723257
H	-2.580924	-1.954797	2.944340
H	-1.064625	-1.571419	2.165286
H	-1.770569	-0.448924	3.303141

61

2-methylene-3$_{Mes*}$ E(B3LYP/6-311+G**)=-1240.298138

C	0.135638	-1.069034	-0.424005
C	-0.367870	0.260953	-0.297138
C	0.590194	1.335382	-0.221250
C	1.929774	1.022927	0.002043
C	2.402751	-0.284029	0.129019
C	1.495840	-1.293669	-0.148855
P	-2.065161	0.771335	0.351120
C	-2.718656	-0.538992	1.467203
C	-4.205646	-0.732675	1.193227
C	-4.691476	0.392283	0.256990
C	-3.576415	0.683306	-0.768072
C	0.265522	2.856567	-0.403337
C	-0.080709	3.529047	0.946966
C	3.873490	-0.544703	0.496307
C	4.181929	0.117698	1.859731
C	-0.590066	-2.345822	-0.952636
C	-0.632019	-3.460768	0.117917
C	1.492923	3.610557	-0.982398
C	-0.867095	3.093569	-1.428704
C	4.192684	-2.046476	0.612742

C	4.796370	0.061631	-0.586760
C	-2.013678	-2.125132	-1.473189
C	0.215772	-2.861203	-2.180310
C	-2.062063	-1.107947	2.481944
H	-5.625555	0.121660	-0.242953
H	-3.726260	1.646224	-1.258549
H	-3.535634	-0.083893	-1.537833
H	-4.890986	1.294890	0.843316
H	2.638982	1.829922	0.102970
H	1.849599	-2.313351	-0.167492
H	-0.295432	-3.726556	-2.613058
H	0.285167	-2.088459	-2.951090
H	1.229724	-3.171482	-1.926630
H	-2.366588	-3.046471	-1.946056
H	-2.709096	-1.883545	-0.677013
H	-2.041012	-1.335273	-2.226405
H	-1.086997	-4.363023	-0.303633
H	0.366211	-3.729490	0.470612
H	-1.223055	-3.151179	0.981157
H	5.238062	-2.177096	0.905779
H	3.573477	-2.535927	1.369521
H	4.051891	-2.569608	-0.337096
H	5.226877	-0.053269	2.137025
H	4.015719	1.196968	1.833830
H	3.548616	-0.298829	2.647917
H	5.846306	-0.116610	-0.333703
H	4.600736	-0.390010	-1.563301
H	4.655741	1.140556	-0.683471
H	1.188285	4.627465	-1.242943
H	2.313430	3.704728	-0.268648
H	1.875561	3.131273	-1.887542
H	-0.239907	4.602710	0.798877
H	-0.981319	3.103924	1.387936
H	0.742084	3.406827	1.657301
H	-0.940619	4.162507	-1.649848
H	-0.665412	2.568523	-2.366662
H	-1.841390	2.781771	-1.060147
H	-4.778173	-0.761627	2.124391
H	-2.565975	-1.774018	3.177273
H	-1.004462	-0.938690	2.651641
H	-4.354337	-1.702368	0.703103

31

2-methyl-4$_H$ E(B3LYP/6-311+G**)= -789.670573

C	0.000000	0.000000	0.000000
B	0.000000	0.000000	1.711112
B	1.526097	0.000000	0.818583
B	1.328236	-1.104119	2.162871
B	-0.361605	-1.682665	2.153971
B	0.942654	-2.717811	1.500183

B	2.104264	-1.669263	0.652595
B	0.899266	-2.605448	-0.273462
B	-0.629467	-2.614184	0.652386
B	-1.203755	-0.924765	0.777596
B	-0.442770	-1.529888	-0.705046
C	1.194195	-0.957003	-0.598949
C	1.691883	-0.349243	-1.867054
C	1.107626	0.786643	-2.257165
P	-0.152393	1.503549	-1.114990
H	-1.388767	-3.515894	0.571783
H	1.958620	-0.937519	3.148086
H	-0.940240	-1.934242	3.153082
H	1.298059	-3.710244	2.034216
H	2.213057	0.943558	0.666785
H	3.256457	-1.844593	0.469004
H	-0.316706	0.994802	2.259968
H	-2.321200	-0.550994	0.714891
H	-0.947602	-1.506514	-1.767043
H	1.255294	-3.398230	-1.071669
H	2.428192	-0.806635	-2.365694
C	1.510541	1.518480	-3.502988
H	-1.309264	1.078684	-1.828659
H	2.246108	0.947421	-4.029990
H	0.653041	1.658548	-4.127468
H	1.919884	2.471516	-3.240192

31

2-methylene-4$_{\mathrm{H}}$ E(B3LYP/6-311+G**)= -789.665466

C	0.000000	0.000000	0.000000
B	0.000000	0.000000	1.711112
B	1.526097	0.000000	0.818583
B	1.328236	-1.104119	2.162871
B	-0.361605	-1.682665	2.153971
B	0.942654	-2.717811	1.500183
B	2.104264	-1.669263	0.652595
B	0.899266	-2.605448	-0.273462
B	-0.629467	-2.614184	0.652386
B	-1.203755	-0.924765	0.777596
B	-0.442770	-1.529888	-0.705046
C	1.194195	-0.957003	-0.598949
C	1.691883	-0.349243	-1.867054
C	1.107626	0.786643	-2.257165
P	-0.152393	1.503549	-1.114990
H	-1.388767	-3.515894	0.571783
H	1.958620	-0.937519	3.148086
H	-0.940240	-1.934242	3.153082
H	1.298059	-3.710244	2.034216
H	2.213057	0.943558	0.666785
H	3.256457	-1.844593	0.469004
H	-0.316706	0.994802	2.259968

H	-2.321200	-0.550994	0.714891
H	-0.947602	-1.506514	-1.767043
H	1.255294	-3.398230	-1.071669
H	2.627822	-0.157581	-1.571615
C	1.510541	1.518480	-3.502988
H	-1.309264	1.078684	-1.828659
H	2.246108	0.947421	-4.029990
H	0.966581	2.437569	-3.568444
H	1.711611	-1.100206	-2.629002

43

2-methyl-4$_{t\text{Bu}}$ E(B3LYP/6-311+G**)= -946.962719

C	0.000000	0.000000	0.000000
B	0.000000	0.000000	1.713945
B	1.530137	0.000000	0.831623
B	1.334499	-1.092206	2.181711
B	-0.350642	-1.680952	2.170788
B	0.963817	-2.713233	1.531790
B	2.119272	-1.662188	0.678103
B	0.927203	-2.613532	-0.241117
B	-0.603768	-2.625912	0.677561
B	-1.192625	-0.941187	0.784225
B	-0.416847	-1.550875	-0.688175
C	1.204852	-0.961743	-0.584021
C	1.697734	-0.369254	-1.860403
C	1.130394	0.780519	-2.244722
P	-0.024439	1.570044	-1.059958
C	-1.680550	1.795778	-1.988995
C	-2.695454	2.306143	-0.947640
C	-1.370309	2.929524	-2.991964
C	-2.253159	0.589934	-2.742430
H	-1.357770	-3.532582	0.597649
H	1.957919	-0.911113	3.169101
H	-0.931555	-1.926449	3.170480
H	1.323760	-3.700017	2.073744
H	2.213187	0.945945	0.682350
H	3.274024	-1.826403	0.499528
H	-0.324933	0.994087	2.260049
H	-2.318072	-0.593376	0.728846
H	-0.899187	-1.582041	-1.754649
H	1.288392	-3.409853	-1.033821
H	2.418981	-0.842788	-2.365942
C	1.552891	1.494183	-3.494593
H	-3.120732	0.915654	-3.328105
H	-1.528523	0.155422	-3.434771
H	-2.592481	-0.193034	-2.064872
H	-3.627382	2.581628	-1.452822
H	-2.935131	1.542430	-0.204536
H	-2.325437	3.190250	-0.421633
H	-2.302798	3.249802	-3.467880

H	-0.930211	3.803422	-2.503298
H	-0.694128	2.601092	-3.786002
H	2.223749	0.873061	-4.050521
H	0.690880	1.710791	-4.090343
H	2.045424	2.407779	-3.234491

43

2-methylene-4$_{tBu}$ E(B3LYP/6-311+G**)= -946.956075

C	0.055299	0.018504	-0.080063
B	-0.119026	0.018159	1.621445
B	1.490950	0.033848	0.892771
B	1.174485	-1.056367	2.228348
B	-0.493677	-1.667238	2.045172
B	0.890467	-2.684714	1.552030
B	2.112939	-1.620162	0.819077
B	1.036449	-2.585648	-0.213843
B	-0.577570	-2.618611	0.532609
B	-1.195181	-0.946478	0.579688
B	-0.255651	-1.529103	-0.806073
C	1.329686	-0.932839	-0.532062
C	2.016406	-0.380511	-1.774434
C	1.162559	0.717731	-2.407273
P	0.085021	1.579537	-1.161494
C	-1.651862	1.853926	-1.922487
C	-2.556720	2.274127	-0.745131
C	-1.495555	3.074415	-2.857522
C	-2.275300	0.685877	-2.694854
H	-1.310918	-3.532498	0.378047
H	1.689452	-0.863325	3.274225
H	-1.174736	-1.917554	2.978348
H	1.205503	-3.666004	2.130838
H	2.177754	0.986613	0.814061
H	3.282811	-1.765780	0.758194
H	-0.510985	1.006007	2.133678
H	-2.316999	-0.630226	0.412386
H	-0.630279	-1.552949	-1.918935
H	1.489206	-3.372943	-0.967928
H	2.992078	0.023638	-1.492093
C	1.316355	1.052172	-3.690196
H	-3.209543	1.021859	-3.159606
H	-1.619216	0.327046	-3.490648
H	-2.517613	-0.153836	-2.043938
H	-3.536055	2.569944	-1.136204
H	-2.716097	1.464492	-0.032578
H	-2.141728	3.129417	-0.204817
H	-2.491434	3.429815	-3.142014
H	-0.974162	3.902460	-2.369042
H	-0.966779	2.829056	-3.779761
H	2.017263	0.516118	-4.323772
H	0.777140	1.863844	-4.158548

H	2.187084	-1.186218	-2.490590

41

2-methyl-4$_{Ph}$ E(B3LYP/6-311+G**)=-1020.779376

C	0.000000	0.000000	0.000000
C	0.000000	0.000000	1.401034
C	1.226545	0.000000	2.080667
C	2.426991	0.032248	1.375386
C	2.416036	0.034403	-0.018206
C	1.202149	0.011351	-0.703476
P	-1.514776	-0.099102	2.450900
C	-2.867470	-0.427123	1.251785
C	-3.777366	0.538424	1.085686
C	-3.532452	1.821098	1.806960
B	-3.605915	1.675224	3.540711
B	-2.186711	2.558821	4.110919
B	-3.806227	3.311645	4.124561
B	-2.377681	4.254142	3.614637
B	-3.912546	4.394094	2.707042
B	-4.663806	2.782372	2.645679
B	-3.773550	3.410227	1.233305
B	-2.355990	4.320630	1.829665
B	-1.293236	3.174260	2.699311
B	-2.150179	2.693640	1.224705
C	-2.123358	1.684330	2.639418
H	-1.910210	5.226574	1.215585
H	-4.389095	3.500790	5.134937
H	-1.941338	5.130985	4.276262
H	-4.575233	5.372688	2.719721
H	-3.963645	0.638220	3.966595
H	-5.806547	2.504351	2.549452
H	-1.600580	2.120275	5.036176
H	-0.114297	3.159729	2.687548
H	-1.632973	2.293604	0.250183
H	-4.326589	3.548885	0.199892
H	-4.683747	0.440655	0.470125
C	-2.999317	-1.776411	0.609882
H	1.242070	-0.028213	3.165273
H	3.367974	0.043713	1.913455
H	3.349867	0.048141	-0.568731
H	1.190162	0.007979	-1.787673
H	-0.937091	-0.005334	-0.543440
H	-3.839186	-1.775454	-0.053079
H	-2.109598	-1.997342	0.058077
H	-3.143216	-2.518927	1.366759

41

2-methylene-4$_{Ph}$ E(B3LYP/6-311+G**)=-1020.772967

C	0.000000	0.000000	0.000000
C	0.000000	0.000000	1.401034

C	1.226545	0.000000	2.080667
C	2.426991	0.032248	1.375386
C	2.416036	0.034403	-0.018206
C	1.202149	0.011351	-0.703476
P	-1.514776	-0.099102	2.450900
C	-2.867470	-0.427123	1.251785
C	-3.777366	0.538424	1.085686
C	-3.532452	1.821098	1.806960
B	-3.605915	1.675224	3.540711
B	-2.186711	2.558821	4.110919
B	-3.806227	3.311645	4.124561
B	-2.377681	4.254142	3.614637
B	-3.912546	4.394094	2.707042
B	-4.663806	2.782372	2.645679
B	-3.773550	3.410227	1.233305
B	-2.355990	4.320630	1.829665
B	-1.293236	3.174260	2.699311
B	-2.150179	2.693640	1.224705
C	-2.123358	1.684330	2.639418
H	-1.910210	5.226574	1.215585
H	-4.389095	3.500790	5.134937
H	-1.941338	5.130985	4.276262
H	-4.575233	5.372688	2.719721
H	-3.963645	0.638220	3.966595
H	-5.806547	2.504351	2.549452
H	-1.600580	2.120275	5.036176
H	-0.114297	3.159729	2.687548
H	-1.632973	2.293604	0.250183
H	-4.326589	3.548885	0.199892
H	-4.781826	0.132506	1.276185
C	-2.999317	-1.776411	0.609882
H	1.242070	-0.028213	3.165273
H	3.367974	0.043713	1.913455
H	3.349867	0.048141	-0.568731
H	1.190162	0.007979	-1.787673
H	-0.937091	-0.005334	-0.543440
H	-3.839186	-1.775454	-0.053079
H	-2.222147	-2.419028	0.967583
H	-4.156931	0.754963	0.108987

50

2-methyl-4$_{Mes}$ E(B3LYP/6-311+G**)=-1138.747881

C	0.000000	0.000000	0.000000
C	0.000000	0.000000	1.416720
C	1.240847	0.000000	2.107083
C	2.431507	0.055894	1.380759
C	2.449074	0.090097	-0.011011
C	1.224198	0.047584	-0.673484
P	-1.472630	-0.132934	2.544644
C	-3.026172	-0.207791	1.575265

C	-3.881282	0.809327	1.725042
C	-3.402199	1.973976	2.520632
B	-3.193056	1.623893	4.211588
B	-1.632213	2.342392	4.612168
B	-3.156149	3.188199	4.995161
B	-1.761937	4.093824	4.344544
B	-3.411620	4.438895	3.744910
B	-4.290874	2.895702	3.644558
B	-3.604284	3.633931	2.172594
B	-2.038179	4.375620	2.602449
B	-0.942948	3.068207	3.138815
B	-2.070713	2.823369	1.798188
C	-1.887754	1.646423	3.064513
C	1.343473	-0.052857	3.616457
C	3.746292	0.184325	-0.774194
C	-1.240739	-0.088765	-0.860714
H	-1.628031	5.319385	2.021182
H	-3.540822	3.289662	6.107980
H	-1.146879	4.853983	5.008797
H	-3.980073	5.447729	3.982158
H	-3.558523	0.568379	4.581338
H	-5.452509	2.702848	3.722524
H	-0.938822	1.765311	5.372032
H	0.211588	2.988500	2.914588
H	-1.753365	2.512801	0.711307
H	-4.311749	3.929720	1.275266
H	-4.810041	0.826447	1.354754
C	-3.399238	-1.460227	0.838906
H	3.371039	0.063825	1.924582
H	1.214196	0.049102	-1.759070
H	-1.983224	0.665870	-0.605311
H	-0.974182	0.045566	-1.910259
H	-1.720887	-1.067842	-0.773532
H	2.379436	-0.218204	3.916800
H	1.014466	0.879638	4.082396
H	0.735997	-0.856992	4.039490
H	3.665938	-0.282914	-1.758219
H	4.028580	1.231314	-0.930099
H	4.563608	-0.297022	-0.232384
H	-4.331789	-1.313470	0.335209
H	-2.639410	-1.692338	0.122187
H	-3.492636	-2.268279	1.534053

50

2-methylene-4$_{\mathrm{Mes}}$ E(B3LYP/6-311+G**)=-1138.741920

C	0.000000	0.000000	0.000000
C	0.000000	0.000000	1.416720
C	1.240847	0.000000	2.107083
C	2.431507	0.055894	1.380759
C	2.449074	0.090097	-0.011011

C	1.224198	0.047584	-0.673484
P	-1.472630	-0.132934	2.544644
C	-3.026172	-0.207791	1.575265
C	-3.881282	0.809327	1.725042
C	-3.402199	1.973976	2.520632
B	-3.193056	1.623893	4.211588
B	-1.632213	2.342392	4.612168
B	-3.156149	3.188199	4.995161
B	-1.761937	4.093824	4.344544
B	-3.411620	4.438895	3.744910
B	-4.290874	2.895702	3.644558
B	-3.604284	3.633931	2.172594
B	-2.038179	4.375620	2.602449
B	-0.942948	3.068207	3.138815
B	-2.070713	2.823369	1.798188
C	-1.887754	1.646423	3.064513
C	1.343473	-0.052857	3.616457
C	3.746292	0.184325	-0.774194
C	-1.240739	-0.088765	-0.860714
H	-1.628031	5.319385	2.021182
H	-3.540822	3.289662	6.107980
H	-1.146879	4.853983	5.008797
H	-3.980073	5.447729	3.982158
H	-3.558523	0.568379	4.581338
H	-5.452509	2.702848	3.722524
H	-0.938822	1.765311	5.372032
H	0.211588	2.988500	2.914588
H	-1.753365	2.512801	0.711307
H	-4.311749	3.929720	1.275266
H	-4.759249	0.460378	2.052775
C	-3.399238	-1.460227	0.838906
H	3.371039	0.063825	1.924582
H	1.214196	0.049102	-1.759070
H	-1.983224	0.665870	-0.605311
H	-0.974182	0.045566	-1.910259
H	-1.720887	-1.067842	-0.773532
H	2.379436	-0.218204	3.916800
H	1.014466	0.879638	4.082396
H	0.735997	-0.856992	4.039490
H	3.665938	-0.282914	-1.758219
H	4.028580	1.231314	-0.930099
H	4.563608	-0.297022	-0.232384
H	-4.331789	-1.313470	0.335209
H	-2.644101	-2.202588	0.992424
H	-4.124577	1.139062	0.736619

77

2-methyl-4$_{Mes*}$ E(B3LYP/6-311+G**)=-1492.601074

C	-1.165370	0.224532	-1.374096
C	-0.686961	0.953315	-0.241099

C	-1.550498	1.053680	0.912039
C	-2.601294	0.142768	1.021179
C	-2.913371	-0.789765	0.031823
C	-2.240914	-0.653092	-1.175263
P	1.054069	1.369536	0.321276
C	2.383853	1.939697	-0.834139
C	3.449868	1.125031	-0.889535
C	3.366175	-0.191603	-0.201580
B	3.311435	-0.068156	1.535525
B	2.028863	-1.184074	2.005122
B	3.747797	-1.654367	2.122081
B	2.534824	-2.814691	1.516561
B	4.128338	-2.680037	0.710860
B	4.592570	-0.961225	0.705410
B	3.916914	-1.713106	-0.762849
B	2.642400	-2.859573	-0.264043
B	1.344656	-1.920584	0.535305
B	2.204482	-1.280951	-0.869812
C	1.907705	-0.305458	0.531893
C	-1.486430	2.177228	2.009564
C	-1.049876	3.535546	1.407989
C	-4.014528	-1.835862	0.269861
C	-5.366108	-1.125169	0.516505
C	-0.813243	0.441688	-2.877524
C	-2.064937	1.127607	-3.499943
C	-2.908573	2.434907	2.579499
C	-0.594353	1.805793	3.217708
C	-4.182977	-2.791216	-0.925890
C	-3.645302	-2.680803	1.511985
C	0.359188	1.385885	-3.149289
C	-0.562873	-0.881430	-3.639065
H	2.395705	-3.818380	-0.910440
H	4.287437	-1.756881	3.168815
H	2.210720	-3.762419	2.144990
H	4.947276	-3.531148	0.764170
H	3.461973	1.005361	1.993024
H	5.675267	-0.491607	0.684034
H	1.320855	-0.875732	2.895857
H	0.186034	-2.113026	0.448954
H	1.701420	-0.957191	-1.874357
H	4.549451	-1.738987	-1.759221
H	4.390858	1.407111	-1.349593
C	2.418691	3.365562	-1.316908
H	-3.210725	0.162724	1.911538
H	-2.568329	-1.243874	-2.017021
H	-0.706187	2.560620	4.003026
H	-0.892563	0.840659	3.636171
H	0.459209	1.749963	2.950094
H	-2.876579	3.333408	3.200692
H	-3.643854	2.598916	1.787777

H	-3.263412	1.625062	3.219901
H	-1.135250	4.313065	2.172679
H	-0.016891	3.537243	1.064062
H	-1.695294	3.814943	0.570478
H	-1.880344	1.337529	-4.558096
H	-2.955368	0.500838	-3.430162
H	-2.280603	2.074726	-2.997885
H	0.432853	1.563381	-4.226078
H	0.193489	2.351918	-2.671783
H	1.313324	0.984933	-2.817172
H	-0.335409	-0.657706	-4.685494
H	0.278267	-1.437408	-3.225122
H	-1.432768	-1.540249	-3.634755
H	-4.942499	-3.541320	-0.689901
H	-4.512943	-2.266863	-1.827071
H	-3.254702	-3.321056	-1.155836
H	-6.155282	-1.864047	0.686206
H	-5.329610	-0.471452	1.390939
H	-5.651350	-0.516269	-0.345994
H	-4.419232	-3.430861	1.701193
H	-2.695836	-3.201644	1.363141
H	-3.551170	-2.065138	2.409355
H	3.276525	3.531796	-1.972440
H	1.516677	3.650951	-1.859350
H	2.509185	4.045847	-0.463558

77

2-methylene-4$_{Mes*}$ E(B3LYP/6-311+G**)=-1492.593422

C	-1.160215	0.601740	-1.278929
C	-0.670964	1.013270	-0.000271
C	-1.521613	0.794787	1.146327
C	-2.546816	-0.144833	1.025518
C	-2.852509	-0.797943	-0.168104
C	-2.209254	-0.327905	-1.306097
P	1.068667	1.330181	0.650387
C	2.389133	2.119116	-0.401357
C	3.416399	1.175570	-1.021939
C	3.281506	-0.219422	-0.424838
B	3.444397	-0.295983	1.288928
B	2.179986	-1.416117	1.815630
B	3.865905	-1.966209	1.637420
B	2.515806	-2.992486	1.075076
B	3.991624	-2.833543	0.080622
B	4.558653	-1.153550	0.210686
B	3.649992	-1.679291	-1.224525
B	2.381011	-2.815636	-0.698499
B	1.262767	-1.932587	0.381881
B	1.970627	-1.145413	-1.037168
C	1.908875	-0.358032	0.491506
C	-1.483894	1.613182	2.489733

C	-1.100064	3.092837	2.242286
C	-3.923641	-1.900455	-0.199728
C	-5.290203	-1.313768	0.225661
C	-0.862963	1.248251	-2.666645
C	-2.135429	2.075070	-3.012391
C	-2.909203	1.670921	3.105753
C	-0.568904	0.991159	3.570217
C	-4.080490	-2.523746	-1.599041
C	-3.520227	-3.024185	0.783835
C	0.307095	2.232095	-2.698833
C	-0.640323	0.210602	-3.791913
H	1.992463	-3.673969	-1.412585
H	4.529711	-2.216781	2.582707
H	2.219931	-3.995776	1.626329
H	4.755288	-3.721308	-0.082314
H	3.720943	0.703339	1.847249
H	5.654580	-0.729446	0.097408
H	1.624206	-1.191223	2.829546
H	0.093478	-2.061882	0.429606
H	1.353748	-0.676218	-1.915220
H	4.137135	-1.606998	-2.297783
H	4.425564	1.552359	-0.844314
C	2.550294	3.443437	-0.408200
H	-3.142722	-0.372120	1.896099
H	-2.544356	-0.679934	-2.269869
H	-0.707748	1.520955	4.518417
H	-0.821581	-0.059934	3.734180
H	0.483825	1.050607	3.300949
H	-2.902582	2.389126	3.929358
H	-3.657099	1.998445	2.379354
H	-3.229733	0.715985	3.526825
H	-1.211233	3.654783	3.174182
H	-0.069066	3.214311	1.915000
H	-1.758280	3.545836	1.495540
H	-1.996289	2.581289	-3.972811
H	-3.024834	1.446976	-3.085643
H	-2.323382	2.836357	-2.250520
H	0.325644	2.733256	-3.671178
H	0.214361	3.000111	-1.931731
H	1.264603	1.733287	-2.576480
H	-0.474592	0.735370	-4.737456
H	0.232860	-0.414759	-3.600575
H	-1.497306	-0.448494	-3.938500
H	-4.814570	-3.332936	-1.558458
H	-4.437892	-1.797626	-2.334538
H	-3.140142	-2.947343	-1.961903
H	-6.058459	-2.092882	0.205058
H	-5.260069	-0.903394	1.237605
H	-5.600545	-0.513323	-0.451857
H	-4.273492	-3.817902	0.778773

H	-2.560068	-3.464631	0.502864
H	-3.431300	-2.655665	1.808254
H	3.392423	3.911032	-0.910384
H	1.844208	4.111850	0.071927
H	3.278995	1.110142	-2.104450

32

3-methyl-6, E(B3LYP/6-311+G**)=-827.776332

C	-0.195293	-0.000058	0.054685
C	-0.035742	-0.000049	1.483424
C	1.121941	0.000005	2.185950
C	2.466554	0.000068	1.525621
B	2.947827	1.450979	0.695008
B	3.866243	0.888961	-0.717376
B	3.866328	-0.888702	-0.717371
B	5.290306	0.000200	-0.138048
B	4.712713	1.446678	0.739205
B	3.794875	0.891858	2.152570
B	3.794961	-0.891590	2.152575
B	5.242185	0.000202	1.644416
B	4.712852	-1.446329	0.739212
B	2.947967	-1.450801	0.695015
C	2.521377	0.000067	-0.148633
P	0.963086	-0.000010	-1.169356
H	6.212472	0.000250	2.319331
H	2.181534	-2.343946	0.677714
H	2.181309	2.344051	0.677702
H	5.288243	2.478494	0.756321
H	3.635186	1.491531	3.154204
H	3.725181	1.483274	-1.727366
H	3.725323	-1.483034	-1.727358
H	5.288482	-2.478090	0.756334
H	3.635330	-1.491272	3.154213
H	6.296810	0.000247	-0.757257
H	-0.953094	-0.000091	2.064270
H	-1.221581	-0.000106	-0.304627
C	1.100529	0.000000	3.688832
H	0.072773	-0.000038	4.052678
H	1.612697	-0.877567	4.092563
H	1.612635	0.877601	4.092568

32

3-methylene-6, E(B3LYP/6-311+G**)=-827.757174

C	-0.040043	-0.081043	-0.250237
P	-0.396996	0.328160	1.328844
C	1.280802	0.143128	2.143285
B	2.333173	1.509842	2.116872
B	3.284453	1.382184	3.596328
B	4.208081	-0.148023	3.534511
B	3.965054	0.808486	2.058653

B	3.824834	-0.965057	2.003679
B	3.044352	-1.503600	3.509843
B	2.716674	-0.051596	4.503052
B	1.560253	0.964918	3.619176
B	1.410765	-0.803364	3.563693
B	2.098060	-1.376351	2.030692
C	2.673160	0.049520	1.229255
C	2.516886	0.102567	-0.269477
C	3.469951	0.617412	-1.045293
C	1.262146	-0.489139	-0.883166
H	5.285018	-0.248598	4.011154
H	1.997240	2.450725	1.495905
H	1.609279	-2.221009	1.374403
H	3.267687	-2.570575	3.965858
H	4.532906	-1.597133	1.304110
H	0.430114	-1.329739	3.955327
H	0.674647	1.618532	4.044656
H	3.689358	2.365376	4.111566
H	4.789082	1.367153	1.429696
H	2.710655	-0.087242	5.684231
H	1.246617	-0.227090	-1.943556
H	-0.899199	-0.068624	-0.918210
H	3.349019	0.637122	-2.122233
H	4.389779	1.021530	-0.646224
H	1.325152	-1.586901	-0.848617

10

TS_1$_{\mathrm{H}}$, E(B3LYP/6-311+G**)=-496.783943

C	-0.023766	0.068371	-0.035843
C	0.017975	-0.001055	1.351592
C	1.316918	-0.000420	1.925282
C	2.370391	0.069443	1.021489
P	1.626914	0.132282	-0.533335
H	3.423989	0.082909	1.247942
H	1.481726	-0.050695	2.993877
H	-0.882820	-0.052224	1.949548
H	-0.900608	0.080016	-0.662339
H	2.188936	0.209480	-1.803866

22

TS_1$_{t\mathrm{Bu}}$, E(B3LYP/6-311+G**)=-654.089625

C	0.023279	0.003273	-0.009284
P	-0.001622	0.000212	1.716043
C	1.704027	-0.005253	1.980057
C	2.284541	-0.004679	0.711580
C	1.371357	-0.000045	-0.369118
H	2.223068	-0.008481	2.924981
H	3.358105	-0.007583	0.570783
H	1.689871	0.000884	-1.404056
H	-0.819803	0.006967	-0.681603

C	-1.424211	0.002283	2.927874
C	-0.809955	-0.001208	4.339947
C	-2.279501	1.267678	2.721383
C	-2.286108	-1.258053	2.718006
H	-3.122324	-1.248459	3.426295
H	-2.703172	-1.296510	1.708910
H	-1.707180	-2.169281	2.879607
H	-3.115871	1.260455	3.429522
H	-1.695869	2.175426	2.885631
H	-2.696220	1.311150	1.712347
H	-1.619825	-0.000289	5.076357
H	-0.198790	-0.889846	4.513783
H	-0.194469	0.883942	4.516278

20

TS_1$_{Ph}$, E(B3LYP/6-311+G**)=-727.899554

C	-0.010500	0.367550	-0.053746
C	0.071243	0.152564	1.319728
C	1.365486	-0.158071	1.810004
C	2.372122	-0.204345	0.848820
P	1.594523	0.157086	-0.646787
H	3.417334	-0.406816	1.017408
H	1.563828	-0.340919	2.858326
H	-0.797243	0.213160	1.963010
H	-0.898332	0.598715	-0.619610
C	2.250584	0.276751	-2.302818
C	3.462425	-0.352196	-2.632054
C	3.974482	-0.243724	-3.920785
C	3.279979	0.464503	-4.901101
C	2.069807	1.078674	-4.579827
C	1.559559	1.000685	-3.288177
H	3.997223	-0.929795	-1.887187
H	4.913679	-0.728286	-4.162502
H	3.678213	0.537139	-5.906277
H	1.525227	1.635211	-5.334161
H	0.632915	1.506811	-3.043997

29

TS_1$_{Mes}$, E(B3LYP/6-311+G**)=-845.879518

C	-0.062166	0.143057	0.023360
C	-0.070262	-0.054004	1.425345
C	1.146510	-0.219833	2.130261
C	2.343233	-0.173759	1.414460
C	2.378471	0.014976	0.032512
C	1.165213	0.163434	-0.640013
P	-1.634536	-0.099338	2.315300
C	-2.140052	0.690709	3.766822
C	-3.454355	0.289141	3.984381
C	-3.979930	-0.604640	3.016905
C	-3.106089	-0.944768	1.988942

H	-1.563685	1.367084	4.377033
H	-4.031300	0.639205	4.831030
H	-4.986696	-0.999168	3.071663
H	-3.308043	-1.610604	1.165594
C	1.195336	-0.443492	3.621781
H	3.275755	-0.302911	1.955262
C	3.690850	0.084440	-0.708127
H	1.170225	0.309441	-1.715807
C	-1.328433	0.323942	-0.777226
H	2.186370	-0.785741	3.924761
H	0.980911	0.475998	4.175010
H	0.457562	-1.184622	3.940088
H	-1.098993	0.699112	-1.776061
H	-1.869419	-0.619879	-0.895884
H	-2.013284	1.023232	-0.290547
H	4.060050	1.115174	-0.750021
H	4.458418	-0.517490	-0.216305
H	3.587952	-0.267914	-1.736915

56

TS_1$_{Mes*}$, E(B3LYP/6-311+G**)=-1199.755846

C	-1.672431	-1.155243	-0.003583
C	-2.376869	0.044138	0.083518
C	-1.645619	1.221975	-0.002756
C	-0.248650	1.257448	-0.108116
C	0.444329	0.006704	-0.097522
C	-0.282762	-1.230218	-0.107992
C	-3.908003	0.022519	0.239891
C	-4.277146	-0.742384	1.532952
C	0.409392	2.667282	-0.270824
C	1.263569	3.062008	0.959030
P	2.251352	-0.017532	0.117635
C	3.145229	-0.022565	1.593548
C	4.492490	-0.040820	1.238665
C	4.741711	-0.048710	-0.155292
C	3.602818	-0.037316	-0.958780
C	0.340323	-2.655071	-0.272952
C	1.177124	-2.731026	-1.573512
C	1.253923	2.723905	-1.567461
C	-0.651608	3.785593	-0.419428
C	1.190474	-3.070793	0.952750
C	-0.748354	-3.746862	-0.416386
C	-4.512298	1.435758	0.332298
C	-4.540542	-0.692419	-0.977461
H	3.576099	-0.040563	-2.036977
H	5.740579	-0.062786	-0.572983
H	5.285567	-0.048615	1.975757
H	2.739365	-0.014419	2.592979
H	-2.239792	-2.070839	0.013580
H	-2.182574	2.153227	0.015809

H	1.504983	-4.112750	0.838154
H	2.086131	-2.466173	1.075617
H	0.600546	-2.998708	1.871501
H	1.528094	-3.756620	-1.722445
H	0.567824	-2.455424	-2.439416
H	2.053994	-2.088404	-1.549076
H	-0.251922	-4.705174	-0.585122
H	-1.356355	-3.856178	0.485431
H	-1.410448	-3.568303	-1.267627
H	1.628630	3.741325	-1.714586
H	2.115814	2.061533	-1.539313
H	0.642500	2.462396	-2.436252
H	1.603141	4.096322	0.846697
H	0.667922	3.003362	1.875058
H	2.143978	2.436069	1.085301
H	-0.130725	4.730855	-0.588106
H	-1.315307	3.622070	-1.272417
H	-1.259921	3.911709	0.479886
H	-5.363808	-0.767556	1.659150
H	-3.920451	-1.774541	1.512500
H	-3.844512	-0.256009	2.411581
H	-5.630113	-0.713793	-0.878216
H	-4.294604	-0.172149	-1.907302
H	-4.194806	-1.724421	-1.069697
H	-5.596011	1.361284	0.455822
H	-4.121194	1.991701	1.188764
H	-4.325515	2.021138	-0.572099

16

TS_2$_{H}$, E(B3LYP/6-311+G**)=-650.464441

C	-0.019941	-0.000614	-0.002174
C	-0.020927	-0.000174	1.372069
C	1.276067	0.000057	1.991035
C	1.558802	0.000414	3.372787
C	2.865541	0.000554	3.819942
P	1.645662	-0.000797	-0.509202
C	2.381094	-0.000151	1.075864
C	3.706574	-0.000078	1.541290
C	3.938531	0.000265	2.904142
H	-0.884387	-0.000612	-0.647383
H	-0.935098	0.000003	1.952443
H	2.209605	-0.000960	-1.779837
H	0.735295	0.000603	4.079557
H	3.072065	0.000845	4.884059
H	4.536784	-0.000307	0.844402
H	4.958007	0.000326	3.272947

28

TS_2$_{t\text{Bu}}$, E(B3LYP/6-311+G**)=-807.769342

C	0.142581	-0.925529	0.145908

C	0.079112	-0.546315	1.504606
C	1.170996	0.027147	2.125062
C	2.370135	0.244656	1.412595
C	2.418887	-0.147131	0.029194
C	1.296197	-0.730994	-0.589240
C	3.581005	0.824644	1.911416
C	4.605559	0.919072	0.993646
P	4.027936	0.266685	-0.514360
H	5.588226	1.324787	1.178797
H	3.680862	1.163177	2.935358
C	4.870936	0.076796	-2.174384
H	1.118514	0.317414	3.169767
H	-0.836442	-0.708376	2.062126
H	1.330256	-1.027210	-1.631822
H	-0.724080	-1.374696	-0.326275
C	4.104856	0.879284	-3.244105
C	6.296516	0.634964	-2.010342
C	4.924288	-1.412871	-2.565820
H	5.408663	-1.514530	-3.543746
H	5.489990	-1.996072	-1.837087
H	3.924436	-1.846364	-2.642647
H	6.822132	0.540108	-2.965801
H	6.289178	1.692192	-1.735139
H	6.864748	0.082133	-1.258668
H	4.597929	0.753826	-4.214895
H	3.073703	0.533380	-3.347059
H	4.081442	1.943423	-3.002755

26

TS_2$_{Ph}$, E(B3LYP/6-311+G**)=-881.579954

C	0.036506	-0.387798	0.002361
C	0.030261	-0.246154	1.405887
C	1.204464	-0.007308	2.093712
C	2.429673	0.095111	1.403619
C	2.419087	-0.059287	-0.024968
C	1.215721	-0.296003	-0.713710
C	3.724086	0.349467	1.969774
C	4.760321	0.406706	1.065229
P	4.084665	0.134545	-0.514070
H	5.800953	0.573734	1.296052
H	3.871534	0.477716	3.034877
C	4.860021	0.072695	-2.117528
H	1.195048	0.106488	3.173019
H	-0.906201	-0.323508	1.946345
H	1.206163	-0.399934	-1.792429
H	-0.894763	-0.569206	-0.522293
C	4.269008	-0.652885	-3.166807
C	4.875501	-0.685596	-4.418120
C	6.086774	-0.030020	-4.636715
C	6.683844	0.676753	-3.592615

C	6.073168	0.746109	-2.345366
H	3.348850	-1.200315	-3.001699
H	4.406413	-1.243076	-5.221016
H	6.560648	-0.069059	-5.610362
H	7.622917	1.194163	-3.753566
H	6.530179	1.329074	-1.554349

35

TS_2$_{Mes}$, E(B3LYP/6-311+G**)=-999.560476

C	-0.003482	0.067557	-0.006727
C	0.042471	0.048106	1.387157
C	1.308492	0.002713	2.021859
C	2.495873	-0.017684	1.249662
C	2.386865	0.027788	-0.141062
C	1.152250	0.062781	-0.789767
C	-1.249959	0.075992	2.164864
P	1.413872	-0.020720	3.817366
C	0.742614	-1.109569	5.000735
C	1.139917	-0.676330	6.245626
C	1.955274	0.504038	6.246672
C	2.226679	1.042694	4.942794
C	3.865379	-0.093567	1.877338
C	1.066240	0.065290	-2.295795
C	2.995881	2.211182	4.794932
H	0.863725	-1.187819	7.159524
H	0.137832	-1.978515	4.791228
H	3.296463	0.020814	-0.733592
H	-0.972913	0.103962	-0.494197
H	3.902645	-0.868660	2.647705
H	4.621576	-0.315158	1.122243
H	4.147430	0.845230	2.363104
H	-2.083618	0.346840	1.514557
H	-1.477912	-0.899823	2.604864
H	-1.201250	0.791466	2.989995
H	1.954022	0.514773	-2.745874
H	0.983704	-0.956691	-2.682035
H	0.189891	0.617000	-2.643963
C	3.503118	2.832971	5.921164
H	3.181351	2.628994	3.811878
H	4.096554	3.734131	5.813769
C	3.253109	2.311717	7.208349
C	2.490567	1.170628	7.368376
H	3.660129	2.816073	8.077396
H	2.294071	0.776821	8.360672

62

TS_2$_{Mes*}$, E(B3LYP/6-311+G**)=-1353.436936

C	-0.961512	-1.229217	-0.078550
C	-2.348663	-1.161880	0.059496
C	-3.063526	0.032888	0.112659

C	-2.336697	1.215172	0.062762
C	-0.942879	1.260781	-0.075075
C	-0.249517	0.013501	-0.186644
C	-4.597245	0.001663	0.239141
C	-5.194064	-0.751971	-0.972967
C	-0.286957	2.681154	-0.071892
C	-1.323796	3.798638	0.201945
P	1.524537	0.001635	-0.594833
C	2.289503	0.001369	-2.159379
C	3.654388	-0.008699	-1.973712
C	4.073656	-0.015578	-0.603396
C	2.996633	-0.011004	0.349367
C	-0.322905	-2.656981	-0.079040
C	-1.374487	-3.761865	0.188746
C	-4.990852	-0.731066	1.543556
C	-5.215841	1.411207	0.276865
C	0.759443	2.803929	1.061923
C	0.343787	3.025380	-1.443727
C	0.306551	-3.004676	-1.450564
C	0.719307	-2.795881	1.056667
H	1.774888	0.007561	-3.108590
H	4.360188	-0.011308	-2.795262
C	3.263243	-0.016387	1.731675
H	-2.906643	-2.080634	0.127299
H	-2.876160	2.142366	0.130980
H	1.083951	-3.826815	1.093055
H	1.584481	-2.151814	0.922537
H	0.264411	-2.568142	2.025328
H	0.636077	-4.048227	-1.444737
H	-0.430263	-2.891131	-2.251540
H	1.169743	-2.387755	-1.688742
H	-0.858541	-4.724155	0.219429
H	-1.880974	-3.633865	1.148949
H	-2.127741	-3.827607	-0.600645
H	0.685631	4.064946	-1.435561
H	1.199039	2.398793	-1.685326
H	-0.395933	2.922976	-2.243537
H	1.135713	3.830564	1.101175
H	0.304160	2.577941	2.030812
H	1.616894	2.150542	0.923634
H	-0.794908	4.753787	0.235805
H	-2.077506	3.877783	-0.585688
H	-1.830739	3.673468	1.162245
H	-6.079923	-0.759253	1.646159
H	-4.628700	-1.761409	1.558349
H	-4.580740	-0.219032	2.418411
H	-6.285264	-0.782965	-0.897651
H	-4.932586	-0.253394	-1.910412
H	-4.835192	-1.781957	-1.030346
H	-6.302972	1.329859	0.358899

H	-4.864965	1.989516	1.135886
H	-4.996619	1.977952	-0.632147
C	4.575548	-0.026525	2.167276
H	2.450518	-0.012644	2.449252
H	4.785934	-0.030727	3.231081
C	5.643724	-0.031397	1.244897
C	5.396755	-0.026008	-0.114375
H	6.664779	-0.039375	1.609174
H	6.220310	-0.029663	-0.821718

12

TS_3$_{H}$, E(B3LYP/6-311+G**)=-497.972117

C	-0.026801	0.064290	-0.159827
P	0.003675	0.143110	1.688902
C	1.758533	0.004804	1.896718
C	2.331468	-0.265705	0.713259
C	1.383972	-0.529338	-0.451698
H	2.279199	0.152456	2.832482
H	3.401590	-0.371226	0.585326
H	1.771013	-0.116663	-1.386762
H	-0.820369	-0.572298	-0.545946
H	-1.007061	0.253804	2.633487
H	-0.134362	1.064048	-0.586290
H	1.296437	-1.616073	-0.591034

24

TS_3$_{t\mathrm{Bu}}$, E(B3LYP/6-311+G**)=-655.271584

C	0.007292	-0.044340	0.029437
C	-0.002814	-0.009329	1.584908
C	1.422115	0.001186	2.125551
C	2.368976	0.433238	1.273160
P	1.673750	0.692404	-0.339204
H	3.413486	0.568662	1.518787
H	1.623515	-0.248264	3.159951
H	-0.579019	-0.853715	1.972427
H	-0.819160	0.528133	-0.389321
C	2.393683	1.482067	-1.885194
H	-0.053577	-1.070551	-0.344326
H	-0.510760	0.907811	1.919086
C	3.849382	1.845317	-1.540396
C	2.363953	0.485175	-3.060038
C	1.611909	2.754705	-2.265754
H	4.311775	2.317040	-2.413894
H	3.903229	2.553277	-0.709902
H	4.436844	0.960182	-1.285590
H	2.037602	3.195425	-3.176177
H	0.560628	2.532421	-2.466209
H	1.653540	3.495463	-1.465816
H	2.771639	0.959945	-3.961109
H	2.953809	-0.405158	-2.836318

H	1.343332	0.166595	-3.288552

22

TS_3$_{Ph}$, E(B3LYP/6-311+G**)=-729.087547

C	0.147555	-0.396160	-0.112975
C	0.057349	-0.122605	1.420492
C	1.395489	0.362828	1.962294
C	2.274402	0.830881	1.058603
P	1.639856	0.587774	-0.572824
C	2.218474	1.133378	-2.154003
C	1.563356	0.722323	-3.334390
C	2.018679	1.151127	-4.575518
C	3.133197	1.983621	-4.683294
C	3.787339	2.388906	-3.518195
C	3.342041	1.980131	-2.267738
H	3.239906	1.259607	1.288069
H	1.579986	0.381081	3.029204
H	-0.280881	-1.023317	1.938702
H	-0.750881	-0.069286	-0.634687
H	0.303256	-1.457930	-0.320207
H	-0.697893	0.653296	1.605951
H	3.861168	2.321482	-1.379408
H	4.653858	3.038464	-3.581037
H	3.484638	2.310753	-5.654272
H	1.497069	0.824406	-5.468760
H	0.701483	0.066523	-3.280763

31

TS_3$_{Mes}$, E(B3LYP/6-311+G**)=-847.066864

C	-0.030248	-0.066538	-0.056380
C	0.039286	0.133258	1.485263
C	1.488051	0.179004	1.949650
C	2.403338	0.465888	1.008644
P	1.637597	0.545026	-0.589822
C	2.248713	1.062839	-2.191872
C	1.786331	0.409342	-3.363871
C	2.237975	0.851941	-4.606230
C	3.159056	1.892014	-4.741387
C	3.624479	2.499031	-3.573943
C	3.183643	2.124618	-2.305023
H	3.462297	0.590125	1.184674
H	1.735959	0.049090	2.995894
H	-0.521368	-0.657082	1.990917
H	-0.843748	0.504361	-0.500001
H	-0.161385	-1.120421	-0.315253
H	-0.443286	1.085367	1.747939
C	3.706338	2.855579	-1.096188
H	4.345094	3.308369	-3.649631
C	3.615330	2.360062	-6.101683
H	1.874744	0.347195	-5.496947

C	0.846997	-0.767194	-3.292679
H	0.748695	-1.244577	-4.269346
H	1.218102	-1.510905	-2.578581
H	-0.158827	-0.481451	-2.966149
H	4.221527	3.772004	-1.389878
H	2.888664	3.114441	-0.415010
H	4.418088	2.249680	-0.524969
H	4.635053	2.751613	-6.068276
H	3.589498	1.547425	-6.832064
H	2.970585	3.160813	-6.482048

58

TS_3$_{Mes*}$, E(B3LYP/6-311+G**)=-1200.934328

C	-1.666717	1.202854	-0.053993
C	-0.297173	1.269443	-0.262163
C	0.443925	0.032278	-0.259096
C	-0.267128	-1.205912	-0.287211
C	-1.639793	-1.167589	0.032631
C	-2.356895	0.004597	0.187621
C	0.340861	2.662466	-0.515759
C	1.166717	2.620240	-1.822944
P	2.203682	-0.064663	0.120712
C	2.941577	-0.992479	1.545788
C	4.284142	-0.246836	1.760585
C	4.711655	0.419677	0.461408
C	3.732997	0.656048	-0.431090
C	0.266390	-2.625839	-0.675039
C	0.394569	-3.513389	0.584793
C	-3.851251	0.039673	0.549764
C	-4.045852	0.873444	1.838370
C	-0.763944	-3.292852	-1.634040
C	1.596557	-2.664211	-1.460329
C	-4.651759	0.688839	-0.603898
C	-4.428227	-1.365582	0.800523
C	-0.723332	3.762820	-0.733668
C	1.199960	3.131099	0.684868
H	3.871946	1.170896	-1.370422
H	5.736162	0.736409	0.309094
H	5.045693	-0.937796	2.130969
H	2.305111	-0.964330	2.429164
H	3.122057	-2.040027	1.280193
H	4.153993	0.528850	2.529453
H	-2.230667	2.122647	-0.037259
H	-2.160974	-2.106397	0.135025
H	-0.377254	-4.261416	-1.961858
H	-1.733211	-3.476593	-1.171098
H	-0.925804	-2.675472	-2.521743
H	0.720407	-4.521535	0.308317
H	1.121763	-3.107382	1.290247
H	-0.561593	-3.602178	1.107186

H	1.729562	-3.676302	-1.855680
H	1.582153	-1.974293	-2.307302
H	2.472378	-2.431530	-0.859330
H	-5.105786	0.913442	2.108770
H	-3.497788	0.431092	2.674921
H	-3.694414	1.900342	1.715097
H	-5.482447	-1.284481	1.079459
H	-4.373446	-1.995042	-0.091919
H	-3.909604	-1.878347	1.615338
H	-5.717556	0.720413	-0.356216
H	-4.325776	1.712937	-0.799638
H	-4.534271	0.117815	-1.529082
H	-0.213494	4.692827	-0.999272
H	-1.410236	3.518291	-1.548183
H	-1.307820	3.963943	0.167605
H	1.673486	3.576972	-1.982635
H	1.921846	1.838810	-1.825916
H	0.509317	2.438259	-2.678286
H	1.611028	4.124319	0.477088
H	0.579792	3.204163	1.583018
H	2.026538	2.457899	0.901482

28

TS_4$_{\mathbf{H}}$, E(B3LYP/6-311+G**)=-750.278764

C	-0.000951	0.011884	0.013514
B	0.013682	0.007831	1.722661
B	1.549115	-0.006331	0.847196
B	1.328411	-1.120374	2.170117
B	-0.368729	-1.673433	2.156691
B	0.935490	-2.721830	1.497216
B	2.099960	-1.670090	0.658267
B	0.884510	-2.593802	-0.276838
B	-0.633099	-2.611367	0.661092
B	-1.209168	-0.921687	0.782064
B	-0.458430	-1.532044	-0.697035
C	1.197556	-0.937799	-0.606027
C	1.726603	-0.355225	-1.869285
C	1.170014	0.801899	-2.289130
P	-0.091844	1.355260	-1.195754
H	-1.397365	-3.508731	0.576832
H	1.957096	-0.958865	3.157606
H	-0.948958	-1.921323	3.155992
H	1.287573	-3.719030	2.024753
H	2.229906	0.941487	0.706319
H	3.249833	-1.854792	0.467605
H	-0.304683	0.996653	2.282369
H	-2.323681	-0.537671	0.729188
H	-0.969050	-1.490239	-1.754956
H	1.238903	-3.383147	-1.079342
H	2.520270	-0.864983	-2.397000

H	1.455168	1.334003	-3.185444
H	-0.934676	2.455329	-1.187482

40

TS_4$_{t\text{Bu}}$, E(B3LYP/6-311+G**)= -907.585551

C	0.028414	0.084085	-0.027355
B	0.018968	0.080245	1.686808
B	1.562060	0.025503	0.833765
B	1.296661	-1.077039	2.159235
B	-0.412678	-1.587010	2.124942
B	0.874844	-2.671368	1.488100
B	2.075263	-1.650936	0.661535
B	0.850069	-2.548799	-0.286682
B	-0.678611	-2.524580	0.630507
B	-1.211717	-0.821642	0.734345
B	-0.457736	-1.454665	-0.729424
C	1.210778	-0.901803	-0.621554
C	1.770095	-0.340123	-1.876132
C	1.239744	0.831958	-2.300709
P	-0.025597	1.439511	-1.240924
H	-1.464953	-3.402712	0.539842
H	1.914838	-0.925897	3.155571
H	-1.014004	-1.814432	3.117261
H	1.194328	-3.674782	2.025416
H	2.272237	0.953692	0.704053
H	3.223110	-1.866869	0.490577
H	-0.283498	1.075716	2.245053
H	-2.318576	-0.415672	0.670054
H	-0.955413	-1.411633	-1.793932
H	1.195211	-3.352979	-1.078881
H	2.557919	-0.869419	-2.392909
H	1.555779	1.347324	-3.197081
C	-1.117980	2.973632	-1.282162
C	-0.693795	3.734218	-2.551902
C	-2.599214	2.564385	-1.367347
C	-0.868775	3.832567	-0.029595
H	-1.301450	4.641397	-2.625415
H	0.354996	4.037587	-2.515304
H	-0.862815	3.145550	-3.456461
H	-1.512027	4.718924	-0.070938
H	-1.109619	3.289165	0.886256
H	0.170388	4.159491	0.029933
H	-3.218427	3.468355	-1.390232
H	-2.801202	1.981615	-2.267269
H	-2.904680	1.973759	-0.501365

38

TS_4$_{\text{Ph}}$, E(B3LYP/6-311+G**)= -981.394575

C	0.016034	0.107203	-0.059762
B	-0.280666	-0.028984	1.621794
B	1.387002	0.020631	1.040773
B	0.944117	-1.190425	2.213687
B	-0.715817	-1.737646	1.851970
B	0.698402	-2.735878	1.362503
B	1.982635	-1.625109	0.829764
B	0.966751	-2.472900	-0.376820
B	-0.694294	-2.558731	0.266936
B	-1.300684	-0.882867	0.408543
B	-0.290342	-1.378733	-0.952132
C	1.316435	-0.797902	-0.519561
C	2.056285	-0.125231	-1.617927
C	1.564579	1.063530	-2.041372
P	0.130088	1.546763	-1.155324
H	-1.421068	-3.445010	-0.021341
H	1.381536	-1.106124	3.308341
H	-1.465123	-2.058631	2.707889
H	0.960676	-3.770899	1.869281
H	2.072944	0.973658	1.098845
H	3.149985	-1.798388	0.838198
H	-0.708381	0.913286	2.188503
H	-2.394531	-0.499561	0.185576
H	-0.602292	-1.257610	-2.079238
H	1.469148	-3.201125	-1.157897
H	2.933108	-0.598231	-2.036626
H	1.998938	1.653441	-2.836234
C	-0.938859	2.968473	-1.244498
C	-0.471186	4.157273	-1.832221
C	-1.315417	5.257861	-1.927113
C	-2.610061	5.207925	-1.410522
C	-3.063546	4.034990	-0.808077
C	-2.247587	2.910995	-0.735693
H	0.545358	4.227028	-2.201114
H	-0.948548	6.167116	-2.389647
H	-3.256826	6.074644	-1.474188
H	-4.068109	3.984024	-0.403869
H	-2.626261	1.998187	-0.292954

47

TS_4$_{Mes}$, E(B3LYP/6-311+G**)= -1099.375328

C	-0.153930	-0.241279	0.038959
C	-0.180278	-0.053451	1.444565
C	1.014971	0.212315	2.159554
C	2.209539	0.298153	1.443959
C	2.262936	0.114260	0.061648
C	1.074978	-0.163452	-0.616531
P	-1.748019	-0.174141	2.322559
C	-2.778789	-1.529641	2.756086
C	-3.887214	-1.159732	3.440089

C	-4.042874	0.293050	3.703732
B	-4.328914	1.281413	2.275801
B	-3.262456	2.668023	2.495152
B	-5.008219	2.771891	2.872512
B	-5.488873	1.216356	3.599645
B	-4.607521	1.035568	5.147532
B	-5.202960	2.632693	4.637213
B	-3.808561	3.537245	3.947282
B	-3.586877	2.480588	5.368538
B	-2.876048	0.982655	4.826122
B	-2.379020	2.487777	4.050898
C	-2.768367	1.132468	3.077898
H	-5.745188	3.379373	2.175654
H	-3.312857	2.881643	6.446461
H	-3.701394	4.712219	4.024068
H	-6.094211	3.161013	5.206668
H	-2.132487	0.228177	5.336784
H	-5.039754	0.330001	5.989451
H	-1.249879	2.822439	4.122726
H	-2.705809	3.109545	1.552545
H	-4.454990	0.704696	1.258722
H	-6.497493	0.627685	3.427394
H	-4.639880	-1.849406	3.794715
H	-2.536767	-2.551704	2.498196
C	1.044044	0.406062	3.654022
H	3.127012	0.503122	1.986244
C	3.567123	0.239818	-0.684109
H	1.102080	-0.313755	-1.690973
C	-1.393207	-0.533819	-0.770300
H	2.069405	0.366653	4.025567
H	0.622883	1.373639	3.940294
H	0.456023	-0.360285	4.164439
H	-1.182943	-0.440228	-1.837083
H	-1.759792	-1.549214	-0.591889
H	-2.207092	0.147131	-0.512660
H	3.730382	1.275884	-1.000427
H	4.414235	-0.047880	-0.057754
H	3.575943	-0.381832	-1.581869

74

TS_4$_{Mes*}$, E(B3LYP/6-311+G**)= -1453.245272

C	1.640671	1.261238	-0.047352
C	3.040264	1.211343	-0.088445
C	3.768573	0.029629	-0.083944
C	3.048316	-1.162410	-0.088522
C	1.655214	-1.229698	-0.045955
C	0.938364	0.014515	0.029924
C	5.306641	-0.003108	-0.097277
C	5.793157	-0.724675	-1.376400
C	1.030208	-2.659679	-0.100091

C	2.084847	-3.746458	-0.425019
P	-0.827488	0.005950	0.522614
C	-1.366947	0.008114	2.193592
C	-2.709248	0.001161	2.348954
C	-3.504429	-0.006237	1.099443
B	-3.402421	-1.479273	0.145155
B	-3.242194	-0.907986	-1.514620
B	-4.816961	-1.460967	-0.877392
B	-4.933761	-0.909045	0.810066
B	-4.942805	0.880780	0.805658
B	-5.785465	-0.021502	-0.473952
B	-4.724064	-0.019691	-1.921818
B	-4.831462	1.425609	-0.884472
B	-3.417263	1.463022	0.137963
B	-3.251081	0.885351	-1.518958
C	-2.516773	-0.004659	-0.235659
C	1.003479	2.686208	-0.103729
C	2.047611	3.781639	-0.434502
C	5.927173	1.405875	-0.075623
C	5.810749	-0.769584	1.148491
C	0.414429	-3.066268	1.261112
C	-0.012180	-2.747248	-1.233059
C	-0.042863	2.762808	-1.233933
C	0.388113	3.090572	1.258329
H	-5.282420	-2.491665	-1.222174
H	-5.307175	2.449926	-1.234241
H	-5.136727	-0.024508	-3.029852
H	-6.965880	-0.027612	-0.540284
H	-2.806470	2.356202	0.596181
H	-5.408843	1.468324	1.717365
H	-2.561740	1.465789	-2.276830
H	-2.547435	-1.485174	-2.269942
H	-2.782461	-2.363914	0.607604
H	-5.393994	-1.496773	1.724600
H	-3.217514	0.000732	3.302655
H	-0.661542	0.013867	3.014182
H	3.582432	2.138937	-0.120192
H	3.605838	-2.083289	-0.122280
H	0.081953	-4.107673	1.214015
H	1.160996	-2.989738	2.057345
H	-0.443127	-2.456909	1.534748
H	1.565997	-4.701518	-0.534381
H	2.610039	-3.550664	-1.363346
H	2.822021	-3.875132	0.371549
H	-0.424027	-3.759151	-1.284770
H	-0.845063	-2.068301	-1.088420
H	0.447161	-2.519322	-2.199305
H	1.519378	4.731256	-0.546447
H	2.785248	3.920385	0.359830
H	2.573087	3.587340	-1.372958

H	-0.461932	3.771651	-1.287216
H	0.415398	2.535598	-2.200854
H	-0.870379	2.078342	-1.085014
H	0.047355	4.129243	1.210127
H	-0.463816	2.474784	1.535167
H	1.137297	3.021537	2.052801
H	6.904322	-0.801252	1.153234
H	5.481876	-0.279589	2.069097
H	5.448461	-1.799732	1.169146
H	7.017179	1.323548	-0.074928
H	5.644172	1.991842	-0.954230
H	5.639746	1.965296	0.818854
H	6.886632	-0.753215	-1.399109
H	5.432675	-1.754449	-1.426842
H	5.448767	-0.204241	-2.274251

46

TS_5$_{H}$, E(B3LYP/6-311+G**)= -1003.768347

C	0.001368	-0.039545	0.033761
B	0.013899	-0.007754	1.756872
B	1.735703	0.004781	2.198179
B	1.179954	-1.002094	0.842834
B	1.623242	-0.168330	-0.639258
B	2.357803	1.390327	-0.261270
B	2.708174	-0.069058	0.709077
B	2.470143	1.493733	1.515350
B	0.826801	1.535233	2.183765
B	-0.306487	1.477154	0.873346
B	1.188966	2.386993	0.654880
C	0.663832	1.323772	-0.604211
C	-0.004156	1.763206	-1.934429
B	0.261973	0.727121	-3.349987
B	-1.313755	0.638308	-4.122949
B	-1.718447	2.276997	-4.680208
B	-2.479794	1.632663	-3.208911
B	-1.906946	3.333574	-3.260100
B	-0.343377	3.395025	-2.396604
B	0.825408	2.398362	-3.312827
B	-0.025638	1.729282	-4.734868
B	-0.389431	3.374821	-4.179076
P	-1.324066	-0.529937	-1.123621
C	-1.224139	0.766649	-2.406663
B	-1.667700	2.372623	-1.837340
H	-2.186186	-1.610806	-1.047757
H	-0.062674	4.326442	-4.797446
H	-3.587528	1.260480	-3.052791
H	0.939229	-0.214752	-3.256103
H	-1.641874	-0.398640	-4.577968
H	1.980125	2.600343	-3.210937
H	0.559228	1.478398	-5.729674

H	-2.191879	2.455359	-0.801482
H	-2.670365	4.232454	-3.197981
H	-2.353822	2.435540	-5.663194
H	0.040414	4.254418	-1.690420
H	0.519463	2.134077	3.154116
H	3.749161	-0.619976	0.622554
H	3.363983	2.072316	2.026255
H	2.099610	-0.494024	3.205088
H	1.750757	-0.748563	-1.640099
H	1.056399	-2.173600	0.795144
H	1.118994	3.544873	0.457484
H	-1.380544	1.921583	0.814222
H	-0.889304	-0.514496	2.320261
H	3.058744	1.890824	-1.062978

58

TS_5$_{t\text{Bu}}$, E(B3LYP/6-311+G**)=-1161.074691

C	-1.414882	0.232688	-0.000007
P	-0.038367	1.453914	-0.000095
C	1.414329	0.320909	-0.000029
B	1.604333	-0.628879	1.473723
B	1.846624	-2.276875	0.894117
B	3.388639	-2.294601	0.000039
B	3.215935	-1.270982	-1.438262
B	1.846624	-2.276929	-0.894037
B	1.604331	-0.628970	-1.473740
B	2.893202	0.407036	-0.891055
B	2.893210	0.407087	0.890981
B	3.215935	-1.270899	1.438280
B	4.036658	-0.620322	-0.000011
B	-2.897125	0.237429	0.890554
B	-1.551337	-0.723496	1.474161
C	-0.731261	-1.258285	0.000000
C	0.816382	-1.210892	0.000005
B	-2.897122	0.237435	-0.890553
B	-3.123718	-1.455896	-1.438635
B	-3.237260	-2.487799	-0.000002
B	-1.698697	-2.382519	0.894112
B	-3.123715	-1.455898	1.438632
B	-3.980030	-0.853369	-0.000001
B	-1.551342	-0.723498	-1.474167
B	-1.698698	-2.382518	-0.894117
C	-0.137214	3.358543	0.000003
H	4.071271	-3.258690	0.000068
H	3.118036	1.395739	1.492654
H	0.961766	-0.330773	-2.397543
H	3.118014	1.395657	-1.492792
H	1.340595	-3.160742	-1.483604
H	3.751459	-1.478912	-2.470553
H	0.961773	-0.330629	2.397513

H	3.751460	-1.478771	2.470582
H	5.191787	-0.371474	-0.000018
H	1.340598	-3.160653	1.483737
H	-3.646602	-1.694617	2.470722
H	-3.646607	-1.694618	-2.470724
H	-3.863850	-3.489233	-0.000000
H	-5.147537	-0.671175	-0.000001
H	-0.926515	-0.388999	-2.397743
H	-3.177486	1.213885	-1.489458
H	-1.143604	-3.236789	1.483065
H	-0.926510	-0.389004	2.397740
H	-3.177506	1.213860	1.489474
H	-1.143606	-3.236787	-1.483073
C	-0.871751	3.832773	1.264193
C	1.318139	3.851630	0.001088
C	-0.869943	3.833096	-1.265124
H	1.297648	4.946097	0.000927
H	1.862271	3.527314	0.889508
H	1.863682	3.527132	-0.886408
H	-0.931664	4.927128	1.246103
H	-1.891349	3.445770	1.310984
H	-0.345404	3.528020	2.169360
H	-0.929817	4.927454	-1.246916
H	-0.342328	3.528489	-2.169605
H	-1.889486	3.446137	-1.313439

56

TS_5$_{Ph}$, E(B3LYP/6-311+G**)= -1234.883263

C	-0.064973	1.416542	0.040345
B	-0.003135	2.865315	0.976470
B	-1.063824	4.008822	0.123311
B	-0.011004	2.920089	-0.805403
B	-1.019241	1.624435	-1.426115
B	-2.669997	1.805246	-0.833422
B	-1.699365	3.216873	-1.338299
B	-2.721439	3.318507	0.108091
B	-1.688491	3.128390	1.538024
B	-1.008179	1.533812	1.523059
B	-2.663403	1.750241	0.954086
C	-1.575661	0.775011	0.024309
C	-1.575639	-0.775046	-0.024311
B	-1.008130	-1.533833	-1.523059
B	-0.003051	-2.865307	-0.976466
B	-1.063711	-4.008843	-0.123309
B	-0.010924	-2.920080	0.805407
B	-1.699278	-3.216911	1.338297
B	-2.669948	-1.805311	0.833416
B	-2.663349	-1.750307	-0.954092
B	-1.688397	-3.128430	-1.538025
B	-2.721345	-3.318575	-0.108096

P	1.097864	0.000013	0.000002
C	-0.064931	-1.416534	-0.040342
B	-1.019199	-1.624453	1.426115
C	2.894164	0.000034	0.000002
H	-3.701951	-3.976730	-0.132024
H	0.974132	-3.182016	1.398540
H	-0.688512	-0.869412	-2.423901
H	0.987102	-3.088336	-1.576114
H	-3.532741	-1.204458	-1.529518
H	-1.907703	-3.626503	-2.586442
H	-0.706521	-1.016338	2.368319
H	-1.925302	-3.778602	2.352634
H	-0.845701	-5.169492	-0.157904
H	-3.543710	-1.296369	1.435381
H	-1.907815	3.626457	2.586440
H	-1.925400	3.778560	-2.352636
H	-3.702063	3.976635	0.132017
H	-0.845847	5.169477	0.157908
H	-0.706542	1.016330	-2.368319
H	0.974047	3.182054	-1.398532
H	-3.532782	1.204367	1.529509
H	-0.688546	0.869400	2.423902
H	0.987010	3.088371	1.576121
H	-3.543743	1.296280	-1.435390
C	3.598275	-0.659234	1.021355
C	4.989433	-0.679157	0.998849
C	5.686090	0.000056	-0.000001
C	4.989420	0.679259	-0.998850
C	3.598262	0.659314	-1.021352
H	3.059015	-1.143083	1.825346
H	5.528552	-1.202749	1.779818
H	6.769940	0.000065	-0.000002
H	5.528529	1.202860	-1.779819
H	3.058993	1.143155	-1.825342

TS_5$_{Mes}$, E(B3LYP/6-311+G**)= -1352.865378

C	5.249572	-0.013798	0.000029
C	4.535596	-0.014205	1.199561
C	3.141068	-0.009322	1.232922
C	2.440732	-0.006140	0.000016
C	3.141090	-0.009470	-1.232900
C	4.535594	-0.014372	-1.199528
C	2.444928	-0.010828	2.571029
P	0.636934	-0.002815	0.000006
C	-0.531499	1.412199	-0.000007
B	-1.482535	1.581437	-1.474021
B	-3.134362	1.786282	-0.894206
B	-3.184172	3.328074	-0.000060
B	-2.157341	3.178252	1.438363
B	-3.134384	1.786303	0.894123

B	-1.482570	1.581470	1.473983
B	-0.471606	2.890224	0.890778
B	-0.471584	2.890204	-0.890820
B	-2.157306	3.178219	-1.438453
B	-1.523406	4.012080	-0.000047
C	2.444941	-0.011098	-2.571003
C	6.756425	0.014272	-0.000035
C	-0.538776	-1.411871	0.000013
C	-2.048809	-0.770964	0.000006
C	-2.044805	0.779162	-0.000015
B	-0.486591	-2.890220	-0.890771
B	-1.490718	-1.576188	-1.473970
B	-3.143596	-1.772469	-0.894134
B	-2.173784	-3.169456	-1.438380
B	-3.201391	-3.313949	0.000040
B	-2.173777	-3.169424	1.438452
B	-3.143592	-1.772448	0.894178
B	-1.490711	-1.576156	1.474002
B	-0.486587	-2.890201	0.890828
B	-1.544197	-4.006560	0.000043
H	-4.162638	3.990095	-0.000079
H	0.516869	3.133023	-1.486805
H	-1.171676	0.945022	2.397974
H	0.516831	3.133059	1.486784
H	-4.008110	1.261680	1.482754
H	-2.377934	3.709416	2.470391
H	-1.171615	0.944967	-2.397987
H	-2.377874	3.709359	-2.470500
H	-1.300909	5.172560	-0.000058
H	-4.008073	1.261646	-1.482846
H	-2.397123	-3.699459	-2.470414
H	-2.397112	-3.699403	2.470499
H	-4.183284	-3.970877	0.000049
H	-1.327726	-5.168176	0.000056
H	-1.176495	-0.941339	2.397989
H	0.500568	-3.138182	1.486825
H	-4.014570	-1.243288	-1.482764
H	-1.176510	-0.941394	-2.397974
H	0.500560	-3.138215	-1.486770
H	-4.014562	-1.243253	1.482801
H	5.079518	-0.020593	-2.138366
H	5.079509	-0.020296	2.138403
H	3.177219	-0.010856	-3.380124
H	1.808099	-0.890879	-2.689056
H	1.806348	0.867228	-2.690044
H	3.177209	-0.010417	3.380147
H	1.806260	0.867458	2.689956
H	1.808162	-0.890649	2.689201
H	7.165063	-0.474341	0.886836
H	7.165019	-0.478226	-0.884792

H	7.119673	1.047801	-0.002359

92

TS_5$_{Mes*}$, E(B3LYP/6-311+G**)= -1706.727144

C	2.095181	-0.709678	1.261307
C	1.384254	-0.756055	0.021083
C	2.110201	-0.739098	-1.217070
C	3.442930	-0.332617	-1.160763
C	4.105949	-0.022876	0.027275
C	3.433304	-0.299338	1.212193
P	-0.421075	-0.388111	0.006487
C	-2.107710	-1.205466	0.003864
B	-2.719643	-2.571184	-0.878368
B	-4.160214	-3.109495	0.008135
B	-2.726488	-2.559796	0.898211
B	-3.052753	-0.933186	1.465554
B	-4.635043	-0.404432	0.882435
B	-4.363798	-2.074194	1.439180
B	-5.351195	-1.772656	-0.004891
B	-4.353096	-2.093114	-1.437635
B	-4.628578	-0.416296	-0.904839
B	-3.041987	-0.952421	-1.469226
C	-3.220684	0.024538	-0.009284
C	-2.590565	1.435792	-0.017373
B	-1.776811	1.951817	1.451712
B	-0.308593	2.723245	0.877874
B	-0.296316	2.708721	-0.903064
B	-1.710586	3.659654	-1.467224
B	-3.177782	2.799626	-0.924756
B	-1.756296	1.927724	-1.484112
C	-0.957482	1.384646	-0.006255
C	1.579605	-1.250965	-2.589067
C	2.739216	-1.552849	-3.569261
C	5.542361	0.522876	-0.011437
C	6.092311	0.830126	1.393528
C	1.553963	-1.185408	2.642461
C	2.708007	-1.482149	3.631465
C	0.649326	-0.141037	3.323197
C	0.799406	-2.519476	2.459710
C	0.843664	-2.591230	-2.376125
C	0.662083	-0.235035	-3.295457
C	6.474209	-0.515225	-0.680485
C	5.559154	1.833809	-0.833040
B	-3.190068	2.814449	0.858299
B	-1.730385	3.683249	1.406864
B	-0.800396	4.170893	-0.027954
B	-2.595400	4.231497	-0.040946
H	-3.217051	5.236253	-0.053348
H	0.709002	2.528527	-1.489865
H	-1.798372	1.264035	2.387621

H	0.688702	2.553675	1.481112
H	-4.203943	2.696724	1.443764
H	-1.719542	4.263884	2.435935
H	-1.763056	1.224745	-2.408813
H	-1.685548	4.223133	-2.505539
H	-0.120002	5.137256	-0.031160
H	-4.183513	2.672126	-1.522089
H	-4.776455	-2.481302	-2.470043
H	-4.794927	-2.448770	2.473402
H	-6.520612	-1.940392	-0.008234
H	-4.460433	-4.252380	0.014599
H	-2.507116	-0.491638	2.394411
H	-1.971472	-3.227659	1.503462
H	-5.179810	0.433179	-1.503889
H	-2.490632	-0.522588	-2.400084
H	-1.960205	-3.246348	-1.469819
H	-5.190648	0.452821	1.466178
H	3.992919	-0.254541	-2.085168
H	3.965425	-0.192985	2.142003
H	0.373124	-0.618961	-4.278661
H	-0.243484	-0.053092	-2.725471
H	1.173462	0.719804	-3.441859
H	2.321605	-2.040132	-4.453738
H	3.246111	-0.649185	-3.916367
H	3.482464	-2.228258	-3.138249
H	0.489103	-2.979042	-3.335312
H	1.512165	-3.336160	-1.935056
H	-0.020833	-2.488743	-1.726265
H	2.282647	-1.950430	4.522496
H	3.445625	-2.172005	3.213844
H	3.223470	-0.578504	3.965278
H	0.441598	-2.881024	3.427893
H	-0.064927	-2.418910	1.809768
H	1.456451	-3.283309	2.034046
H	0.350709	-0.499419	4.313145
H	1.174132	0.809071	3.451505
H	-0.251120	0.041857	2.745385
H	6.574761	2.238397	-0.873311
H	5.222498	1.676144	-1.860054
H	4.910783	2.588611	-0.380168
H	7.499445	-0.134556	-0.710524
H	6.478151	-1.455532	-0.122313
H	6.171568	-0.734908	-1.706771
H	7.098633	1.248055	1.307262
H	5.475278	1.562693	1.920864
H	6.165036	-0.068758	2.011826
